# miR-155 Contributes to the Immunoregulatory Function of Human Mesenchymal Stem Cells

**DOI:** 10.3389/fimmu.2021.624024

**Published:** 2021-03-26

**Authors:** Yves-Marie Pers, Claire Bony, Isabelle Duroux-Richard, Laurène Bernard, Marie Maumus, Said Assou, Frank Barry, Christian Jorgensen, Danièle Noël

**Affiliations:** ^1^ IRMB, University of Montpellier, INSERM, CHU Montpellier, Montpellier, France; ^2^ Clinical Immunology and Osteoarticular Diseases Therapeutic Unit, Department of Rheumatology, Lapeyronie University Hospital, Montpellier, France; ^3^ REMEDI, Regenerative Medicine Institute, National University of Ireland Galway, Galway, Ireland

**Keywords:** mesenchymal stromal cell, immunomodulation, miR-155, miR-221, miR-21

## Abstract

**Objectives:**

Mesenchymal stem/stromal cells (MSCs) are widely investigated in regenerative medicine thanks to their immunomodulatory properties. They exert their anti-inflammatory function thanks to the secretion of a number of mediators, including proteins and miRNAs, which can be released in the extracellular environment or in the cargo of extracellular vesicles (EVs). However, the role of miRNAs in the suppressive function of MSCs is controversial. The aim of the study was to identify miRNAs that contribute to the immunomodulatory function of human bone marrow-derived MSCs (BM-MSCs).

**Methods:**

Human BM-MSCs were primed by coculture with activated peripheral blood mononuclear cells (aPBMCs). High throughput miRNA transcriptomic analysis was performed using Human MicroRNA TaqMan^®^ Array Cards. The immunosuppressive function of miRNAs was investigated in mixed lymphocyte reactions and the delayed type hypersensitivity (DTH) murine model.

**Results:**

Upon priming, 21 out of 377 tested miRNAs were significantly modulated in primed MSCs. We validated the up-regulation of miR-29a, miR-146a, miR-155 and the down-regulation of miR-149, miR-221 and miR-361 in additional samples of primed MSCs. We showed that miR-155 significantly reduced the proliferation of aPBMCs *in vitro* and inflammation *in vivo*, using the DTH model. Analysis of miRNA-mRNA interactions revealed miR-221 as a potential target gene that is down-regulated by miR-155 both in primed MSCs and in aPBMCs.

**Conclusion:**

Here, we present evidence that miR-155 participates to the immunosuppressive function of human BM-MSCs and down-regulates the expression of miR-221 as a possible inflammatory mediator.

## Introduction

Multipotent mesenchymal stromal/stem cells (MSCs) are progenitor cells that have been identified and isolated from many tissues. Bone marrow, adipose tissue and umbilical cord are the main sources of MSCs used for diverse therapeutic applications. Along with their immunophenotype and differentiation potential, MSCs display various paracrine functions including immunomodulatory activity. MSCs can inhibit the generation and function of all types of mature immune cells through cell-cell contact and secretory mechanisms. The main molecules that have been involved in their mechanism of action are indoleamine 2,3-dioxygenase (IDO) and inducible nitric oxide synthetase (iNOS) solely in human and murine MSCs, respectively, as well as prostaglandin E2 (PGE2), interleukin-6 (IL-6), transforming growth factor β1 (TGFβ1), tumor necrosis factor-inducible gene 6 protein (TSG6), human leucocyte antigen-G5 (HLAG5) (1). Therefore, this is not a single molecule but rather a cocktail of molecules that support the immunosuppressive function of MSCs depending on the source, species and environmental context they encounter after *in vivo* implantation (2). Recent studies have shown that a large part of secreted factors, which are released in the extracellular medium or within extracellular vesicles (EVs), recapitulate most of the immunoregulatory function of MSCs (3). In addition to proteins and lipids, EV cargo contains mRNAs, lncRNAs and miRNAs, which have been identified as new players in the immunomodulatory activity of MSCs (4).

MiRNAs are short non-coding single strand RNA species of 19-23 nucleotides long that epigenetically regulate a large number of processes, such as proliferation, differentiation, metabolism, apoptosis. They act through the binding to the seed sequence within target mRNA and the repression of its translation or the induction of its degradation (5). A single miRNA can regulate many target genes while one gene can be regulated by many miRNAs making them important regulators in the core of many intracellular pathways. However, to date, a relatively little number of miRNAs have been validated as mediators of the immunoregulatory function of MSCs [for review, see (4, 6)]. Our study aimed at identifying novel miRNAs that are modulated in MSCs upon priming by inflammation and characterizing their effect on lymphocyte proliferation.

## Materials and Methods

### Isolation and Culture of Mesenchymal Stem Cells

Human bone marrow–derived MSCs (BM-MSCs) were established from bone marrow aspirates of healthy donors, after informed consent. The study was approved by the French Ministry of Research and Innovation and the Personal data Protection ethics Committee (CPP) of Languedoc-Roussillon (approval DC-2010-1185). Bone marrow was diluted in serum-free medium, filtered on a nylon membrane and centrifuged at 300 g for 10 min at room temperature. Cells were then plated at the density of 5 x 10^4^ cells/cm^2^ in α-MEM, supplemented with 10% fetal calf serum (FCS), 1 ng/mL basic fibroblast growth factor, 100 U/mL penicillin and 100 μg/mL streptomycin. When cultures reached near confluence, cells were detached with 0.05% trypsin and 0.53 mM ethylenediamine tetracetic acid, and subsequently plated at the density of 1,000 cells/cm^2^. BM-MSCs were characterized by immunophenotyping and trilineage differentiation potential as described (7) and used between passage 2 and passage 6.

### Isolation of Peripheral Blood Mononuclear Cells and Mixed Lymphocyte Reaction

Human peripheral blood mononuclear cells (PBMCs) were isolated from six buffy coats by Ficoll-Hypaque density gradient and pooled before freezing and storage at -80°C. Mixed lymphocyte reactions (MLR) were performed using PBMCs labeled with Cell-Trace Violet (CTV) according to supplier’s indications (Molecular Probes) and activated with either anti-CD3/CD28 beads (ratio 1 bead: 4 cells; Gibco) or 2.5 µg/mL phytohemaglutinin (Sigma). BM-MSCs were seeded in 96 or 24 well plates at 2x10^4^ or 8x10^4^ cells/well, respectively in proliferative medium IMDM containing 25 mM Hepes supplemented with 2 mM L-glutamine, 100 U penicillin, 100 µg streptomycin, 0.1 mM non-essential amino acids, 0.25 mM β-2-mercaptoethanol, 1 mM sodium pyruvate, and 10% inactivated FCS. Activated CTV^+^ PBMCs (aPBMCs) were added at the concentration of 2x10^5^ or 4x10^5^ cells/well either on Transwell (0.4 µm pore inserts) or directly onto MSCs (for MLR in 96 wells plates). Cocultures were maintained at 37°C, 5% CO_2_ for 96h (or 72h when indicated) before lymphocyte proliferation assessment by flow cytometry. The lymphocyte subset was first gated based on SSC and FSC parameters. The proliferation of gated CTV^+^ lymphocytes was analyzed by calculating the percentage of cells that underwent more than one cell cycle as shown by the number of CTV dilution peaks. Results are expressed as the percentage of proliferation normalized to 100% for aPBMCs.

### Analysis of miRNA Profiles

Two groups of MSCs were analyzed with the Human MicroRNA TaqMan Array Cards (TAC; ThermoFisher Scientific). One group was called naïve MSCs (MSCs) and the second group was called primed MSCs (pMSCs) when cultured in presence of aPBMCs. Total RNA was extracted using the miRNeasy Kit (Qiagen, Courtaboeuf). Quality control and quantification of total RNA were ensured with Bioanalyzer (Agilent Technologies) and NanoDrop ND-1000 (Thermo Scientific), respectively. Total RNAs were converted into cDNAs using Megaplex™RT Primers (human pool A v2.1) and TaqMan^®^ MicroRNA Reverse Transcription kit. The RT pool detected 377 miRNAs. MicroRNA profiling was achieved using the TaqMan^®^ Human MicroRNA Array Cards A v2.0 and TaqMan^®^ Master Mix. The 384-well format TACs were run on a ViiA7 real-time PCR system (ThermoFisher Scientific). RT-qPCR raw data were analyzed using SDS 2.3 and RQ Manager Software (ThermoFisher Scientific). Each miRNA for each sample was normalized to the mean threshold cycle (Ct) value of all expressed miRNAs. Comparison of the expression patterns of 377 human microRNAs in MSCs and pMSCs was performed using Ct values < 35, difference of at least 2-fold with a p-value lower than 0.05. Relative expression and statistical analysis were calculated using the ExpressionSuite software and a t test (Applied Biosciences).

### Cell Transfection

MSCs were transfected with 50 nM of premiRNAs or miRNA inhibitors using Oligofectamine™ Transfection Reagent following manufacturer’s instructions (Life Technologies) for 6 hours. The medium was then changed for FCS-containing proliferative medium and MSCs cultured for 24h. Cells were then used for RNA isolation or trypsinized and plated in TC96 or TC24 well plates, overnight. MSCs were then rinsed twice with PBS and cultured with aPBMCs under MLR conditions.

### Gene Ontology Pathway and Affymetrix Analyses

Identification of validated target genes of miRNAs was performed using TaRbase. Gene Ontology (GO) enrichment analysis was performed using Enrichr database server to identify gene enrichment in cellular component, biological process and molecular function (8). Biological pathway enrichment analysis was performed using Enrichr and Panther software (9). We used the STRING database (string-db.org) with enrichment of GO analysis to predict biological process-associated genes and REVIGO (http://revigo.irb.hr) to visualize the results.

For Affymetrix analysis, total RNA from MSCs transfected with each premiRNA or miRNA inhibitor was isolated using the miRNeasy Kit (Qiagen, Courtaboeuf) and processed for high throughput gene analysis using HG-U133 plus 2.0 GeneChip (Affymetrix) and following supplier’s recommendations. After image processing with the Affymetrix GeneChip^®^ Command Console^®^ Software (AGCC), the CEL files were analyzed using the Affymetrix Expression Console™ software v1.3.1. Data were normalized with the MAS5.0 algorithm by scaling each array to a target value (TGT) of 100 using the global scaling method to obtain an intensity value signal for each probe set. Gene annotation was performed using NetAffx database (http://www.affymetrix.com). The miRNA-mRNA interactions and biological pathways were defined using Ingenuity Pathway Analysis miRNA Target Filter for experimentally validated and putative predicted targets (high confidence level), from TargetScan, TarBase, miRecords, and the Ingenuity^®^ Knowledge Base (www.ingenuity.com).

### RNA Extraction and RT-qPCR

Total RNA was isolated using the miRNeasy Kit (Qiagen, Courtaboeuf) and either reverse-transcribed into cDNA using TaqMan^®^ MicroRNA Reverse Transcription kit (Life Technologies) for miRNA expression analysis or using M-MLV Reverse Transcriptase kit (Invitrogen) for gene expression analysis. Expression levels of miRNAs were quantified by real-time PCR using TaqMan MicroRNA Assays with Taqman Universal Master Mix II, no UNG (Life Technologies). Real-time PCR for gene expression was done using DNA Master SYBER Green I mix (Roche) and primers for *IDO1* (*Forward GCCTGATCTCATAGAGTCTGGC*; *Reverse TGCATCCCAGAACTAGACGTGC), TSG6 (Forward TCACCTACGCAGAAGCTAAGGC; Reverse TCCAACTCTGCCCTTAGCCATC*) and *TGFβ1* (*Forward ACTGCAAGTGGACATCAACGGGTTCAC; Reverse* GCCATGAGAAGCAGGAAAGGCCGGT). The expression of RNU48 and RPS9 (*Forward GATTACATCCTGGGCCTGAA, Reverse* ATGAAGGACGGGATGTTCAC) was used as endogenous gene controls for miRNA and mRNA data normalization, respectively, and results are expressed as relative expression (normalized to respective endogenous gene control) or as fold change (normalized to control sample) using the formulae 2^-ΔCt^ or 2^-ΔΔCt^, respectively.

### Analysis of Human miR-155 Targets in PBMCs Using RT^2^ Profiler PCR Array

PHA-activated and non-activated PBMCs were cultured alone or in transwell with pCT- or p155- transfected MSCs for 4 days. Total RNA was extracted and reverse transcribed (400 ng) using RT^2^ First Strand Kit according to manufacturer’s recommendations (Qiagen). qPCR was performed using RT^2^ SYBR Green Mastermix and the human miR-155 targets RT^2^ Profiler PCR array [4 x 96 well] (PAHS-6002Z, Qiagen). PCR conditions were 95°C for 10 min, followed by 45 cycles at 95°C for 15 sec, and 60°C for 1 min. Four samples were simultaneously analyzed on a 384-well PCR array. Expression of B2M was used as endogenous control for data normalization.

### ELISA Assays

Different cytokines (HGF, IL-6, TGFβ1) were quantified in the supernatants from MSCs:aPBMCs cocultures. Supernatants were collected and stored at -20°C until quantification using DuoSet^®^ ELISA kits from R&D Systems (Biotechne) and prostaglandin E2 (PGE2) using Multi-format ELISA kit from Arbor Assays.

### DTH Assay

Groups of 8 C57BL/6JRj female aged 7 weeks were immunized by intradermic injection of methyl bovine serum albumin (mBSA) emulsified in Freund’s complete adjuvant at the base of the tail (150 µg/100 µL/mouse) at day 0. Five days later, mice were challenged with 30 µg mBSA/30 µL saline solution in the right hind footpad (control mice). In treated groups, a suspension of 30 µg mBSA/30 µL saline solution containing 30,000 cells was injected in the right footpad of mice. All mice received 30 µL saline solution in the left footpad as a negative control. Inflammation was evaluated by measuring the footpad swelling using a caliper the day before mouse immunization (day -1) and 24 hours after the boost (day 6). Thickness increment of footpads was calculated as: (right footpad thickness-left footpad thickness at day 6) – (right footpad thickness-left footpad thickness at day -1).

### Statistical Analysis

Statistical analysis was performed using the GraphPad Prism software version 7.0 (GraphPad Software Inc). Analysis of data distribution was performed using a Shapiro-Wilk normality test. When the test passed normality, statistical analysis used either a paired- or unpaired-t test; otherwise a Wilcoxon matched-pairs signed rank test or a Mann-Whitney-t test were used for 2-group comparisons (as indicated in figure legends). P values <0.05 were considered statistically significant.

## Results

### Identification of Several miRNAs Modulated in pMSCs

**Figure 1:**
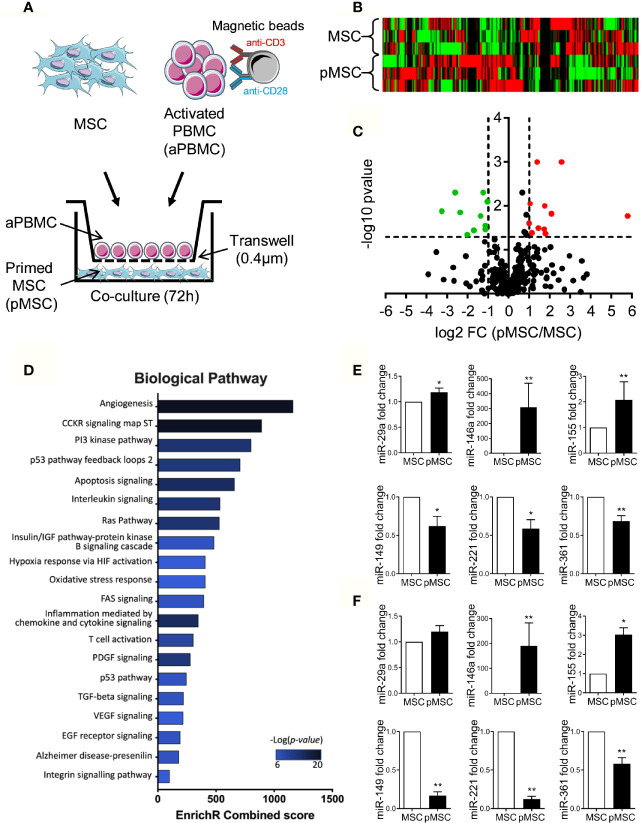
Identification of several miRNAs modulated in pMSCs. **(A)** Experimental scheme for miRNA analysis in naïve MSCs and MSCs primed (pMSCs) by activated peripheral blood mononuclear cells (aPBMCs) (n=3 biological replicates). **(B)** Heat map analysis of naïve MSCs and pMSCs depending on 377 miRNA expression. **(C)** Volcano plot analysis of miRNAs expressed by naïve MSCs and pMSCs and shown in red for significantly up-regulated miRNAs and in green for significantly down-regulated miRNAs. **(D)** Gene Ontology enrichment analysis of biological pathways for target genes of the top 10 modulated miRNAs (fold change>2.5). **(E)** Mean fold change ± SEM of six miRNAs selected in **(C)** in additional samples of naïve MSCs or pMSCs (MSCs + anti-CD3/CD28 antibodies-activated PBMCs) (n=5-9 biological replicates). **(F)** Mean fold change ± SEM of miRNAs in additional samples of naïve MSCs or pMSCs by PHA-activated PBMCs (n=5-9 biological replicates). Statistical analysis used Wilcoxon matched-pairs Signed Rank Test or paired-t test with *p < 0.05; **p < 0.01.

With the rationale that miRNAs supporting the immunosuppressive function of MSCs are modulated when MSCs are primed by activated immune cells, we compared the miRNome of naïve MSCs and pMSCs. Three individual MSCs were primed by coculture with aPBMCs seeded on a Transwell insert to avoid any contact, for 72 h before miRNA identification and quantification by TAC analysis (**Figure 1A**). Using a heat map analysis, we confirmed that miRNA expression was differently regulated in pMSCs compared to MSCs (**Figure 1B**). The miRNAs that were differently expressed between MSCs and pMSCs were identified using a volcano plot analysis (**Figure 1C**). The log2 of the fold change for each miRNA in pMSCs versus MSCs was plotted versus the corresponding log10 of p-value. The threshold of fold change in expression ≥2.0 and p < 0.05 was used to screen up- or downregulated miRNAs. We identified 11 and 10 miRNAs that were, respectively, up- and down-regulated in pMSCs in comparison to MSCs (**Table 1**). The top 10 miRNAs differentially regulated (with a fold change >2.5) were selected to perform a functional enrichment analysis on experimentally validated target genes. Using miRTarBase, 243 target genes were identified and analyzed for biological pathway enrichment using Enrichr software. Overrepresented biological pathways were related to inflammation, proliferation and apoptosis (**Figure 1D**). GO Term Enrichment Analysis was also used to classify target genes into distinct functional categories, including biological processes (**Supplementary Table 1**), molecular functions (**Supplementary Table 2**) and cellular components (**Supplementary Table 3**).

**Table 1:** List of miRNAs modulated in pMSCs.

Up-regulated microRNAs		
Mature miRNA	Fold change pMSCs/MSCs	p-value
hsa-miR-146a	55,55	0,017
hsa-miR-184	5,95	0,001
hsa-miR-545	4,22	0,015
hsa-miR-518d	3,45	0,043
hsa-miR-146b-3p	3,38	0,010
hsa-miR-302b	3,33	0,034
hsa-miR-29b	2,74	0,032
hsa-miR-155	2,60	0,001
hsa-miR-652	2,18	0,041
hsa-miR-29c	2,07	0,009
hsa-miR-29a	1,99	0,025
**Down-regulated microRNAs**		
**Mature miRNA**	**Fold change pMSCs/MSCs**	**p-value**
hsa-miR-149	-3,25	0,013
hsa-miR-221	-2,6	0,005
hsa-miR-199a	-2,36	0,014
hsa-miR-758	-2,01	0,045
hsa-miR-145	-1,68	0,036
hsa-miR-27a	-1,36	0,017
hsa-miR-361	-1,24	0,005
hsa-miR-27b	-1,13	0,034
hsa-miR-23a	-1,12	0,028
hsa-miR-345	-1,03	0,008

We then selected six miRNAs that were among the highly modulated in terms of expression change or the most statistically significant (lowest p-values). Using similar coculture conditions as in **Figure 1A** and RT-qPCR analysis, we validated a significantly higher expression of the mature forms of miR-29a (miR-29a-3p), miR-146a (miR-146a-5p), miR-155 (miR-155-5p) and lower expression of miR-149 (miR-149-5p), miR-221 (miR-221-3p), miR-361 (miR-361-3p), respectively, in additional samples of pMSCs compared to naïve MSCs (**Figure 1E**). Similar modulation of miRNA expression was also observed using the PHA mitogen instead of CD3/CD28 beads to activate PBMCs (**Figure 1F**). These data confirmed that expression of at least these six miRNAs was significantly modulated upon MSC priming.

### miR-155 Regulates a Large Number of Target Genes in pMSCs

**Figure 2:**
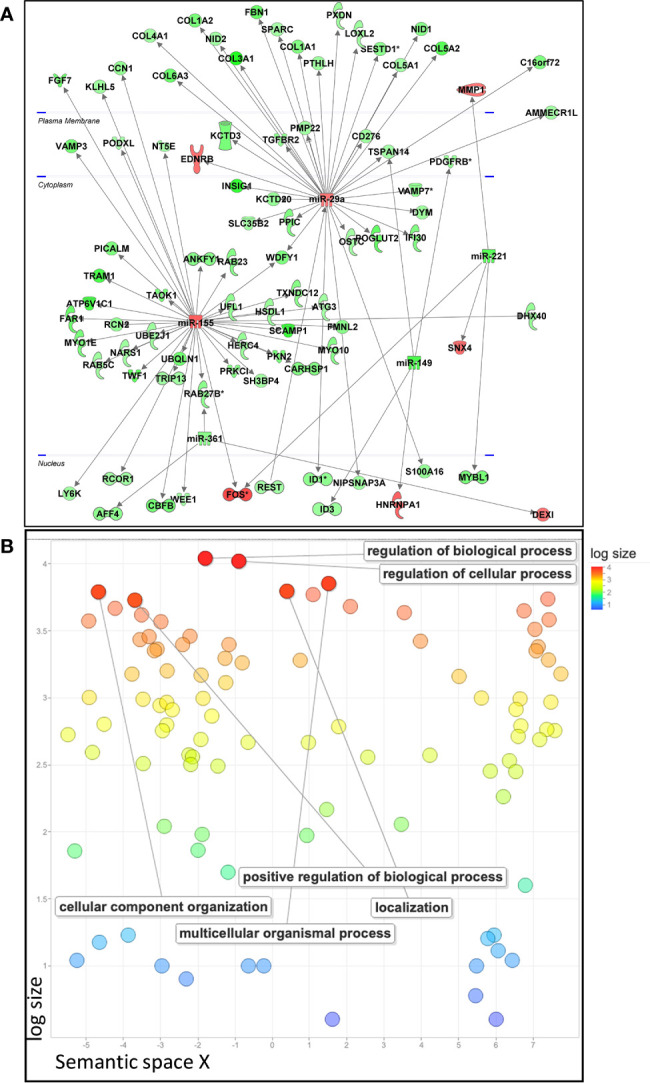
Analysis of predicted miRNA*-*mRNA interactions by *IPA’s miRNA Target Filter*. **(A)** The resultant differentially expressed genes by over-expression of miR-155, miR-146a and miR-29a and down-expression of miR-149 and miR-361, reflecting the immunosuppressive conditions of MSCs, were uploaded on the IPA software to identify putative targets and significant biological functions. **(B)** Gene Ontology scatterplot generated with REVIGO for all the GO terms associated with modulated genes reflecting the immunosuppressive conditions of pMSCs and identified using STRING database. Settings used for REVIGO program were as follow: *Homo sapiens* database, SimRel as semantic similarity measure, the allowed similarity was medium (0.7) between the enriched GO terms.

To identify the potential targets of the selected immunosuppressive miRNAs in pMSCs, we performed a high-throughput gene analysis by over- or under-expressing each miRNA, except for miR-221, which was not modulated using specific premiRNA or miRNA inhibitor. We identified numerous genes whose expression was inversely modulated to that of miRNAs, namely 249 up- and 1029 down-regulated genes (**Supplementary Table 4**). We then analyzed the predicted miRNA-mRNA interactions using the IPA’s miRNA Target Filter and uploaded the genes modulated by over-expression of miR-155, miR-146a, miR-29a and down-expression of miR-149, miR-361, reflecting the immunosuppressive conditions of our experimental design (**Figure 1A**). Two miRNAs, miR-29a and miR-155, were central in the regulation of numerous direct target genes, in contrast to miR-146a, miR-149 and miR-361 (**Figure 2A**). Using STRING database and REVIGO software, we constructed a gene ontology scatterplot for all the GO terms identified for the genes modulated by the five miRNAs and found out that biological and cellular processes as well as cellular component organization were among the highly regulated pathways (**Figure 2B**). Actually, apart from the central role played by miR-29a and miR-155 on gene regulation, the analysis did not provide any significant pathway associated with inflammation or immune responses.

### MiR-155 Is Involved in the Immunosuppressive Function of MSCs

**Figure 3:**
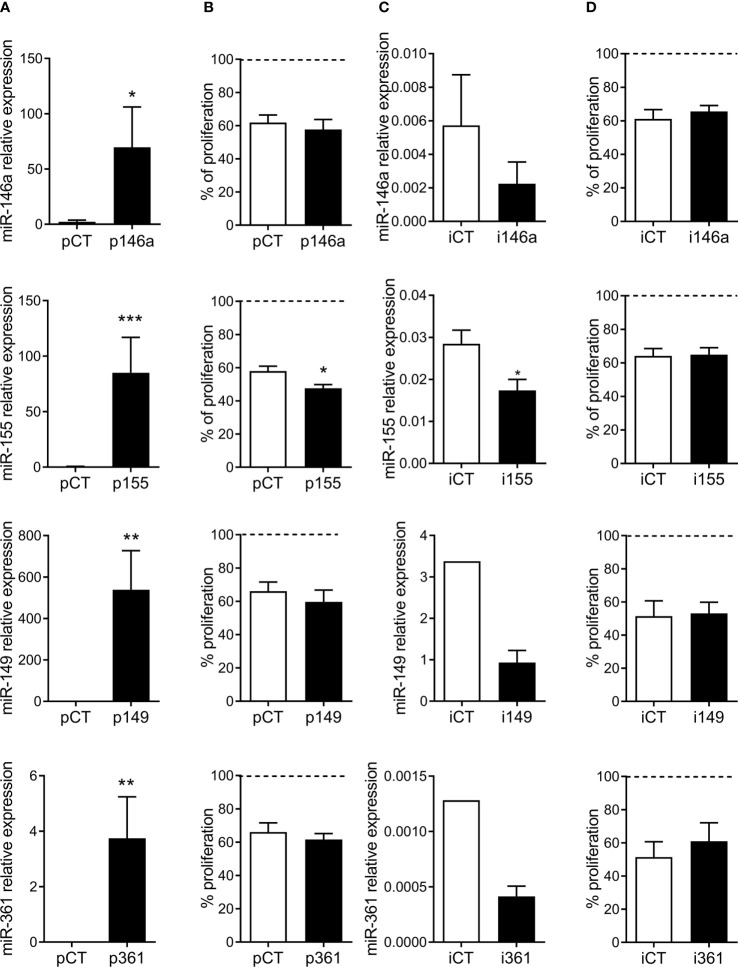
MiR-155 is involved in the immunosuppressive function of MSCs. **(A)** Relative expression of miR-146a, miR-155, miR-149 and miR-361 in MSCs after transfection of specific premiRNAs (p146a, p155, p149, p361) or premiRNA control (pCT) (n=4-6 biological replicates). **(B)** Percentage of proliferation of aPBMCs cultured in presence of MSCs transfected with the indicated premiRNAs (n=5-6 biological replicates). **(C)** Relative expression of miR-146a, miR-155, miR-149 and miR-361 in MSCs after transfection of specific miRNA inhibitors (i146a, i155, i149, i361) or control RNA inhibitor (iCT) (n=3-4 biological replicates). **(D)** Percentage of proliferation of aPBMCs cultured in presence of MSCs transfected with the indicated miRNA inhibitors (n=3-5 biological replicates). Results are expressed as percentage of aPBMCs maximal proliferation set at 100%. Statistical analysis used Mann-Whitney test with *p < 0.05; **p < 0.01, ***p < 0.001.

We therefore evaluated the role of these miRNAs in the immunomodulatory function of pMSCs. The miRNAs were either over- or down-regulated in MSCs, which were then cocultured with aPBMCs for four days. Results obtained with miR-29a are reported elsewhere (submitted manuscript). The four other miRNAs were highly up-regulated in MSCs using specific premiRNAs or down-regulated using specific miRNA inhibitors (**Figures 3A, C**). However, only miR-155 was able to significantly increase the immunosuppressive function of pMSCs, as shown by the lower percentage of proliferative lymphocytes (**Figure 3B**). The down-regulation of miRNAs using specific inhibitors was not sufficient to detect a significant effect on the proliferation of lymphocytes, after the four-day culture period (**Figure 3D**).

**Figure 4:**
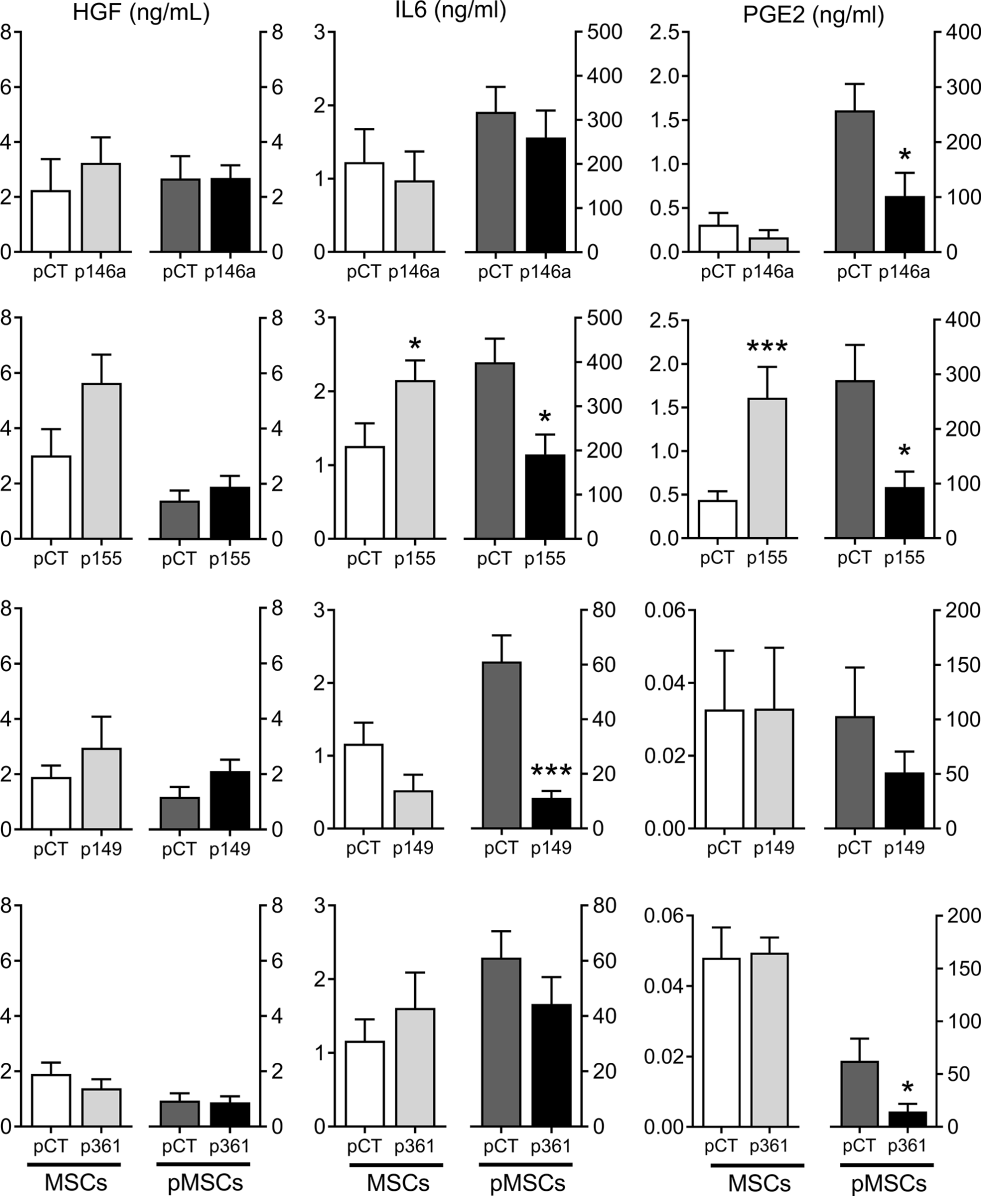
Production of known immunosuppressive factors by MSCs over-expressing miRNAs. Protein levels of HGF, IL6, PGE2 in the supernatants of MSCs cultured alone (MSCs) or pMSCs:aPBMC cocultures (pMSCs), using premiRNA control (pCT) or p146a-, p155-, p149-, p361- transfected MSCs, determined by ELISA (n=6-10 biological replicates). Statistical analysis used unpaired-t or Mann-Whitney test when data assumed normality or not, respectively, with *p < 0.05; ***p < 0.001.

**Figure 5:**
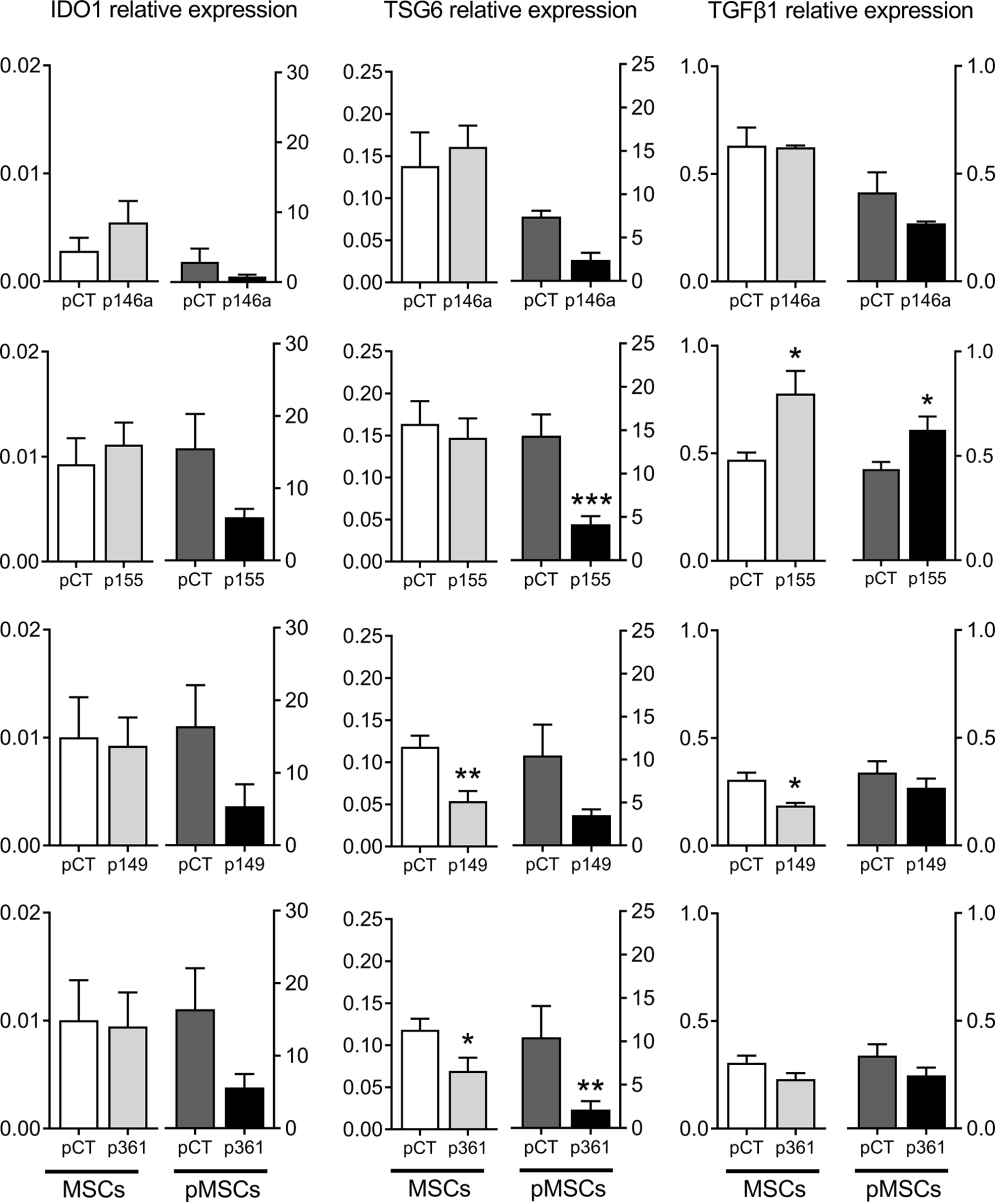
Production of known immunosuppressive factors by MSCs over-expressing miRNAs. Mean relative expression of mRNA levels ± SEM of IDO1, TSG6, TGFβ1 in MSCs cultured alone (MSCs) or in pMSCs from pMSCs:aPBMC cocultures, determined by RT-qPCR (n=6-10 biological replicates). Statistical analysis used unpaired-t or Mann-Whitney test when data assumed normality or not, respectively, with *p < 0.05; **p < 0.01; ***p < 0.001.

We wondered whether the up-regulation of these miRNAs in MSCs can modulate the expression of known immunosuppressive factors in MSCs cultured alone or with aPBMCs. The protein levels in the supernatants of transfected MSCs cultured alone were stable for HGF, IL6 and PGE2, except for IL6 and PGE2, which increased after transfection with the premiR-155 (**Figure 4**). In the supernatants from pMSCs:aPBMCs cocultures, HGF amounts remained unchanged while the amounts of IL6 and PGE2 highly increased in the cocultures. Nevertheless, the amounts of IL6 and PGE2 decreased in premiRNA-transfected pMSCs compared to premiRNA control. The decrease of IL6 was significant in coculture supernatants from pMSCs transfected with premiR-155 and premiR-149 while PGE2 was significantly decreased when pMSCs were transfected with premiR-146a, premiR-155 and premiR-361. As expected, the mRNA levels of IDO1 and TSG6 were increased in pMSCs in cocultures compared to MSCs cultured alone (**Figure 5**). The expression levels of all three factors were stable in MSCs transfected with the premiRNAs and cultured alone, except for TSG6, which significantly decreased in MSCs transfected with premiR-149 or premiR-361 and, TGFβ1, which increased in MSCs transfected with premiR-155 and decreased in MSCs transfected with premiR-149. In the coculture conditions, all factors tended to decrease in pMSCs transfected with premiRNAs. Significant down-regulation of TSG6 was found in pMSCs transfected with premiR-155 and premiR-361. Of note, TGFβ1 was significantly increased in premiR-155-transfected pMSCs. Overall, the results indicated that the higher immunosuppressive function of premiR-155-transfected pMSCs could partly be attributed to an increased secretion of TGFβ1 but not other known immunosuppressive factors.

**Figure 6:**
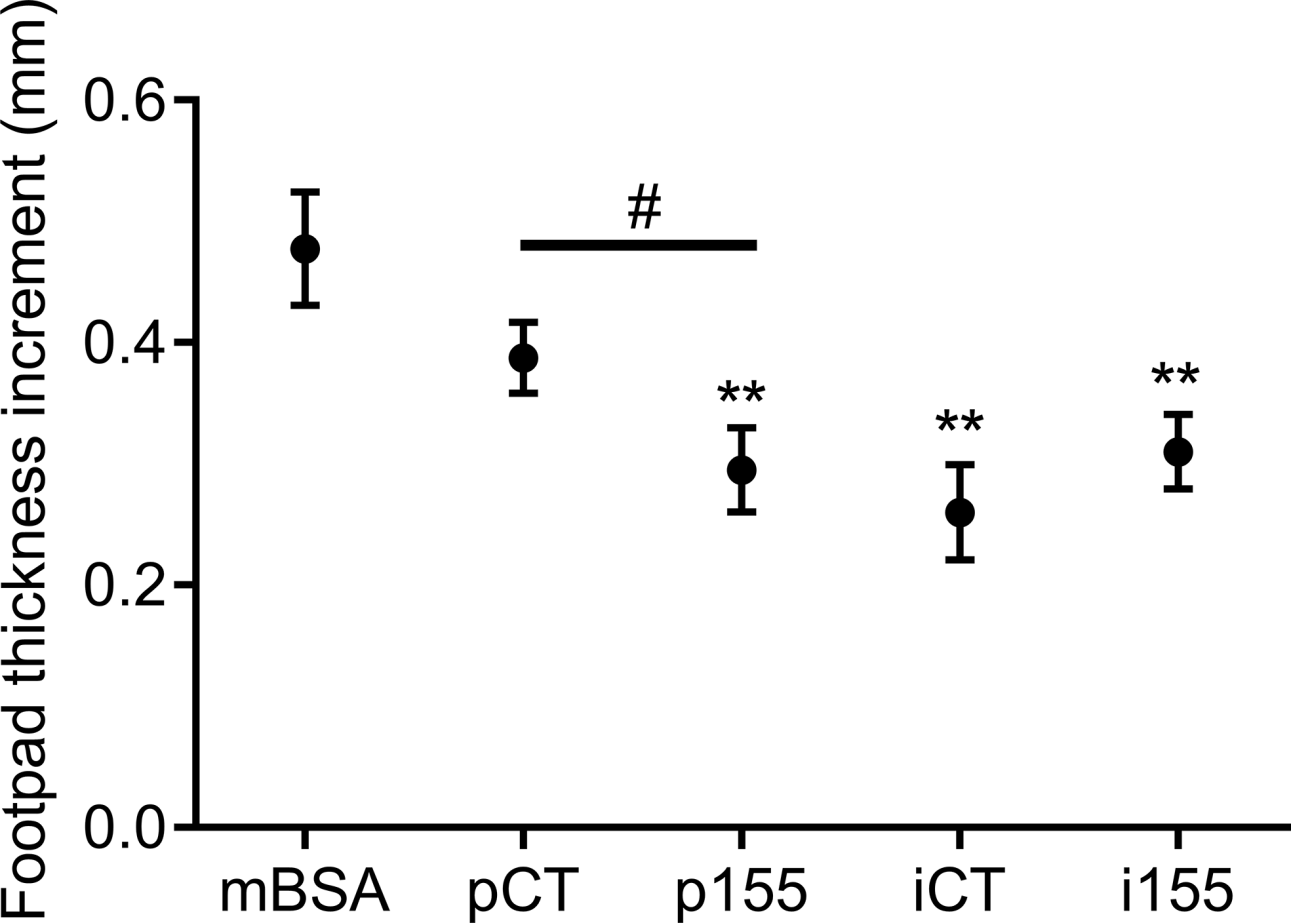
Over-expression of mir-155 enhances the immunosuppressive function of MSCs in the DTH murine model. Mice were immunized by intradermal injection of methyl bovine serum albumin (mBSA) at day 0 and challenged by injection of mBSA ± MSCs transfected with pCT, p155, iCT or i155 in the footpad at day 5. Footpad thickness increment was determined after 24h. Statistical analysis used unpaired-t test with **p < 0.01 versus mBSA group and ^#^p < 0.05 between indicated groups.

Finally, we investigated the immunosuppressive function of miR-155 *in vivo*, by over- and under-expressing miR-155 in MSCs. We used the DTH murine model and injected the modified cells in the footpad of mBSA-immunized mice. The DTH response was determined by measuring the footpad thickness increment. In control immunized mice, inflammation was characterized by high footpad thickness while footpad thickness was reduced in all mice receiving MSCs (**Figure 6**). However, p155-transfected MSCs significantly reduced inflammation compared to pCT-transfected MSCs. MSCs transfected with i155 were less immunosuppressive than iCT-transfected MSCs although the difference did not reach significance. The results therefore confirmed that over-expression of premiR-155 in MSCs increased their immunosuppressive potential.

### MiR-155 Contributes to the Down-Regulation of miR-221

**Figure 7:**
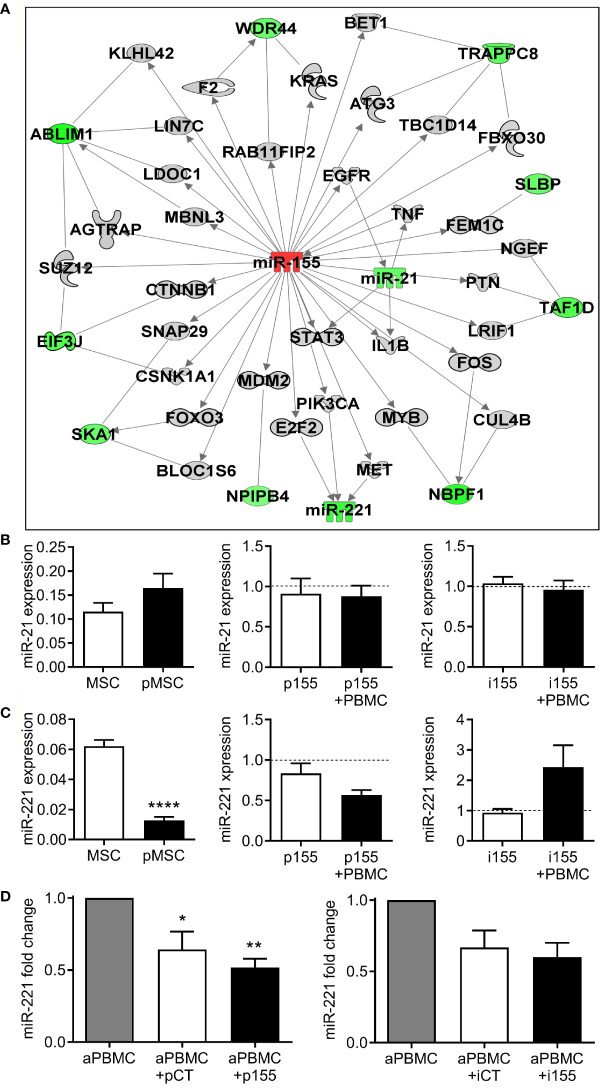
miR-221 is indirectly modulated by miR-155. **(A)** Integration of *miRNA-targets* following miR-155 over-expression. Green and red shapes represent genes/miRNA down- and up-regulated, respectively and grey shapes are other genes, not modulated in the transcriptomic analysis, but with putative links. Solid lines mean direct or putative interaction. **(B)** Mean relative expression ± SEM of miR-21 in naïve MSCs and in pMSCs primed by coculture with aPBMCs (MSC+PBMC) (n=11; left panel) and in MSCs transfected with premiR-155 (p155; n=4; middle panel) or miR-155 inhibitor (i155; n=3; right panel) and cultured alone or with PBMCs. Expression is normalized to MSCs transfected with control premiRNA (pCT) or miRNA inhibitor (iCT) (dash line). **(C)** Mean expression ± SEM of miR-221 in naïve MSCs and in MSCs primed by coculture with aPBMCs (MSC+PBMC) (n=11; left panel) and in MSCs transfected with p155 (n=4; middle panel) or i155 (n=3; right panel) and cultured alone or with PBMCs. Expression is normalized to MSCs transfected with control premiRNA or miRNA inhibitor (dash line). **(D)** Mean expression ± SEM of miR-221 in aPBMCs when cultured alone or with MSCs transfected with control premiRNA (pCT) or p155 (n=4; left panel) or with control miRNA inhibitor (iCT) or i155 (n=3; right panel). Statistical analysis used paired-t test (left panels in **B, C**), Wilcoxon unpaired signed rank test (**D** and middle and right panels in **B, C** as compared to pCT or iCT) with *p < 0.05; **p < 0.01; ****p < 0.0001.

We therefore focused our attention on miR-155 associated to the immunosuppressive effect of pMSCs and analyzed again the potential target genes that could be modulated when up- or down-regulated. Using the IPA’s miRNA Target Filter and the data from the Taqman MicroArray and Affymetrix analyses (**Supplementary Table 4**), we performed a novel miRNA-mRNA interaction analysis and included genes that can be indirectly modulated by miR-155 expression modulation. We found that several genes were down-expressed in premiR-155 overexpressing MSCs (**Figure 7A**). Interestingly, we found that miR-221 and miR-21 were two miRNAs whose expression was decreased when miR-155 was up-regulated. In the analysis, miR-21 was downstream of STAT3 and EGFR while miR-221 was downstream of E2F2, MET and PIK3CA. We determined that miR-21 was up-regulated in pMSCs (**Figure 7B**). However, pMSCs transfected with premiR-155 or miR-155 inhibitor expressed similar levels of miR-21 suggesting that in our conditions, miR-21 was not a target of miR-155. By contrast, the expression of miR-221 was significantly decreased in pMSCs and in pMSCs transfected with premiR-155 (**Figure 7C**). Inversely, the down-regulation of miR-155 using miR-155 inhibitor increased the expression of miR-221 in pMSCs. We also observed that expression of miR-221 was decreased in aPBMCs when cultured in presence of pMSCs (**Figure 7D**). Addition of pMSCs transfected with premiR-155, but not with miR-155 inhibitor, further decreased miR-221 expression. Therefore, our results suggest that miR-221 is one indirect target of miR-155 whose down-regulation may play a role in the immunosuppressive function of pMSCs.

Finally, we wanted to determine whether miR-155 over-expressed in MSCs would be released in PBMCs upon EV uptake, thereby repressing target genes important for immune functions. Using a premiR-155 target specific array, we assessed the target gene expression in aPBMCs that have been cocultured with premiR-155 over-expressing MSCs and in PHA-activated PBMCs. We found that few genes were modulated in PBMCs after coculture with premiR-155 overexpressing MSCs, the top modulated ones being up-regulated (**Supplementary Table 5**). In PHA-activated PBMCs, where miR-155 expression was increased by a 20-fold factor, many genes were up- and down-regulated (**Supplementary Table 5**). From these data, we sorted out a short list of up-regulated genes in PBMCs cocultured with premiR-155 over-expressing MSCs and of down-regulated genes in PHA-activated PBMCs, as representative of genes potentially related to immunosuppressive function (**Table 2**). Interestingly, MAFB and SPI1, which are involved in monocyte/macrophage activation, and CEBPB, which inhibits T cell proliferation and activation were modulated in both situations. Furthermore, NFATC2IP, a regulator of anti-inflammatory cytokine production by Th2 lymphocytes and TP53INP1, an anti-proliferative protein were up-regulated in aPBMCs cocultured with premiR-155 overexpressing pMSCs. The data therefore suggest that miR-155 over-expressed in pMSCs could modulate the expression of several genes involved in inflammation and switch the immune response toward an anti-inflammatory response.

**Table 2:** Short list of target genes modulated in PBMCs after coculture with premiR-155 overexpressing MSCs or PHA activation.

Gene	p155/pCT ratio	Gene	A/NA ratio
MAFB	1,69	BACH1	0,76
BCORL1	1,59	ZNF652	0,71
SPI1	1,56	INFGR1	0,67
HNRNPA3	1,41	LPAR6	0,39
NFATC2IP	1,33	IRAK3	0,29
STX16	1,32	CEBPB	0,24
CEBPB	1,32	TSHZ3	0,22
RUNX2	1,31	MAFB	0,13
TP53INP1	1,30	EDN1	0,13
		SPI1	0,07

The cut-off ratio was ≥1.3 for genes up-modulated in PBMCs after coculture with premiR-155 overexpressing MSCs (p155) on premiR-control overexpressing MSCs (pCT) and <0.8 for genes down-modulated in PHA-activated PBMCs (A) on non-activated PBMCs (NA). In grey are the target genes validated experimentally and in blank, the putative target genes.

## Discussion

In the present study, we have identified several miRNAs whose expression was modulated in pMSCs in presence of aPBMCs suggesting that they could play a role in the immunosuppressive function of MSCs. We identified the miR-155/miR-221 axis as a new player in the immunoregulatory function of pMSCs.

Several miRNAs were previously reported to modulate the immunosuppressive function of MSCs (6). All the down-regulated miRNAs described in **Table 1**, except for miR-23a, have already been reported to be down-regulated in human foreskin MSCs activated by an inflammatory cytokine cocktail but their role was not investigated (10). Among these, miR-27b was shown to negatively modulate the immunosuppressive function of rat adipose tissue-derived MSCs and miR-27a was reported as a positive regulator of M2 macrophage polarization (11, 12). In our study, the most up-regulated miRNA in pMSCs was miR-146a. MiR-146a was one of the first miRNAs shown to negatively regulate the immunosuppressive function of murine BM-MSCs by targeting PGE2 synthase 2 (13). Indeed, inhibition of miR-146a in BM-MSCs was shown to decrease the proliferation of T lymphocytes. Consistently, it was reported that human adipose tissue-derived MSCs expressing lower amounts of miR‐146a exhibited enhanced immunomodulatory properties, as shown by increased production of key immunosuppressive factors and production of lower amounts of inflammatory genes by macrophages (14). By contrast, miR-146a was the most significantly up-regulated in TNFα-stimulated human umbilical cord MSCs even though no functional assay confirmed a possible immunosuppressive role (15). Recently, exosomes from MSCs over-expressing miR-146a were demonstrated to increase Treg cell populations and improve collagen-induced arthritis (16). In the present study, we did not observe any effect on the proliferation of T lymphocytes. The obvious contradictory results likely reflect the environmental context of the studies, which relied on different types of MSC priming, different immune cell responses investigated and different sources and species of MSCs. The expression levels of pro- and anti-inflammatory miRNAs can vary with the species, source, culture conditions of MSCs thus leading to distinct global effects on the immunosuppressive role of MSCs.

We validated functionally miR-155 as being a positive regulator of the immunosuppressive function of human pMSCs. This miRNA was first reported to inhibit the immunosuppressive activity of murine BM-MSCs by suppressing iNOS production (17). Since then, the enhanced expression of miR-155 in primed rat BM-MSCs promoted the differentiation of naïve T cells to Treg and anti-inflammatory Th2 populations while inhibiting the differentiation toward Th1 and Th17 inflammatory sub-populations (18). This effect was proposed to result from miR-155-mediated repression of suppressor of cytokine signaling 1 (SOCS1), which is a negative regulator of the IL2 signaling cascade (19). MiR-155 control of IL2 signaling is mandatory for Treg cell survival. Moreover, Foxp3-dependent upregulation of high levels of miR-155 in Treg cells maintains Treg cell homeostasis by targeting SOCS1, in a context- and cell type-dependent manner (20). Our results are in line with these studies and highlight the importance of the cell context, here the species of origin. Indeed, iNOS is not expressed and therefore cannot be modulated by miR-155 in rat and human MSCs. In non-murine MSCs, miR-155 likely regulates the expression of multiple other target genes resulting in an overall immunosuppressive activity. This activity did not result from an increased production of key immunosuppressive mediators since several factors, including IL6, PGE2 and TSG6 were down-regulated by miR-155 over-expression. Nevertheless, TGFβ1 was increased in miR-155-overexpressing pMSCs, which suggests that it could participate to their higher immunoregulatory function by inducing Treg cells. A recent study identified miR-155 in extracellular vesicles isolated from pMSCs and demonstrated that over-expression of miR-155 in B lymphocytes negatively impacted their survival (21). Although the effect of EV-mediated release of miR-155 was not investigated on their immune function, the results suggest that miR-155 participates to the immunosuppressive function of MSCs. In the present study, we further identified several target genes that were up-regulated in aPBMCs when cocultured with premiR-155 overexpressing pMSCs. Although these data were performed on the entire immune cell population, we nevertheless identified SPI1 and MAFB upregulation in aPBMCs, which might be related to macrophage activation and differentiation toward a M2 anti-inflammatory phenotype (22, 23). We also detected the up-regulation of NFATC2IP, which is involved in Th2 cell differentiation and anti-inflammatory cytokine production (24) and CEBPB, whose anti- or pro-proliferative function depends on the cell subset and/or context (25). Up-regulation of the pro-apoptotic TP53INP1 also suggests an inhibitory role on immune cell proliferation (26). These preliminary results require additional experiments using purified immune cell subsets to decipher the precise role of miR-155 in distinct immune cell subtypes but set the basis for future studies.

We identified two miRNA targets that were putatively negatively regulated by miR-155, namely miR-21 and miR-221, and validated the down-regulation of miR-221 when miR-155 was up-regulated. MiR-21 was recently shown to be packaged within EVs produced by human MSCs and to play a role in their immunosuppressive function by repressing T-cell receptor (TCR) and TLR4 signaling on murine splenocytes (27). However, in a previous study, miR-21 negatively regulated the immunosuppressive role of murine BM-MSCs by targeting phosphatase and tension homolog deleted on chromosome 10 (PTEN) and up-regulating downstream AKT/NF-κB pathway, leading to lower amounts of TGFβ1 and decreased number of Treg cells (28). Again, differential effect of miR-21 on immune responses was observed depending on the species of MSCs. In our experimental conditions, we detected the up-regulation of miR-21 in pMSCs but did not observe the modulation of miR-21 by miR-155, indicating that miR-21 was not involved in the regulatory function of miR-155. By contrast, we found that miR-221 and miR-155 expressions were inversely correlated and miR-221 was down-regulated both in pMSCs and aPBMCs in cocultures, suggesting an inhibitory effect on inflammatory responses. In MSCs, miR-221 has been associated to anti-chondrogenic, anti-osteogenic and pro-angiogenic activities but its modulatory function has not been investigated (29–31). Mir-155 directly targets E2F2, which induces miR-221 expression and down-regulates cyclin-dependent kinases, thereby regulating cell proliferation (32, 33). Interestingly, an inversed correlation between miR-155 and miR-221 was recently reported in TLR4-stimulated M1 and M2 macrophages (34). In this settings, high levels of miR-221 shifted M2 macrophages toward a pro-inflammatory M1 phenotype by targeting directly JAK3 and downstream pSTAT3 activation and IL10 secretion. Other studies reported miR-221-dependent down-regulation of PI3Kα that mediates IFNγ-induced IDO activity in MSCs (35, 36). MiR-155-mediated repression of miR-221 therefore favors anti-inflammatory responses through JAK/STAT and PI3K/AKT signaling.

In conclusion, we present evidence that miR-155 contributes to the immunosuppressive function of human BM-MSCs and down-regulates the expression of miR-221 in BM-MSCs and in target PBMCs. We further discuss cell type- and species-specific regulation of miRNA/target gene interactions that may explain the contradictory results frequently reported on the immunoregulatory role of miRNAs expressed by MSCs.

## Data Availability Statement

The datasets presented in this study can be found in online repositories. The names of the repository/repositories and accession number(s) can be found in the article/**Supplementary Material**.

## Ethics Statement

The studies involving human participants were reviewed and approved by French Ministry of Research and Innovation and the Personal data Protection ethics Committee (CPP) of Languedoc-Roussillon (approval DC-2010-1185). The patients/participants provided their written informed consent to participate in this study. The animal study was reviewed and approved by Regional Ethics Committee on Animal Experimentation (APAFIS#5352-20l6050918509993v3).

## Author Contributions

DN and CJ designed the experiments. Experimental work was performed by Y-MP, CB, and LB. Y-MP, CB, ID-R, MM, SA, FB, and DN analyzed the data and prepared the manuscript. All authors contributed to the article and approved the submitted version.

## Funding

We gratefully acknowledge the Agence Nationale pour la Recherche for support of the national infrastructure: “ECELLFRANCE: Development of a national adult mesenchymal stem cell based therapy platform” (ANR-11-INSB-005). The study was also supported by the European Union Horizon 2020 Programme (project RESPINE, grant agreement #: 732163). The materials presented and views expressed here are the responsibility of the authors only. The EU Commission takes no responsibility for any use made of the information set out. Funding for staff exchange was received by Programme Ulysses 2018 (project #: P41059WK).

## Conflict of Interest

The authors declare that the research was conducted in the absence of any commercial or financial relationships that could be construed as a potential conflict of interest.
