# Supplementary material for: miR-155 Contributes to the Immunoregulatory Function of Human Mesenchymal Stem Cells
Source: Front Immunol. 2021 Mar 26;12:624024. doi: 10.3389/fimmu.2021.624024 (PMC8033167; doi:10.3389/fimmu.2021.624024)
Supplement: Supplementary file 1 [file Table_1.docx]

**Supl. Table 1**: Gene Ontology enrichment analysis of biological processes for miR-155 target genes in pMSCs versus MSCs

| positive regulation of fibroblast proliferation | 1744 | 1,2E-12 |
| --- | --- | --- |
| negative regulation of platelet-derived growth factor receptor-beta signaling pathway | 1542 | 1,5E-05 |
| positive regulation of phospholipase activity | 1467 | 1,6E-09 |
| regulation of phospholipase C activity | 1364 | 1,8E-06 |
| macrophage differentiation | 1096 | 3,1E-06 |
| regulation of mesenchymal cell proliferation | 908 | 4,8E-06 |
| collagen fibril organization | 898 | 2,2E-09 |
| regulation of fibroblast proliferation | 869 | 1,8E-11 |
| regulation of platelet-derived growth factor receptor-beta signaling pathway | 842 | 4,2E-05 |
| positive regulation of mitochondrial membrane potential | 837 | 3,7E-04 |
| response to UV-A | 837 | 3,7E-04 |
| negative regulation of glial cell proliferation | 837 | 3,7E-04 |
| regulation of osteoclast development | 837 | 3,7E-04 |
| negative regulation of extrinsic apoptotic signaling pathway via death domain receptors | 793 | 3,9E-09 |
| positive regulation of blood vessel endothelial cell migration | 708 | 3,0E-10 |
| extracellular matrix organization | 680 | 5,3E-20 |
| negative regulation of cell activation | 674 | 6,4E-05 |
| regulation of cyclin-dependent protein serine/threonine kinase activity involved in G1/S transition of mitotic cell cycle | 665 | 1,1E-05 |
| positive regulation of pri-miRNA transcription from RNA polymerase II promoter | 625 | 2,1E-06 |
| positive regulation of protein serine/threonine kinase activity | 595 | 3,2E-14 |
