# Supplementary material for: miR-155 Contributes to the Immunoregulatory Function of Human Mesenchymal Stem Cells
Source: Front Immunol. 2021 Mar 26;12:624024. doi: 10.3389/fimmu.2021.624024 (PMC8033167; doi:10.3389/fimmu.2021.624024)
Supplement: Supplementary file 2 [file Table_2.docx]

**Supl. Table 2**: Gene Ontology enrichment analysis of molecular function for miR-155 target genes in pMSCs versus MSCs

| **Term** | **EnrichR Combined Score** | **Adjusted P-value** |
| --- | --- | --- |
| platelet-derived growth factor binding | 3652 | 5,3E-10 |
| platelet-derived growth factor receptor binding | 1353 | 1,1E-07 |
| protein-lysine 6-oxidase activity | 837 | 3,5E-04 |
| mismatched DNA binding | 452 | 8,3E-04 |
| mismatch repair complex binding | 452 | 8,3E-04 |
| R-SMAD binding | 377 | 3,8E-05 |
| siRNA binding | 361 | 1,2E-03 |
| inositol tetrakisphosphate phosphatase activity | 361 | 1,2E-03 |
| phosphatidylinositol-4,5-bisphosphate 3-kinase activity | 344 | 2,1E-08 |
| phosphatidylinositol bisphosphate kinase activity | 320 | 2,8E-08 |
| collagen binding | 318 | 2,5E-07 |
| I-SMAD binding | 297 | 1,5E-03 |
| phosphatidylinositol 3-kinase activity | 284 | 5,3E-08 |
| transcription regulatory region DNA binding | 281 | 6,6E-14 |
| protein kinase binding | 263 | 1,9E-14 |
| Y-form DNA binding | 249 | 1,2E-02 |
| DNA insertion or deletion binding | 249 | 1,2E-02 |
| RNA polymerase II core promoter proximal region sequence-specific binding | 220 | 4,6E-11 |
| transcriptional repressor activity, RNA polymerase II activating transcription factor binding | 215 | 2,5E-03 |
| kinase binding | 205 | 4,6E-12 |
