# Supplementary material for: miR-155 Contributes to the Immunoregulatory Function of Human Mesenchymal Stem Cells
Source: Front Immunol. 2021 Mar 26;12:624024. doi: 10.3389/fimmu.2021.624024 (PMC8033167; doi:10.3389/fimmu.2021.624024)
Supplement: Supplementary file 3 [file Table_3.docx]

**Supl. Table 3**: Gene Ontology enrichment analysis of cellular component for miR-155 target genes in pMSCs versus MSCs

| **Term** | **EnrichR Combined Score** | **Adjusted P-value** |
| --- | --- | --- |
| chromatin | 225 | 5,0E-11 |
| invadopodium | 215 | 6,5E-03 |
| micro-ribonucleoprotein complex | 189 | 3,0E-02 |
| platelet alpha granule lumen | 151 | 5,6E-05 |
| death-inducing signaling complex | 150 | 3,3E-02 |
| nuclear chromatin | 131 | 1,4E-07 |
| RISC-loading complex | 123 | 3,5E-02 |
| RISC complex | 123 | 3,5E-02 |
| RNAi effector complex | 123 | 3,5E-02 |
| nuclear chromosome part | 115 | 5,5E-08 |
| lateral element | 103 | 4,0E-02 |
| filopodium tip | 103 | 4,0E-02 |
| platelet alpha granule | 90 | 3,7E-04 |
| RNA polymerase II transcription factor complex | 89 | 6,0E-05 |
| axonal growth cone | 88 | 4,4E-02 |
| euchromatin | 84 | 2,3E-02 |
| endoplasmic reticulum lumen | 82 | 1,1E-05 |
| endoribonuclease complex | 67 | 5,4E-02 |
| Cul4A-RING E3 ubiquitin ligase complex | 67 | 5,4E-02 |
| nuclear transcription factor complex | 64 | 4,6E-03 |
