# Supplementary material for: miR-155 Contributes to the Immunoregulatory Function of Human Mesenchymal Stem Cells
Source: Front Immunol. 2021 Mar 26;12:624024. doi: 10.3389/fimmu.2021.624024 (PMC8033167; doi:10.3389/fimmu.2021.624024)
Supplement: Supplementary file 4 [file Table_4.docx]

**Supl. Table 4:** List of genes regulated by over-expression and down-regulation of indicated miRNAs

| \| **pre-miR-146a** \| \| \| \| --- \| --- \| --- \| \| **Gene Symbol** \| **mRna - Description** \| **Fold Change** \| \| PLSCR4 \| Homo sapiens phospholipid scramblase 4 (PLSCR4), transcript variant 1, mRNA. \| -2,62 \| \| HAS2 \| Homo sapiens hyaluronan synthase 2 (HAS2), mRNA. \| -2,27 \| \| SNRNP27 \| Homo sapiens small nuclear ribonucleoprotein 27kDa (U4/U6.U5) (SNRNP27), transcript variant 1, mRNA. \| -2,08 \| \| MIR31HG \| Homo sapiens MIR31 host gene (MIR31HG), long non-coding RNA. \| -2,05 \| \| TRBJ2-6 \| T cell receptor beta joining 2-6[gene_biotype:TR_gene transcript_biotype:TR_gene] \| -1,99 \| \| --- \| T cell receptor beta joining 2-6[gene_biotype:TR_gene transcript_biotype:TR_gene] \| -1,99 \| \| PHKB \| Homo sapiens phosphorylase kinase, beta (PHKB), transcript variant 1, mRNA. \| -1,97 \| \| CXCL6 \| Homo sapiens chemokine (C-X-C motif) ligand 6 (CXCL6), mRNA. \| -1,83 \| \| HLA-DRA \| Homo sapiens major histocompatibility complex, class II, DR alpha (HLA-DRA), mRNA. \| -1,81 \| \| --- \| RNA, 5S ribosomal pseudogene 356 [gene_biotype:rRNA transcript_biotype:rRNA] \| -1,80 \| \| CEMIP \| Homo sapiens cell migration inducing protein, hyaluronan binding (CEMIP), transcript variant 1, mRNA. \| -1,80 \| \| MPHOSPH6 \| Homo sapiens M-phase phosphoprotein 6 (MPHOSPH6), mRNA. \| -1,79 \| \| MAN2A1 \| Homo sapiens mannosidase, alpha, class 2A, member 1 (MAN2A1), mRNA. \| -1,78 \| \| SPRR2F \| Homo sapiens small proline-rich protein 2F (SPRR2F), mRNA. \| -1,77 \| \| WSB2 \| Homo sapiens WD repeat and SOCS box containing 2 (WSB2), transcript variant 1, mRNA. \| -1,76 \| \| CPA4 \| Homo sapiens carboxypeptidase A4 (CPA4), transcript variant 2, mRNA. \| -1,74 \| \| --- \| RNA, U6 small nuclear 1324, pseudogene [gene_biotype:snRNA transcript_biotype:snRNA] \| -1,73 \| \| IGHV3-48 \| Homo sapiens clone MCA1H myosin-reactive immunoglobulin heavy chain variable region mRNA, partial cds. \| -1,71 \| \| PTX3 \| Homo sapiens pentraxin 3, long (PTX3), mRNA. \| -1,70 \| \| --- \| Homo sapiens microRNA 4453 (MIR4453), microRNA. \| -1,70 \| \| VASN \| Homo sapiens vasorin (VASN), mRNA. \| -1,69 \| \| MTSS1 \| Homo sapiens metastasis suppressor 1 (MTSS1), transcript variant 1, mRNA. \| -1,67 \| \| TMX1 \| Homo sapiens thioredoxin-related transmembrane protein 1 (TMX1), mRNA. \| -1,66 \| \| --- \| Non-coding transcript identified by NONCODE: Linc \| -1,66 \| \| LIN7C \| Homo sapiens lin-7 homolog C (C. elegans) (LIN7C), mRNA. \| -1,66 \| \| LOC100101148 \| Homo sapiens FK506 binding protein 6, 36kDa pseudogene (LOC100101148), non-coding RNA. \| -1,65 \| \| DSPP \| Homo sapiens dentin sialophosphoprotein (DSPP), mRNA. \| -1,65 \| \| --- \| RNA, U6 small nuclear 781, pseudogene [gene_biotype:snRNA transcript_biotype:snRNA] \| -1,64 \| \| --- \| RNA, U7 small nuclear 47 pseudogene [gene_biotype:snRNA transcript_biotype:snRNA] \| -1,63 \| \| --- \| PREDICTED: Homo sapiens uncharacterized LOC101928451 (LOC101928451), transcript variant X1, misc_RNA. \| -1,61 \| \| CATSPER2P1 \| Homo sapiens cation channel, sperm associated 2 pseudogene 1 (CATSPER2P1), non-coding RNA. \| -1,59 \| \| RPSAP52 \| Homo sapiens ribosomal protein SA pseudogene 52 (RPSAP52), non-coding RNA. \| -1,59 \| \| MIR4710 \| Homo sapiens microRNA 4710 (MIR4710), microRNA. \| -1,58 \| \| GRPR \| Homo sapiens gastrin-releasing peptide receptor (GRPR), mRNA. \| -1,58 \| \| MIR3178 \| Homo sapiens microRNA 3178 (MIR3178), microRNA. \| -1,58 \| \| --- \| havana:known chromosome:GRCh38:1:121118126:121146826:1 gene:ENSG00000227193 gene_biotype:lincRNA transcript_biotype:lincRNA \| -1,56 \| \| SNAR-I \| Homo sapiens small ILF3/NF90-associated RNA I (SNAR-I), small nuclear RNA. \| -1,56 \| \| LOC100652871 \| PREDICTED: Homo sapiens glutathione S-transferase theta-4-like (LOC100652871), transcript variant X1, mRNA. \| -1,56 \| \| --- \| havana:known chromosome:GRCh38:2:130583549:130590463:-1 gene:ENSG00000232408 gene_biotype:antisense transcript_biotype:antisense \| -1,55 \| \| CMTM6 \| Homo sapiens CKLF-like MARVEL transmembrane domain containing 6 (CMTM6), mRNA. \| -1,55 \| \| --- \| ncrna:novel chromosome:GRCh38:15:90295041:90295123:1 gene:ENSG00000221309 gene_biotype:miRNA transcript_biotype:miRNA \| -1,55 \| \| --- \| RNA, U7 small nuclear 27 pseudogene [gene_biotype:snRNA transcript_biotype:snRNA] \| -1,54 \| \| PER3 \| Homo sapiens period circadian clock 3 (PER3), transcript variant 2, mRNA. \| -1,54 \| \| RAP2C \| Homo sapiens RAP2C, member of RAS oncogene family (RAP2C), transcript variant 1, mRNA. \| -1,54 \| \| --- \| RNA, 5S ribosomal pseudogene 111 [gene_biotype:rRNA transcript_biotype:rRNA] \| -1,54 \| \| LOC401357 \| Homo sapiens uncharacterized LOC401357 (LOC401357), long non-coding RNA. \| -1,53 \| \| MIR378I \| Homo sapiens microRNA 378i (MIR378I), microRNA. \| -1,53 \| \| --- \| mitochondrially encoded tRNA serine 1 (UCN) [gene_biotype:Mt_tRNA transcript_biotype:Mt_tRNA] \| -1,53 \| \| GALNT10 \| Homo sapiens polypeptide N-acetylgalactosaminyltransferase 10 (GALNT10), mRNA. \| -1,52 \| \| TERC \| Homo sapiens telomerase RNA component (TERC), telomerase RNA. \| -1,51 \| \| --- \| Non-coding transcript identified by NONCODE: Linc \| -1,51 \| \| --- \| RNA, 5S ribosomal pseudogene 463 [gene_biotype:rRNA transcript_biotype:rRNA] \| -1,51 \| \| --- \| Small nucleolar RNA U3 [gene_biotype:snoRNA transcript_biotype:snoRNA] \| -1,51 \| \| PP13 \| PREDICTED: Homo sapiens vegetative cell wall protein gp1 (PP13), misc_RNA. \| -1,51 \|  \| **pre-miR-155** \| \| \| \| --- \| --- \| --- \| \| **Gene Symbol** \| **mRna - Description** \| **Fold Change** \| \| --- \| Homo sapiens chloride channel, voltage-sensitive 3 (CLCN3), transcript variant a, mRNA. \| -4,68 \| \| H3F3AP4 \| Homo sapiens H3 histone, family 3A, pseudogene 4 (H3F3AP4), non-coding RNA. \| -4,33 \| \| SNORA22 \| Homo sapiens small nucleolar RNA, H/ACA box 22 (SNORA22), small nucleolar RNA. \| -4,09 \| \| --- \| mitochondrially encoded tRNA threonine [gene_biotype:Mt_tRNA transcript_biotype:Mt_tRNA] \| -3,80 \| \| IL6ST \| Homo sapiens interleukin 6 signal transducer (IL6ST), transcript variant 3, mRNA. \| -3,77 \| \| RPS2P46 \| ribosomal protein S2 pseudogene 46[gene_biotype:processed_pseudogene transcript_biotype:processed_pseudogene] \| -3,69 \| \| MIR1244-1 \| Homo sapiens microRNA 1244-1 (MIR1244-1), microRNA. \| -3,25 \| \| MIR1244-1 \| Homo sapiens microRNA 1244-1 (MIR1244-1), microRNA. \| -3,25 \| \| MIR1244-1 \| Homo sapiens microRNA 1244-1 (MIR1244-1), microRNA. \| -3,25 \| \| SCAMP1 \| Homo sapiens secretory carrier membrane protein 1 (SCAMP1), transcript variant 3, mRNA. \| -3,25 \| \| ENPP1 \| Homo sapiens ectonucleotide pyrophosphatase/phosphodiesterase 1 (ENPP1), mRNA. \| -3,22 \| \| ATP6V1C1 \| Homo sapiens ATPase, H+ transporting, lysosomal 42kDa, V1 subunit C1 (ATP6V1C1), mRNA. \| -3,08 \| \| MIR4461 \| Homo sapiens microRNA 4461 (MIR4461), microRNA. \| -3,07 \| \| TRAM1 \| Homo sapiens translocation associated membrane protein 1 (TRAM1), mRNA. \| -3,00 \| \| KITLG \| Homo sapiens KIT ligand (KITLG), transcript variant b, mRNA. \| -2,95 \| \| --- \| linc-KRTAP9-1 chr17:+:39264469-39271423 \| -2,94 \| \| HIST1H1B \| Homo sapiens histone cluster 1, H1b (HIST1H1B), mRNA. \| -2,92 \| \| RPL21P28 \| Homo sapiens ribosomal protein L21 pseudogene 28 (RPL21P28), non-coding RNA. \| -2,83 \| \| HIST1H2BM \| Homo sapiens histone cluster 1, H2bm (HIST1H2BM), mRNA. \| -2,82 \| \| HIST1H2AI \| Homo sapiens histone cluster 1, H2ai (HIST1H2AI), mRNA. \| -2,82 \| \| KLF2 \| Homo sapiens Kruppel-like factor 2 (KLF2), mRNA. \| -2,81 \| \| PTX3 \| Homo sapiens pentraxin 3, long (PTX3), mRNA. \| -2,77 \| \| --- \| mitochondrially encoded tRNA isoleucine [gene_biotype:Mt_tRNA transcript_biotype:Mt_tRNA] \| -2,76 \| \| INAFM2 \| Homo sapiens InaF-motif containing 2 (INAFM2), mRNA. \| -2,74 \| \| EPG5 \| Homo sapiens ectopic P-granules autophagy protein 5 homolog (C. elegans) (EPG5), mRNA. \| -2,73 \| \| --- \| mitochondrially encoded tRNA cysteine [gene_biotype:Mt_tRNA transcript_biotype:Mt_tRNA] \| -2,72 \| \| --- \| mitochondrially encoded tRNA methionine [gene_biotype:Mt_tRNA transcript_biotype:Mt_tRNA] \| -2,72 \| \| CEMIP \| Homo sapiens cell migration inducing protein, hyaluronan binding (CEMIP), transcript variant 1, mRNA. \| -2,69 \| \| TWF1 \| Homo sapiens twinfilin actin binding protein 1 (TWF1), transcript variant 1, mRNA. \| -2,67 \| \| --- \| Homo sapiens vascular endothelial zinc finger 1 (VEZF1), mRNA. \| -2,64 \| \| SEMA3A \| Homo sapiens sema domain, immunoglobulin domain (Ig), short basic domain, secreted, (semaphorin) 3A (SEMA3A), mRNA. \| -2,62 \| \| ABLIM1 \| PREDICTED: Homo sapiens actin binding LIM protein 1 (ABLIM1), transcript variant X8, mRNA. \| -2,59 \| \| --- \| Homo sapiens B-cell receptor-associated protein 31 (BCAP31), transcript variant 3, mRNA. \| -2,57 \| \| PBK \| Homo sapiens PDZ binding kinase (PBK), transcript variant 2, mRNA. \| -2,56 \| \| ARL2BP \| Homo sapiens ADP-ribosylation factor-like 2 binding protein (ARL2BP), mRNA. \| -2,56 \| \| TMX3 \| Homo sapiens thioredoxin-related transmembrane protein 3 (TMX3), mRNA. \| -2,55 \| \| ANKRD28 \| Homo sapiens ankyrin repeat domain 28 (ANKRD28), transcript variant 2, mRNA. \| -2,54 \| \| EBF2 \| Homo sapiens early B-cell factor 2 (EBF2), mRNA. \| -2,53 \| \| --- \| Non-coding transcript identified by NONCODE \| -2,52 \| \| HIST2H2BA \| Homo sapiens histone cluster 2, H2ba (pseudogene) (HIST2H2BA), non-coding RNA. \| -2,51 \| \| --- \| ensembl:known chromosome:GRCh38:5:60430738:60431112:1 gene:ENSG00000280447 gene_biotype:protein_coding transcript_biotype:protein_coding \| -2,51 \| \| TBC1D2 \| Homo sapiens TBC1 domain family, member 2 (TBC1D2), transcript variant 1, mRNA. \| -2,51 \| \| HLTF \| Homo sapiens helicase-like transcription factor (HLTF), transcript variant 1, mRNA. \| -2,49 \| \| CCNA2 \| Homo sapiens cyclin A2 (CCNA2), mRNA. \| -2,48 \| \| DSEL \| Homo sapiens dermatan sulfate epimerase-like (DSEL), mRNA. \| -2,46 \| \| FAR1 \| Homo sapiens fatty acyl CoA reductase 1 (FAR1), mRNA. \| -2,45 \| \| TCAF1 \| Homo sapiens TRPM8 channel-associated factor 1 (TCAF1), transcript variant 2, mRNA. \| -2,45 \| \| CLCN3 \| Homo sapiens chloride channel, voltage-sensitive 3 (CLCN3), transcript variant a, mRNA. \| -2,44 \| \| MIR31HG \| Homo sapiens MIR31 host gene (MIR31HG), long non-coding RNA. \| -2,44 \| \| FLG \| Homo sapiens filaggrin (FLG), mRNA. \| -2,44 \| \| NBPF19 \| PREDICTED: Homo sapiens neuroblastoma breakpoint family, member 19 (NBPF19), transcript variant X1, mRNA. \| -2,44 \| \| --- \| linc-TSC1-1 chr9:-:135894815-135896553 \| -2,43 \| \| --- \| mitochondrially encoded tRNA cysteine [gene_biotype:Mt_tRNA transcript_biotype:Mt_tRNA] \| -2,42 \| \| PCDH18 \| Homo sapiens protocadherin 18 (PCDH18), transcript variant 2, mRNA. \| -2,40 \| \| --- \| havana:known chromosome:GRCh38:11:32097143:32105091:-1 gene:ENSG00000255252 gene_biotype:antisense transcript_biotype:antisense \| -2,40 \| \| KRTAP2-3 \| Homo sapiens keratin associated protein 2-3 (KRTAP2-3), mRNA. \| -2,39 \| \| HIST2H2AB \| Homo sapiens histone cluster 2, H2ab (HIST2H2AB), mRNA. \| -2,38 \| \| UBQLN1 \| Homo sapiens ubiquilin 1 (UBQLN1), transcript variant 1, mRNA. \| -2,37 \| \| SUZ12 \| Homo sapiens SUZ12 polycomb repressive complex 2 subunit (SUZ12), mRNA. \| -2,36 \| \| --- \| Homo sapiens small nucleolar RNA, C/D box 50A (SNORD50A), small nucleolar RNA. \| -2,36 \| \| SCARNA9 \| Homo sapiens small Cajal body-specific RNA 9 (SCARNA9), guide RNA. \| -2,35 \| \| TYMS \| Homo sapiens thymidylate synthetase (TYMS), mRNA. \| -2,34 \| \| FGF7 \| Homo sapiens fibroblast growth factor 7 (FGF7), mRNA. \| -2,34 \| \| SLC44A2 \| Homo sapiens solute carrier family 44 (choline transporter), member 2 (SLC44A2), transcript variant 2, mRNA. \| -2,33 \| \| SKIV2L2 \| Homo sapiens superkiller viralicidic activity 2-like 2 (S. cerevisiae) (SKIV2L2), mRNA. \| -2,32 \| \| --- \| Homo sapiens small nucleolar RNA, C/D box 45A (SNORD45A), small nucleolar RNA. \| -2,30 \| \| CBFB \| Homo sapiens core-binding factor, beta subunit (CBFB), transcript variant 2, mRNA. \| -2,30 \| \| VAMP3 \| Homo sapiens vesicle-associated membrane protein 3 (VAMP3), mRNA. \| -2,30 \| \| --- \| Homo sapiens sorting nexin 7 (SNX7), transcript variant 1, mRNA. \| -2,29 \| \| MIR221 \| Homo sapiens microRNA 221 (MIR221), microRNA. \| -2,28 \| \| LIN7C \| Homo sapiens lin-7 homolog C (C. elegans) (LIN7C), mRNA. \| -2,27 \| \| RAPH1 \| Homo sapiens Ras association (RalGDS/AF-6) and pleckstrin homology domains 1 (RAPH1), transcript variant 3, mRNA. \| -2,27 \| \| TULP4 \| PREDICTED: Homo sapiens tubby like protein 4 (TULP4), transcript variant X1, mRNA. \| -2,26 \| \| CSNK1G2 \| Homo sapiens casein kinase 1, gamma 2 (CSNK1G2), mRNA. \| -2,26 \| \| PIGY \| Homo sapiens phosphatidylinositol glycan anchor biosynthesis, class Y (PIGY), mRNA. \| -2,26 \| \| --- \| Homo sapiens transcription elongation factor B (SIII), polypeptide 1 (15kDa, elongin C) (TCEB1), transcript variant 2, mRNA. \| -2,26 \| \| DUSP14 \| Homo sapiens dual specificity phosphatase 14 (DUSP14), mRNA. \| -2,26 \| \| MCM8 \| Homo sapiens minichromosome maintenance 8 homologous recombination repair factor (MCM8), transcript variant 3, mRNA. \| -2,25 \| \| CPD \| Homo sapiens carboxypeptidase D (CPD), transcript variant 2, mRNA. \| -2,25 \| \| PKN2 \| Homo sapiens protein kinase N2 (PKN2), mRNA. \| -2,25 \| \| CFL2 \| Homo sapiens cofilin 2 (muscle) (CFL2), transcript variant 5, mRNA. \| -2,24 \| \| RPS6KA3 \| Homo sapiens ribosomal protein S6 kinase, 90kDa, polypeptide 3 (RPS6KA3), mRNA. \| -2,24 \| \| ARHGAP11B \| Homo sapiens Rho GTPase activating protein 11B (ARHGAP11B), mRNA. \| -2,23 \| \| ERBB2IP \| Homo sapiens erbb2 interacting protein (ERBB2IP), transcript variant 7, mRNA. \| -2,23 \| \| ANKRD1 \| Homo sapiens ankyrin repeat domain 1 (cardiac muscle) (ANKRD1), mRNA. \| -2,23 \| \| MYBL1 \| Homo sapiens v-myb avian myeloblastosis viral oncogene homolog-like 1 (MYBL1), transcript variant 1, mRNA. \| -2,22 \| \| KCTD3 \| Homo sapiens potassium channel tetramerization domain containing 3 (KCTD3), mRNA. \| -2,21 \| \| NABP1 \| Homo sapiens nucleic acid binding protein 1 (NABP1), transcript variant 1, mRNA. \| -2,21 \| \| RAB11FIP2 \| Homo sapiens RAB11 family interacting protein 2 (class I) (RAB11FIP2), mRNA. \| -2,21 \| \| MYO1E \| Homo sapiens myosin IE (MYO1E), mRNA. \| -2,21 \| \| SNORD59A \| Homo sapiens small nucleolar RNA, C/D box 59A (SNORD59A), small nucleolar RNA. \| -2,20 \| \| TPGS2 \| Homo sapiens tubulin polyglutamylase complex subunit 2 (TPGS2), transcript variant 2, mRNA. \| -2,20 \| \| HLA-DRA \| Homo sapiens major histocompatibility complex, class II, DR alpha (HLA-DRA), mRNA. \| -2,20 \| \| --- \| Homo sapiens fibroblast growth factor 7 (FGF7), mRNA. \| -2,20 \| \| --- \| Homo sapiens long intergenic non-protein coding RNA 1000 (LINC01000), long non-coding RNA. \| -2,20 \| \| LOC101060341 \| PREDICTED: Homo sapiens putative uncharacterized protein FLJ46235 (LOC101060341), mRNA. \| -2,20 \| \| ADAMTS5 \| Homo sapiens ADAM metallopeptidase with thrombospondin type 1 motif, 5 (ADAMTS5), mRNA. \| -2,20 \| \| TAOK1 \| Homo sapiens TAO kinase 1 (TAOK1), transcript variant 1, mRNA. \| -2,19 \| \| --- \| Small nucleolar RNA SNORA22 [gene_biotype:snoRNA transcript_biotype:snoRNA] \| -2,19 \| \| ANLN \| Homo sapiens anillin actin binding protein (ANLN), transcript variant 2, mRNA. \| -2,18 \| \| LOC105370447 \| PREDICTED: Homo sapiens uncharacterized LOC105370447 (LOC105370447), ncRNA. \| -2,18 \| \| JUN \| Homo sapiens jun proto-oncogene (JUN), mRNA. \| -2,18 \| \| SMU1 \| Homo sapiens smu-1 suppressor of mec-8 and unc-52 homolog (C. elegans) (SMU1), mRNA. \| -2,18 \| \| CDC73 \| Homo sapiens cell division cycle 73 (CDC73), mRNA. \| -2,17 \| \| KIF20B \| Homo sapiens kinesin family member 20B (KIF20B), transcript variant 1, mRNA. \| -2,16 \| \| WDFY1 \| Homo sapiens WD repeat and FYVE domain containing 1 (WDFY1), mRNA. \| -2,16 \| \| --- \| Homo sapiens small nucleolar RNA, C/D box 14C (SNORD14C), small nucleolar RNA. \| -2,15 \| \| RALGPS2 \| Homo sapiens Ral GEF with PH domain and SH3 binding motif 2 (RALGPS2), transcript variant 2, mRNA. \| -2,14 \| \| --- \| mitochondrially encoded tRNA aspartic acid [gene_biotype:Mt_tRNA transcript_biotype:Mt_tRNA] \| -2,13 \| \| LINC-PINT \| Homo sapiens long intergenic non-protein coding RNA, p53 induced transcript (LINC-PINT), transcript variant 9, long non-coding RNA. \| -2,13 \| \| PICALM \| Homo sapiens phosphatidylinositol binding clathrin assembly protein (PICALM), transcript variant 2, mRNA. \| -2,13 \| \| TAF7 \| Homo sapiens TAF7 RNA polymerase II, TATA box binding protein (TBP)-associated factor, 55kDa (TAF7), mRNA. \| -2,13 \| \| UGCG \| Homo sapiens UDP-glucose ceramide glucosyltransferase (UGCG), mRNA. \| -2,13 \| \| ABI2 \| Homo sapiens abl-interactor 2 (ABI2), transcript variant 1, mRNA. \| -2,13 \| \| CITED2 \| Homo sapiens Cbp/p300-interacting transactivator, with Glu/Asp-rich carboxy-terminal domain, 2 (CITED2), transcript variant 2, mRNA. \| -2,12 \| \| OSMR \| Homo sapiens oncostatin M receptor (OSMR), transcript variant 2, mRNA. \| -2,11 \| \| --- \| Homo sapiens small nucleolar RNA, C/D box 47 (SNORD47), small nucleolar RNA. \| -2,11 \| \| TOP2A \| Homo sapiens topoisomerase (DNA) II alpha 170kDa (TOP2A), mRNA. \| -2,11 \| \| SQRDL \| Homo sapiens sulfide quinone reductase-like (yeast) (SQRDL), transcript variant 2, mRNA. \| -2,11 \| \| NUSAP1 \| Homo sapiens nucleolar and spindle associated protein 1 (NUSAP1), transcript variant 4, mRNA. \| -2,11 \| \| SLK \| Homo sapiens STE20-like kinase (SLK), transcript variant 2, mRNA. \| -2,09 \| \| CARHSP1 \| Homo sapiens calcium regulated heat stable protein 1, 24kDa (CARHSP1), transcript variant 2, mRNA. \| -2,09 \| \| C16orf72 \| PREDICTED: Homo sapiens chromosome 16 open reading frame 72 (C16orf72), transcript variant X1, mRNA. \| -2,09 \| \| ZFP36 \| Homo sapiens ZFP36 ring finger protein (ZFP36), mRNA. \| -2,09 \| \| BUB1 \| Homo sapiens BUB1 mitotic checkpoint serine/threonine kinase (BUB1), transcript variant 2, mRNA. \| -2,08 \| \| --- \| PREDICTED: Homo sapiens neurofibromin 1 (NF1), transcript variant X8, mRNA. \| -2,08 \| \| --- \| Non-coding transcript identified by NONCODE: Linc \| -2,07 \| \| PDE5A \| Homo sapiens phosphodiesterase 5A, cGMP-specific (PDE5A), transcript variant 1, mRNA. \| -2,07 \| \| --- \| mitochondrially encoded tRNA leucine 2 (CUN) [gene_biotype:Mt_tRNA transcript_biotype:Mt_tRNA] \| -2,07 \| \| MARVELD1 \| Homo sapiens MARVEL domain containing 1 (MARVELD1), mRNA. \| -2,06 \| \| VEZF1 \| Homo sapiens vascular endothelial zinc finger 1 (VEZF1), mRNA. \| -2,06 \| \| DTL \| Homo sapiens denticleless E3 ubiquitin protein ligase homolog (Drosophila) (DTL), transcript variant 2, mRNA. \| -2,05 \| \| --- \| Homo sapiens mRNA; cDNA DKFZp686O1684 (from clone DKFZp686O1684). \| -2,05 \| \| TAF1D \| TATA box binding protein (TBP)-associated factor, RNA polymerase I, D, 41kDa [gene_biotype:protein_coding transcript_biotype:nonsense_mediated_decay] \| -2,05 \| \| PLEKHB2 \| Homo sapiens pleckstrin homology domain containing, family B (evectins) member 2 (PLEKHB2), transcript variant 3, mRNA. \| -2,05 \| \| SNORD60 \| Homo sapiens small nucleolar RNA, C/D box 60 (SNORD60), small nucleolar RNA. \| -2,04 \| \| ACTR10 \| Homo sapiens actin-related protein 10 homolog (S. cerevisiae) (ACTR10), mRNA. \| -2,04 \| \| F2RL2 \| Homo sapiens coagulation factor II (thrombin) receptor-like 2 (F2RL2), transcript variant 2, mRNA. \| -2,04 \| \| EZR \| Homo sapiens ezrin (EZR), transcript variant 2, mRNA. \| -2,03 \| \| ZAK \| Homo sapiens sterile alpha motif and leucine zipper containing kinase AZK (ZAK), transcript variant 1, mRNA. \| -2,03 \| \| HIVEP2 \| Homo sapiens human immunodeficiency virus type I enhancer binding protein 2 (HIVEP2), mRNA. \| -2,03 \| \| FAM126A \| Homo sapiens family with sequence similarity 126, member A (FAM126A), mRNA. \| -2,03 \| \| ANKRD36B \| Homo sapiens ankyrin repeat domain 36B (ANKRD36B), mRNA. \| -2,03 \| \| --- \| Homo sapiens spermine synthase (SMS), transcript variant 2, mRNA. \| -2,03 \| \| FAM21C \| Homo sapiens family with sequence similarity 21, member C, mRNA (cDNA clone IMAGE:5123514). \| -2,02 \| \| STK38 \| Homo sapiens serine/threonine kinase 38 (STK38), transcript variant 2, mRNA. \| -2,02 \| \| --- \| Homo sapiens small nucleolar RNA, C/D box 26 (SNORD26), small nucleolar RNA. \| -2,02 \| \| F2R \| Homo sapiens coagulation factor II (thrombin) receptor (F2R), transcript variant 2, mRNA. \| -2,02 \| \| VPS4B \| Homo sapiens vacuolar protein sorting 4 homolog B (S. cerevisiae) (VPS4B), mRNA. \| -2,01 \| \| MKI67 \| Homo sapiens marker of proliferation Ki-67 (MKI67), transcript variant 2, mRNA. \| -2,00 \| \| RAB23 \| Homo sapiens RAB23, member RAS oncogene family (RAB23), transcript variant 3, mRNA. \| -2,00 \| \| --- \| Homo sapiens eukaryotic translation initiation factor 3, subunit J (EIF3J), transcript variant 2, mRNA. \| -2,00 \| \| PAK2 \| Homo sapiens p21 protein (Cdc42/Rac)-activated kinase 2 (PAK2), mRNA. \| -2,00 \| \| LIFR \| Homo sapiens leukemia inhibitory factor receptor alpha (LIFR), transcript variant 1, mRNA. \| -1,99 \| \| MYO10 \| Homo sapiens myosin X (MYO10), mRNA. \| -1,99 \| \| ANKFY1 \| Homo sapiens ankyrin repeat and FYVE domain containing 1 (ANKFY1), transcript variant 3, mRNA. \| -1,99 \| \| RUFY2 \| Homo sapiens RUN and FYVE domain containing 2 (RUFY2), transcript variant 2, mRNA. \| -1,99 \| \| ESF1 \| Homo sapiens ESF1 nucleolar pre-rRNA processing protein homolog (ESF1), transcript variant 2, mRNA. \| -1,99 \| \| PRKCI \| Homo sapiens protein kinase C, iota (PRKCI), mRNA. \| -1,98 \| \| ZSWIM6 \| Homo sapiens zinc finger, SWIM-type containing 6 (ZSWIM6), mRNA. \| -1,98 \| \| PTPRG \| Homo sapiens protein tyrosine phosphatase, receptor type, G (PTPRG), mRNA. \| -1,98 \| \| TXNDC12 \| Homo sapiens thioredoxin domain containing 12 (endoplasmic reticulum) (TXNDC12), transcript variant 1, mRNA. \| -1,98 \| \| --- \| Homo sapiens B-cell receptor-associated protein 31 (BCAP31), transcript variant 3, mRNA. \| -1,98 \| \| FJX1 \| Homo sapiens four jointed box 1 (FJX1), mRNA. \| -1,97 \| \| PRR11 \| Homo sapiens proline rich 11 (PRR11), mRNA. \| -1,97 \| \| TBCK \| Homo sapiens TBC1 domain containing kinase (TBCK), transcript variant 1, mRNA. \| -1,97 \| \| SLC43A3 \| Homo sapiens solute carrier family 43, member 3 (SLC43A3), transcript variant 2, mRNA. \| -1,97 \| \| NCAPG \| Homo sapiens non-SMC condensin I complex, subunit G (NCAPG), transcript variant 1, mRNA. \| -1,97 \| \| --- \| Homo sapiens MIR4435-2 host gene (MIR4435-2HG), transcript variant 2, long non-coding RNA. \| -1,96 \| \| NTAN1 \| Homo sapiens N-terminal asparagine amidase (NTAN1), transcript variant 2, mRNA. \| -1,96 \| \| FMNL2 \| Homo sapiens formin-like 2 (FMNL2), mRNA. \| -1,96 \| \| KIF20A \| Homo sapiens kinesin family member 20A (KIF20A), mRNA. \| -1,96 \| \| ZFP36L2 \| Homo sapiens ZFP36 ring finger protein-like 2 (ZFP36L2), mRNA. \| -1,95 \| \| CTSO \| Homo sapiens cathepsin O (CTSO), mRNA. \| -1,95 \| \| --- \| Homo sapiens MIR4435-2 host gene (MIR4435-2HG), transcript variant 2, long non-coding RNA. \| -1,94 \| \| --- \| Homo sapiens DBF4 zinc finger (DBF4), mRNA. \| -1,94 \| \| LOC729218 \| Homo sapiens uncharacterized LOC729218 (LOC729218), transcript variant 2, non-coding RNA. \| -1,94 \| \| LOC101930131 \| PREDICTED: Homo sapiens uncharacterized LOC101930131 (LOC101930131), transcript variant X3, ncRNA. \| -1,94 \| \| --- \| cdna:genscan chromosome:GRCh38:9:111014080:111153073:1 transcript_biotype:protein_coding \| -1,94 \| \| DIAPH3 \| Homo sapiens diaphanous-related formin 3 (DIAPH3), transcript variant 1, mRNA. \| -1,94 \| \| B3GALT2 \| Homo sapiens UDP-Gal:betaGlcNAc beta 1,3-galactosyltransferase, polypeptide 2 (B3GALT2), mRNA. \| -1,94 \| \| LOC220729 \| Homo sapiens succinate dehydrogenase complex, subunit A, flavoprotein (Fp) pseudogene (LOC220729), non-coding RNA. \| -1,93 \| \| SGTB \| Homo sapiens small glutamine-rich tetratricopeptide repeat (TPR)-containing, beta (SGTB), mRNA. \| -1,93 \| \| --- \| havana:known chromosome:GRCh38:15:39593580:39594231:-1 gene:ENSG00000259279 gene_biotype:antisense transcript_biotype:antisense \| -1,93 \| \| --- \| mitochondrially encoded tRNA tyrosine [gene_biotype:Mt_tRNA transcript_biotype:Mt_tRNA] \| -1,93 \| \| C15orf41 \| Homo sapiens chromosome 15 open reading frame 41 (C15orf41), transcript variant 1, mRNA. \| -1,93 \| \| ELL2 \| Homo sapiens elongation factor, RNA polymerase II, 2 (ELL2), mRNA. \| -1,93 \| \| MDM2 \| Homo sapiens MDM2 proto-oncogene, E3 ubiquitin protein ligase (MDM2), transcript variant 3, mRNA. \| -1,92 \| \| --- \| Non-coding transcript identified by NONCODE \| -1,92 \| \| PRR16 \| Homo sapiens proline rich 16 (PRR16), transcript variant 1, mRNA. \| -1,92 \| \| STRN3 \| Homo sapiens striatin, calmodulin binding protein 3 (STRN3), transcript variant 1, mRNA. \| -1,92 \| \| LYSMD3 \| Homo sapiens LysM, putative peptidoglycan-binding, domain containing 3 (LYSMD3), transcript variant 2, mRNA. \| -1,92 \| \| CBWD2 \| Homo sapiens COBW domain containing 2 (CBWD2), mRNA. \| -1,92 \| \| --- \| RNA, U5A small nuclear 8, pseudogene [gene_biotype:snRNA transcript_biotype:snRNA] \| -1,91 \| \| SNORD63 \| Homo sapiens small nucleolar RNA, C/D box 63 (SNORD63), small nucleolar RNA. \| -1,91 \| \| --- \| ncrna:novel scaffold:GRCh38:GL000224.1:166714:166794:1 gene:ENSG00000275189 gene_biotype:miRNA transcript_biotype:miRNA \| -1,91 \| \| RAB27B \| Homo sapiens RAB27B, member RAS oncogene family (RAB27B), mRNA. \| -1,91 \| \| MTSS1 \| Homo sapiens metastasis suppressor 1 (MTSS1), transcript variant 1, mRNA. \| -1,91 \| \| PTGIS \| Homo sapiens prostaglandin I2 (prostacyclin) synthase (PTGIS), mRNA. \| -1,91 \| \| GAS6-AS2 \| Homo sapiens GAS6 antisense RNA 2 (head to head) (GAS6-AS2), long non-coding RNA. \| -1,91 \| \| VGLL3 \| Homo sapiens vestigial-like family member 3 (VGLL3), mRNA. \| -1,91 \| \| NUP153 \| Homo sapiens nucleoporin 153kDa (NUP153), transcript variant 1, mRNA. \| -1,91 \| \| AXL \| Homo sapiens AXL receptor tyrosine kinase (AXL), transcript variant 3, mRNA. \| -1,91 \| \| CTSK \| Homo sapiens cathepsin K (CTSK), mRNA. \| -1,91 \| \| RPL13AP20 \| Homo sapiens ribosomal protein L13a pseudogene 20 (RPL13AP20), non-coding RNA. \| -1,91 \| \| ANTXR2 \| Homo sapiens anthrax toxin receptor 2 (ANTXR2), transcript variant 2, mRNA. \| -1,90 \| \| SAMHD1 \| Homo sapiens SAM domain and HD domain 1 (SAMHD1), mRNA. \| -1,90 \| \| --- \| Homo sapiens sorting nexin 29 (SNX29), mRNA. \| -1,89 \| \| DPP4 \| Homo sapiens dipeptidyl-peptidase 4 (DPP4), mRNA. \| -1,89 \| \| PRKD1 \| Homo sapiens protein kinase D1 (PRKD1), mRNA. \| -1,89 \| \| CD109 \| Homo sapiens CD109 molecule (CD109), transcript variant 2, mRNA. \| -1,89 \| \| KRCC1 \| Homo sapiens lysine-rich coiled-coil 1 (KRCC1), transcript variant 2, mRNA. \| -1,89 \| \| USP1 \| Homo sapiens ubiquitin specific peptidase 1 (USP1), transcript variant 2, mRNA. \| -1,89 \| \| PEBP1 \| Homo sapiens phosphatidylethanolamine binding protein 1 (PEBP1), mRNA. \| -1,88 \| \| GANAB \| Homo sapiens glucosidase, alpha; neutral AB (GANAB), transcript variant 4, mRNA. \| -1,88 \| \| ATP8B1 \| Homo sapiens ATPase, aminophospholipid transporter, class I, type 8B, member 1 (ATP8B1), mRNA. \| -1,88 \| \| STYX \| Homo sapiens serine/threonine/tyrosine interacting protein (STYX), transcript variant 2, mRNA. \| -1,88 \| \| UAP1 \| Homo sapiens UDP-N-acetylglucosamine pyrophosphorylase 1 (UAP1), mRNA. \| -1,88 \| \| AFF4 \| Homo sapiens AF4/FMR2 family, member 4 (AFF4), mRNA. \| -1,88 \| \| --- \| Non-coding transcript identified by NONCODE \| -1,87 \| \| SPRED1 \| Homo sapiens sprouty-related, EVH1 domain containing 1 (SPRED1), mRNA. \| -1,87 \| \| PRKDC \| Homo sapiens protein kinase, DNA-activated, catalytic polypeptide (PRKDC), transcript variant 2, mRNA. \| -1,87 \| \| LACC1 \| Homo sapiens laccase (multicopper oxidoreductase) domain containing 1 (LACC1), transcript variant 1, mRNA. \| -1,87 \| \| --- \| Homo sapiens cDNA clone IMAGE:3856788. \| -1,87 \| \| LSM14A \| Homo sapiens LSM14A mRNA processing body assembly factor (LSM14A), transcript variant 1, mRNA. \| -1,87 \| \| PLA2G12A \| Homo sapiens phospholipase A2, group XIIA (PLA2G12A), mRNA. \| -1,87 \| \| --- \| mitochondrially encoded tRNA lysine [gene_biotype:Mt_tRNA transcript_biotype:Mt_tRNA] \| -1,86 \| \| RNF26 \| Homo sapiens ring finger protein 26 (RNF26), mRNA. \| -1,86 \| \| CAMK2D \| Homo sapiens calcium/calmodulin-dependent protein kinase II delta (CAMK2D), transcript variant 3, mRNA. \| -1,86 \| \| MEOX2 \| Homo sapiens mesenchyme homeobox 2 (MEOX2), mRNA. \| -1,86 \| \| PI4KAP1 \| Homo sapiens phosphatidylinositol 4-kinase, catalytic, alpha pseudogene 1 (PI4KAP1), non-coding RNA. \| -1,86 \| \| KIAA1715 \| Homo sapiens KIAA1715 (KIAA1715), transcript variant 1, mRNA. \| -1,86 \| \| --- \| Non-coding transcript identified by NONCODE: Antisense \| -1,86 \| \| KIAA0368 \| Homo sapiens KIAA0368 (KIAA0368), mRNA. \| -1,86 \| \| ARHGAP21 \| Homo sapiens Rho GTPase activating protein 21 (ARHGAP21), mRNA. \| -1,86 \| \| STAT1 \| Homo sapiens signal transducer and activator of transcription 1, 91kDa (STAT1), transcript variant alpha, mRNA. \| -1,86 \| \| TOX \| Homo sapiens thymocyte selection-associated high mobility group box (TOX), mRNA. \| -1,85 \| \| UBE2R2 \| Homo sapiens ubiquitin-conjugating enzyme E2R 2 (UBE2R2), mRNA. \| -1,85 \| \| TGFBR2 \| Homo sapiens transforming growth factor, beta receptor II (70/80kDa) (TGFBR2), transcript variant 1, mRNA. \| -1,85 \| \| WDR45B \| Homo sapiens WD repeat domain 45B (WDR45B), mRNA. \| -1,84 \| \| SNRPN \| Homo sapiens clone kid4 SNURF-SNRPN mRNA, downstream untranslated exons, alternatively spliced. \| -1,84 \| \| TPRG1L \| Homo sapiens tumor protein p63 regulated 1-like (TPRG1L), mRNA. \| -1,84 \| \| TRAPPC8 \| Homo sapiens trafficking protein particle complex 8 (TRAPPC8), mRNA. \| -1,84 \| \| GOLM1 \| Homo sapiens golgi membrane protein 1 (GOLM1), transcript variant 1, mRNA. \| -1,84 \| \| DEK \| Homo sapiens DEK proto-oncogene (DEK), transcript variant 2, mRNA. \| -1,84 \| \| MRPL45 \| Homo sapiens mitochondrial ribosomal protein L45 (MRPL45), transcript variant 2, mRNA. \| -1,84 \| \| SESTD1 \| Homo sapiens SEC14 and spectrin domains 1 (SESTD1), mRNA. \| -1,84 \| \| ID1 \| Homo sapiens inhibitor of DNA binding 1, dominant negative helix-loop-helix protein (ID1), transcript variant 1, mRNA. \| -1,84 \| \| PBRM1 \| Homo sapiens polybromo 1 (PBRM1), mRNA. \| -1,84 \| \| --- \| Homo sapiens small nucleolar RNA, C/D box 34 (SNORD34), small nucleolar RNA. \| -1,84 \| \| FOPNL \| Homo sapiens FGFR1OP N-terminal like (FOPNL), transcript variant 2, mRNA. \| -1,83 \| \| ARSJ \| Homo sapiens arylsulfatase family, member J (ARSJ), mRNA. \| -1,83 \| \| KIF11 \| Homo sapiens kinesin family member 11 (KIF11), mRNA. \| -1,83 \| \| SPDL1 \| Homo sapiens spindle apparatus coiled-coil protein 1 (SPDL1), mRNA. \| -1,83 \| \| METTL9 \| Homo sapiens methyltransferase like 9 (METTL9), transcript variant 2, mRNA. \| -1,83 \| \| CDV3 \| Homo sapiens CDV3 homolog (mouse) (CDV3), transcript variant 1, mRNA. \| -1,83 \| \| TPX2 \| Homo sapiens TPX2, microtubule-associated (TPX2), mRNA. \| -1,83 \| \| CEP170P1 \| Homo sapiens centrosomal protein 170kDa pseudogene 1 (CEP170P1), non-coding RNA. \| -1,83 \| \| --- \| Non-coding transcript identified by NONCODE: Linc \| -1,83 \| \| ZMYND11 \| Homo sapiens zinc finger, MYND-type containing 11 (ZMYND11), transcript variant 4, mRNA. \| -1,83 \| \| LOC101928451 \| PREDICTED: Homo sapiens uncharacterized LOC101928451 (LOC101928451), transcript variant X2, misc_RNA. \| -1,83 \| \| STK17A \| Homo sapiens serine/threonine kinase 17a (STK17A), mRNA. \| -1,83 \| \| SNORD114-3 \| Homo sapiens small nucleolar RNA, C/D box 114-3 (SNORD114-3), small nucleolar RNA. \| -1,83 \| \| TWSG1 \| Homo sapiens twisted gastrulation BMP signaling modulator 1 (TWSG1), mRNA. \| -1,82 \| \| SLC12A6 \| Homo sapiens solute carrier family 12 (potassium/chloride transporter), member 6 (SLC12A6), transcript variant 3, mRNA. \| -1,82 \| \| MAN2A1 \| Homo sapiens mannosidase, alpha, class 2A, member 1 (MAN2A1), mRNA. \| -1,82 \| \| --- \| Homo sapiens ribosomal protein L31 (RPL31), transcript variant 1, mRNA. \| -1,82 \| \| ADM \| Homo sapiens adrenomedullin (ADM), mRNA. \| -1,82 \| \| DNAPTP3 \| Homo sapiens cDNA FLJ36958 fis, clone BRACE2005881. \| -1,82 \| \| CSE1L \| Homo sapiens CSE1 chromosome segregation 1-like (yeast) (CSE1L), transcript variant 2, mRNA. \| -1,82 \| \| --- \| Non-coding transcript identified by NONCODE: Linc \| -1,82 \| \| IFNAR1 \| Homo sapiens interferon (alpha, beta and omega) receptor 1 (IFNAR1), mRNA. \| -1,82 \| \| TTC37 \| Homo sapiens tetratricopeptide repeat domain 37 (TTC37), mRNA. \| -1,81 \| \| ECT2 \| Homo sapiens epithelial cell transforming 2 (ECT2), transcript variant 1, mRNA. \| -1,81 \| \| MIR21 \| Homo sapiens microRNA 21 (MIR21), microRNA. \| -1,81 \| \| ARIH1 \| Homo sapiens ariadne RBR E3 ubiquitin protein ligase 1 (ARIH1), mRNA. \| -1,81 \| \| METRNL \| Homo sapiens meteorin, glial cell differentiation regulator-like (METRNL), mRNA. \| -1,81 \| \| MOCS2 \| Homo sapiens molybdenum cofactor synthesis 2 (MOCS2), transcript variant 3, mRNA. \| -1,81 \| \| CDC20 \| Homo sapiens cell division cycle 20 (CDC20), mRNA. \| -1,81 \| \| --- \| Homo sapiens high mobility group box 1 (HMGB1), transcript variant 3, mRNA. \| -1,81 \| \| GCNT1 \| Homo sapiens glucosaminyl (N-acetyl) transferase 1, core 2 (GCNT1), transcript variant 3, mRNA. \| -1,81 \| \| TNFRSF11B \| Homo sapiens tumor necrosis factor receptor superfamily, member 11b (TNFRSF11B), mRNA. \| -1,81 \| \| UTRN \| Homo sapiens utrophin (UTRN), mRNA. \| -1,81 \| \| UFL1 \| Homo sapiens UFM1-specific ligase 1 (UFL1), mRNA. \| -1,80 \| \| RCN2 \| Homo sapiens reticulocalbin 2, EF-hand calcium binding domain (RCN2), transcript variant 2, mRNA. \| -1,80 \| \| CASP3 \| Homo sapiens caspase 3, apoptosis-related cysteine peptidase (CASP3), transcript variant alpha, mRNA. \| -1,80 \| \| MTMR10 \| Homo sapiens myotubularin related protein 10 (MTMR10), mRNA. \| -1,80 \| \| CHI3L1 \| Homo sapiens chitinase 3-like 1 (cartilage glycoprotein-39) (CHI3L1), mRNA. \| -1,80 \| \| SMURF2 \| Homo sapiens SMAD specific E3 ubiquitin protein ligase 2 (SMURF2), mRNA. \| -1,80 \| \| SPIN1 \| Homo sapiens spindlin 1 (SPIN1), mRNA. \| -1,80 \| \| KDELC2 \| Homo sapiens KDEL (Lys-Asp-Glu-Leu) containing 2 (KDELC2), mRNA. \| -1,80 \| \| FST \| Homo sapiens follistatin (FST), transcript variant FST317, mRNA. \| -1,80 \| \| MTFMT \| Homo sapiens mitochondrial methionyl-tRNA formyltransferase (MTFMT), mRNA. \| -1,80 \| \| --- \| Homo sapiens phosphatidylinositol-4,5-bisphosphate 3-kinase, catalytic subunit alpha (PIK3CA), mRNA. \| -1,80 \| \| --- \| RNA, U6 small nuclear 118, pseudogene [gene_biotype:snRNA transcript_biotype:snRNA] \| -1,79 \| \| --- \| PREDICTED: Homo sapiens neurofibromin 1 (NF1), transcript variant X4, mRNA. \| -1,79 \| \| RGMB \| Homo sapiens repulsive guidance molecule family member b (RGMB), mRNA. \| -1,79 \| \| ZFAS1 \| Homo sapiens ZNFX1 antisense RNA 1 (ZFAS1), transcript variant 1, long non-coding RNA. \| -1,79 \| \| EEF2KMT \| Homo sapiens eukaryotic elongation factor 2 lysine methyltransferase (EEF2KMT), transcript variant 3, mRNA. \| -1,79 \| \| --- \| Homo sapiens proline rich 13 (PRR13), transcript variant 3, mRNA. \| -1,79 \| \| GAS5 \| Homo sapiens growth arrest-specific 5 (non-protein coding) (GAS5), long non-coding RNA. \| -1,79 \| \| HIST1H1C \| Homo sapiens histone cluster 1, H1c (HIST1H1C), mRNA. \| -1,79 \| \| ELAC2 \| Homo sapiens elaC ribonuclease Z 2 (ELAC2), transcript variant 3, mRNA. \| -1,78 \| \| --- \| linc-WNT3A chr1:+:228154669-228162651 \| -1,78 \| \| MAP3K5 \| Homo sapiens mitogen-activated protein kinase kinase kinase 5 (MAP3K5), mRNA. \| -1,78 \| \| PANK3 \| Homo sapiens pantothenate kinase 3 (PANK3), mRNA. \| -1,78 \| \| MRPS10 \| Homo sapiens mitochondrial ribosomal protein S10 (MRPS10), mRNA. \| -1,78 \| \| USP14 \| Homo sapiens ubiquitin specific peptidase 14 (tRNA-guanine transglycosylase) (USP14), transcript variant 2, mRNA. \| -1,78 \| \| CYR61 \| Homo sapiens cysteine-rich, angiogenic inducer, 61 (CYR61), mRNA. \| -1,78 \| \| CHD9 \| Homo sapiens chromodomain helicase DNA binding protein 9 (CHD9), transcript variant 1, mRNA. \| -1,78 \| \| --- \| RNA, U2 small nuclear 63, pseudogene [gene_biotype:snRNA transcript_biotype:snRNA] \| -1,78 \| \| TROVE2 \| Homo sapiens TROVE domain family, member 2 (TROVE2), transcript variant 1, mRNA. \| -1,77 \| \| --- \| PREDICTED: Homo sapiens neurofibromin 1 (NF1), transcript variant X4, mRNA. \| -1,77 \| \| IL7R \| Homo sapiens interleukin 7 receptor (IL7R), transcript variant 1, mRNA. \| -1,77 \| \| CSGALNACT2 \| Homo sapiens chondroitin sulfate N-acetylgalactosaminyltransferase 2 (CSGALNACT2), mRNA. \| -1,77 \| \| ANXA2P1 \| Homo sapiens annexin A2 pseudogene 1 (ANXA2P1), non-coding RNA. \| -1,77 \| \| --- \| PREDICTED: Homo sapiens uncharacterized LOC101928451 (LOC101928451), transcript variant X1, misc_RNA. \| -1,77 \| \| --- \| small nucleolar RNA, C/D box 45B [gene_biotype:snoRNA transcript_biotype:snoRNA] \| -1,77 \| \| PHKB \| Homo sapiens phosphorylase kinase, beta (PHKB), transcript variant 1, mRNA. \| -1,77 \| \| KIAA0430 \| Homo sapiens KIAA0430 (KIAA0430), transcript variant 2, mRNA. \| -1,77 \| \| TCEB3 \| Homo sapiens transcription elongation factor B (SIII), polypeptide 3 (110kDa, elongin A) (TCEB3), mRNA. \| -1,77 \| \| SKA1 \| Homo sapiens spindle and kinetochore associated complex subunit 1 (SKA1), transcript variant 1, mRNA. \| -1,76 \| \| CENPE \| Homo sapiens centromere protein E, 312kDa (CENPE), transcript variant 2, mRNA. \| -1,76 \| \| SKA3 \| Homo sapiens spindle and kinetochore associated complex subunit 3 (SKA3), transcript variant 2, mRNA. \| -1,76 \| \| RAB12 \| Homo sapiens RAB12, member RAS oncogene family (RAB12), mRNA. \| -1,76 \| \| CWC22 \| Homo sapiens CWC22 spliceosome-associated protein (CWC22), mRNA. \| -1,76 \| \| KCTD20 \| Homo sapiens potassium channel tetramerization domain containing 20 (KCTD20), transcript variant 2, mRNA. \| -1,76 \| \| SLC35F6 \| Homo sapiens solute carrier family 35, member F6 (SLC35F6), mRNA. \| -1,76 \| \| PHACTR2 \| Homo sapiens phosphatase and actin regulator 2 (PHACTR2), transcript variant 1, mRNA. \| -1,75 \| \| --- \| Homo sapiens proline rich 13 (PRR13), transcript variant 3, mRNA. \| -1,75 \| \| ENPP2 \| Homo sapiens ectonucleotide pyrophosphatase/phosphodiesterase 2 (ENPP2), transcript variant 2, mRNA. \| -1,75 \| \| FBXO30 \| Homo sapiens F-box protein 30 (FBXO30), mRNA. \| -1,75 \| \| --- \| Homo sapiens high mobility group box 1 (HMGB1), transcript variant 3, mRNA. \| -1,75 \| \| RECK \| Homo sapiens reversion-inducing-cysteine-rich protein with kazal motifs (RECK), transcript variant 2, mRNA. \| -1,75 \| \| ROCK1 \| Homo sapiens Rho-associated, coiled-coil containing protein kinase 1 (ROCK1), mRNA. \| -1,75 \| \| OCRL \| Homo sapiens oculocerebrorenal syndrome of Lowe (OCRL), transcript variant a, mRNA. \| -1,74 \| \| KLF6 \| Homo sapiens Kruppel-like factor 6 (KLF6), transcript variant B, mRNA. \| -1,74 \| \| PRDM4 \| Homo sapiens PR domain containing 4 (PRDM4), mRNA. \| -1,74 \| \| RHOB \| Homo sapiens ras homolog family member B (RHOB), mRNA. \| -1,74 \| \| LMNB1 \| Homo sapiens lamin B1 (LMNB1), transcript variant 2, mRNA. \| -1,74 \| \| ASPM \| Homo sapiens abnormal spindle microtubule assembly (ASPM), transcript variant 2, mRNA. \| -1,74 \| \| BNIP3 \| Homo sapiens BCL2/adenovirus E1B 19kDa interacting protein 3 (BNIP3), mRNA. \| -1,74 \| \| --- \| Non-coding transcript identified by NONCODE \| -1,74 \| \| --- \| Non-coding transcript identified by NONCODE \| -1,73 \| \| SLBP \| Homo sapiens stem-loop binding protein (SLBP), transcript variant 2, mRNA. \| -1,73 \| \| --- \| Metastasis-associated lung adenocarcinoma transcript 1 [gene_biotype:protein_coding transcript_biotype:protein_coding] \| -1,73 \| \| LINC00657 \| Homo sapiens long intergenic non-protein coding RNA 657 (LINC00657), long non-coding RNA. \| -1,73 \| \| --- \| Homo sapiens B-cell receptor-associated protein 31 (BCAP31), transcript variant 3, mRNA. \| -1,73 \| \| RNU4ATAC \| Homo sapiens RNA, U4atac small nuclear (U12-dependent splicing) (RNU4ATAC), small nuclear RNA. \| -1,73 \| \| --- \| Homo sapiens small nucleolar RNA, C/D box 80 (SNORD80), small nucleolar RNA. \| -1,73 \| \| ZWILCH \| Homo sapiens zwilch kinetochore protein (ZWILCH), transcript variant 2, mRNA. \| -1,73 \| \| --- \| RNA, 5S ribosomal pseudogene 60 [gene_biotype:rRNA transcript_biotype:rRNA] \| -1,73 \| \| LINC01061 \| Homo sapiens piRNA piR-37834, complete sequence. \| -1,73 \| \| --- \| Homo sapiens mitofusin 1 (MFN1), mRNA. \| -1,73 \| \| NBPF20 \| Homo sapiens neuroblastoma breakpoint family, member 20 (NBPF20), mRNA. \| -1,73 \| \| HSBP1L1 \| Homo sapiens heat shock factor binding protein 1-like 1 (HSBP1L1), mRNA. \| -1,73 \| \| --- \| solute carrier family 25 (mitochondrial carrier; adenine nucleotide translocator), member 5 pseudogene 5 [gene_biotype:processed_pseudogene transcript_biotype:processed_pseudogene] \| -1,72 \| \| --- \| Homo sapiens chondroitin sulfate proteoglycan 4 (CSPG4), mRNA. \| -1,72 \| \| DHX40 \| Homo sapiens DEAH (Asp-Glu-Ala-His) box polypeptide 40 (DHX40), transcript variant 2, mRNA. \| -1,72 \| \| AHNAK \| Homo sapiens AHNAK nucleoprotein (AHNAK), transcript variant 1, mRNA. \| -1,72 \| \| PAMR1 \| Homo sapiens peptidase domain containing associated with muscle regeneration 1 (PAMR1), transcript variant 2, mRNA. \| -1,72 \| \| RPL23AP60 \| ribosomal protein L23a pseudogene 60[gene_biotype:processed_pseudogene transcript_biotype:processed_pseudogene] \| -1,72 \| \| RPL23AP60 \| ribosomal protein L23a pseudogene 60[gene_biotype:processed_pseudogene transcript_biotype:processed_pseudogene] \| -1,72 \| \| RPL23AP60 \| ribosomal protein L23a pseudogene 60[gene_biotype:processed_pseudogene transcript_biotype:processed_pseudogene] \| -1,72 \| \| ARHGAP11A \| Homo sapiens Rho GTPase activating protein 11A (ARHGAP11A), transcript variant 3, mRNA. \| -1,72 \| \| DKK1 \| Homo sapiens dickkopf WNT signaling pathway inhibitor 1 (DKK1), mRNA. \| -1,72 \| \| VTI1B \| Homo sapiens vesicle transport through interaction with t-SNAREs 1B (VTI1B), mRNA. \| -1,72 \| \| --- \| Homo sapiens septin 7 pseudogene 2 (SEPT7P2), non-coding RNA. \| -1,71 \| \| ADAMTS1 \| Homo sapiens ADAM metallopeptidase with thrombospondin type 1 motif, 1 (ADAMTS1), mRNA. \| -1,71 \| \| --- \| linc-KRTAP9-1 chr17:+:39264469-39272256 \| -1,71 \| \| NEDD1 \| Homo sapiens neural precursor cell expressed, developmentally down-regulated 1 (NEDD1), transcript variant 1, mRNA. \| -1,71 \| \| --- \| Non-coding transcript identified by NONCODE \| -1,71 \| \| PAK1IP1 \| Homo sapiens PAK1 interacting protein 1 (PAK1IP1), mRNA. \| -1,71 \| \| --- \| Homo sapiens chloride channel, voltage-sensitive 3 (CLCN3), transcript variant a, mRNA. \| -1,71 \| \| UBA2 \| Homo sapiens ubiquitin-like modifier activating enzyme 2 (UBA2), mRNA. \| -1,71 \| \| NEXN \| Homo sapiens nexilin (F actin binding protein) (NEXN), transcript variant 2, mRNA. \| -1,71 \| \| MARCH6 \| Homo sapiens membrane-associated ring finger (C3HC4) 6, E3 ubiquitin protein ligase (MARCH6), transcript variant 2, mRNA. \| -1,71 \| \| DCUN1D1 \| Homo sapiens DCN1, defective in cullin neddylation 1, domain containing 1 (DCUN1D1), transcript variant 2, mRNA. \| -1,71 \| \| SNORA71A \| Homo sapiens small nucleolar RNA, H/ACA box 71A (SNORA71A), small nucleolar RNA. \| -1,71 \| \| GTF2IP4 \| Homo sapiens general transcription factor IIi, pseudogene 4 (GTF2IP4), non-coding RNA. \| -1,71 \| \| RBMS3 \| Homo sapiens RNA binding motif, single stranded interacting protein 3 (RBMS3), transcript variant 3, mRNA. \| -1,71 \| \| --- \| Homo sapiens SMG1 pseudogene 7 (SMG1P7), non-coding RNA. \| -1,70 \| \| ANGPT1 \| Homo sapiens angiopoietin 1 (ANGPT1), transcript variant 1, mRNA. \| -1,70 \| \| WDR44 \| Homo sapiens WD repeat domain 44 (WDR44), transcript variant 2, mRNA. \| -1,70 \| \| TRIM8 \| Homo sapiens tripartite motif containing 8 (TRIM8), mRNA. \| -1,70 \| \| SMYD2 \| Homo sapiens SET and MYND domain containing 2 (SMYD2), mRNA. \| -1,70 \| \| TUBGCP4 \| Homo sapiens tubulin, gamma complex associated protein 4 (TUBGCP4), transcript variant 1, mRNA. \| -1,70 \| \| --- \| Homo sapiens long intergenic non-protein coding RNA 674 (LINC00674), long non-coding RNA. \| -1,70 \| \| --- \| long intergenic non-protein coding RNA 665 [gene_biotype:lincRNA transcript_biotype:lincRNA] \| -1,70 \| \| FOS \| Homo sapiens FBJ murine osteosarcoma viral oncogene homolog (FOS), mRNA. \| -1,70 \| \| CENPI \| Homo sapiens centromere protein I (CENPI), mRNA. \| -1,70 \| \| ODC1 \| Homo sapiens ornithine decarboxylase 1 (ODC1), transcript variant 2, mRNA. \| -1,69 \| \| RAB8B \| Homo sapiens RAB8B, member RAS oncogene family (RAB8B), mRNA. \| -1,69 \| \| ATP11C \| Homo sapiens ATPase, class VI, type 11C (ATP11C), transcript variant 2, mRNA. \| -1,69 \| \| TMSB4X \| Homo sapiens thymosin beta 4, X-linked (TMSB4X), mRNA. \| -1,69 \| \| NDC1 \| Homo sapiens NDC1 transmembrane nucleoporin (NDC1), transcript variant 2, mRNA. \| -1,69 \| \| PLSCR4 \| Homo sapiens phospholipid scramblase 4 (PLSCR4), transcript variant 1, mRNA. \| -1,69 \| \| HIST1H4K \| Homo sapiens histone cluster 1, H4k (HIST1H4K), mRNA. \| -1,69 \| \| HERC4 \| Homo sapiens HECT and RLD domain containing E3 ubiquitin protein ligase 4 (HERC4), transcript variant 3, mRNA. \| -1,69 \| \| UBALD2 \| Homo sapiens UBA-like domain containing 2 (UBALD2), mRNA. \| -1,69 \| \| NF1 \| Homo sapiens neurofibromin 1 (NF1), transcript variant 2, mRNA. \| -1,69 \| \| SBNO1 \| Homo sapiens strawberry notch homolog 1 (Drosophila) (SBNO1), transcript variant 1, mRNA. \| -1,69 \| \| BROX \| Homo sapiens BRO1 domain and CAAX motif containing (BROX), transcript variant 2, mRNA. \| -1,69 \| \| KIAA1462 \| Homo sapiens KIAA1462 (KIAA1462), mRNA. \| -1,69 \| \| LOC105369807 \| PREDICTED: Homo sapiens uncharacterized LOC105369807 (LOC105369807), transcript variant X1, ncRNA. \| -1,69 \| \| --- \| Non-coding transcript identified by NONCODE \| -1,69 \| \| ROCK2 \| Homo sapiens Rho-associated, coiled-coil containing protein kinase 2 (ROCK2), mRNA. \| -1,69 \| \| --- \| Homo sapiens VPS35 retromer complex component (VPS35), mRNA. \| -1,69 \| \| DLGAP5 \| Homo sapiens discs, large (Drosophila) homolog-associated protein 5 (DLGAP5), transcript variant 2, mRNA. \| -1,69 \| \| CTNNAL1 \| Homo sapiens catenin (cadherin-associated protein), alpha-like 1 (CTNNAL1), transcript variant 2, mRNA. \| -1,69 \| \| TPR \| Homo sapiens translocated promoter region, nuclear basket protein (TPR), mRNA. \| -1,68 \| \| SLIT2 \| Homo sapiens slit guidance ligand 2 (SLIT2), transcript variant 2, mRNA. \| -1,68 \| \| HSDL1 \| Homo sapiens hydroxysteroid dehydrogenase like 1 (HSDL1), transcript variant 2, mRNA. \| -1,68 \| \| CCNG1 \| Homo sapiens cyclin G1 (CCNG1), transcript variant 1, mRNA. \| -1,68 \| \| --- \| Homo sapiens microRNA 423 (MIR423), microRNA. \| -1,68 \| \| SLC33A1 \| Homo sapiens solute carrier family 33 (acetyl-CoA transporter), member 1 (SLC33A1), transcript variant 2, mRNA. \| -1,68 \| \| PTPRM \| Homo sapiens protein tyrosine phosphatase, receptor type, M (PTPRM), transcript variant 1, mRNA. \| -1,68 \| \| NUP155 \| Homo sapiens nucleoporin 155kDa (NUP155), transcript variant 3, mRNA. \| -1,68 \| \| FBXL20 \| Homo sapiens F-box and leucine-rich repeat protein 20 (FBXL20), transcript variant 2, mRNA. \| -1,68 \| \| PRRX2 \| Homo sapiens paired related homeobox 2 (PRRX2), mRNA. \| -1,68 \| \| STAG2 \| Homo sapiens stromal antigen 2 (STAG2), transcript variant 1, mRNA. \| -1,68 \| \| USP16 \| Homo sapiens ubiquitin specific peptidase 16 (USP16), transcript variant 2, mRNA. \| -1,67 \| \| VAMP4 \| Homo sapiens vesicle-associated membrane protein 4 (VAMP4), transcript variant 2, mRNA. \| -1,67 \| \| MED1 \| Homo sapiens mediator complex subunit 1 (MED1), mRNA. \| -1,67 \| \| GHITM \| Homo sapiens growth hormone inducible transmembrane protein (GHITM), mRNA. \| -1,67 \| \| PSAT1 \| Homo sapiens phosphoserine aminotransferase 1 (PSAT1), transcript variant 2, mRNA. \| -1,67 \| \| TGFB2 \| Homo sapiens transforming growth factor, beta 2 (TGFB2), transcript variant 1, mRNA. \| -1,67 \| \| --- \| PREDICTED: Homo sapiens family with sequence similarity 157, member B (FAM157B), transcript variant X7, mRNA. \| -1,67 \| \| HIST1H3G \| Homo sapiens histone cluster 1, H3g (HIST1H3G), mRNA. \| -1,67 \| \| --- \| Homo sapiens small nucleolar RNA, C/D box 54 (SNORD54), small nucleolar RNA. \| -1,67 \| \| --- \| Non-coding transcript identified by NONCODE \| -1,67 \| \| SLC4A4 \| Homo sapiens solute carrier family 4 (sodium bicarbonate cotransporter), member 4 (SLC4A4), transcript variant 1, mRNA. \| -1,67 \| \| KIAA0196 \| Homo sapiens KIAA0196 (KIAA0196), mRNA. \| -1,67 \| \| LINC00294 \| Homo sapiens long intergenic non-protein coding RNA 294 (LINC00294), long non-coding RNA. \| -1,67 \| \| ADRA2C \| Homo sapiens adrenoceptor alpha 2C (ADRA2C), mRNA. \| -1,67 \| \| PRKAR2A \| Homo sapiens protein kinase, cAMP-dependent, regulatory, type II, alpha (PRKAR2A), mRNA. \| -1,67 \| \| LINC01061 \| Homo sapiens long intergenic non-protein coding RNA 1061 (LINC01061), long non-coding RNA. \| -1,67 \| \| FAM171A1 \| Homo sapiens family with sequence similarity 171, member A1 (FAM171A1), mRNA. \| -1,67 \| \| CKAP5 \| Homo sapiens cytoskeleton associated protein 5 (CKAP5), transcript variant 1, mRNA. \| -1,66 \| \| SOAT1 \| Homo sapiens sterol O-acyltransferase 1 (SOAT1), transcript variant 2, mRNA. \| -1,66 \| \| OTUD6B-AS1 \| Homo sapiens OTUD6B antisense RNA 1 (head to head) (OTUD6B-AS1), transcript variant 1, long non-coding RNA. \| -1,66 \| \| PIGK \| Homo sapiens phosphatidylinositol glycan anchor biosynthesis, class K (PIGK), mRNA. \| -1,66 \| \| PIAS1 \| Homo sapiens protein inhibitor of activated STAT, 1 (PIAS1), mRNA. \| -1,66 \| \| AIM1 \| Homo sapiens absent in melanoma 1 (AIM1), mRNA. \| -1,66 \| \| GPATCH8 \| Homo sapiens G patch domain containing 8 (GPATCH8), transcript variant 1, mRNA. \| -1,66 \| \| --- \| Homo sapiens mRNA; cDNA DKFZp686K16201 (from clone DKFZp686K16201). \| -1,66 \| \| --- \| growth arrest-specific 5 (non-protein coding) [gene_biotype:processed_transcript transcript_biotype:retained_intron] \| -1,66 \| \| PUM2 \| Homo sapiens pumilio RNA-binding family member 2 (PUM2), transcript variant 2, mRNA. \| -1,66 \| \| --- \| cdna:genscan chromosome:GRCh38:20:21755177:21755536:-1 transcript_biotype:protein_coding \| -1,66 \| \| MCM4 \| Homo sapiens minichromosome maintenance complex component 4 (MCM4), transcript variant 1, mRNA. \| -1,66 \| \| WDR75 \| Homo sapiens WD repeat domain 75 (WDR75), transcript variant 2, mRNA. \| -1,65 \| \| NOA1 \| Homo sapiens nitric oxide associated 1 (NOA1), mRNA. \| -1,65 \| \| NAA50 \| Homo sapiens N(alpha)-acetyltransferase 50, NatE catalytic subunit (NAA50), transcript variant 2, mRNA. \| -1,65 \| \| --- \| Homo sapiens golgin A8 family, member A (GOLGA8A), transcript variant 2, non-coding RNA. \| -1,65 \| \| DDX27 \| Homo sapiens DEAD (Asp-Glu-Ala-Asp) box polypeptide 27 (DDX27), mRNA. \| -1,65 \| \| --- \| mitochondrially encoded tRNA asparagine [gene_biotype:Mt_tRNA transcript_biotype:Mt_tRNA] \| -1,65 \| \| --- \| Small nucleolar RNA U13 [gene_biotype:snoRNA transcript_biotype:snoRNA] \| -1,65 \| \| NFIB \| Homo sapiens nuclear factor I/B (NFIB), transcript variant 1, mRNA. \| -1,65 \| \| SRPX \| Homo sapiens sushi-repeat containing protein, X-linked (SRPX), transcript variant 2, mRNA. \| -1,65 \| \| ID3 \| Homo sapiens inhibitor of DNA binding 3, dominant negative helix-loop-helix protein (ID3), mRNA. \| -1,65 \| \| SPTLC3 \| Homo sapiens serine palmitoyltransferase, long chain base subunit 3 (SPTLC3), mRNA. \| -1,65 \| \| DNAJC13 \| Homo sapiens DnaJ (Hsp40) homolog, subfamily C, member 13 (DNAJC13), mRNA. \| -1,65 \| \| LOC100133331 \| Homo sapiens uncharacterized LOC100133331 (LOC100133331), long non-coding RNA. \| -1,65 \| \| DROSHA \| Homo sapiens drosha, ribonuclease type III (DROSHA), transcript variant 2, mRNA. \| -1,65 \| \| FAM83D \| Homo sapiens family with sequence similarity 83, member D (FAM83D), mRNA. \| -1,65 \| \| FGF5 \| Homo sapiens fibroblast growth factor 5 (FGF5), transcript variant 3, mRNA. \| -1,65 \| \| CRIM1 \| Homo sapiens cysteine rich transmembrane BMP regulator 1 (chordin-like) (CRIM1), mRNA. \| -1,65 \| \| QSER1 \| Homo sapiens glutamine and serine rich 1 (QSER1), mRNA. \| -1,64 \| \| ZBED6 \| Homo sapiens zinc finger, BED-type containing 6 (ZBED6), mRNA. \| -1,64 \| \| PXK \| Homo sapiens PX domain containing serine/threonine kinase (PXK), transcript variant 2, mRNA. \| -1,64 \| \| --- \| Homo sapiens mRNA; cDNA DKFZp686K16201 (from clone DKFZp686K16201). \| -1,64 \| \| WDFY3 \| Homo sapiens WD repeat and FYVE domain containing 3 (WDFY3), mRNA. \| -1,64 \| \| MMD \| Homo sapiens monocyte to macrophage differentiation-associated (MMD), mRNA. \| -1,64 \| \| --- \| Homo sapiens mRNA; cDNA DKFZp686K16201 (from clone DKFZp686K16201). \| -1,64 \| \| --- \| Homo sapiens armadillo repeat containing 10 (ARMC10), transcript variant B, mRNA. \| -1,63 \| \| NOMO1 \| Homo sapiens NODAL modulator 1 (NOMO1), mRNA. \| -1,63 \| \| PDCD1LG2 \| Homo sapiens programmed cell death 1 ligand 2 (PDCD1LG2), mRNA. \| -1,63 \| \| --- \| Homo sapiens GATS protein-like 1 (GATSL1), mRNA. \| -1,63 \| \| --- \| Homo sapiens ankyrin repeat domain 36 (ANKRD36), mRNA. \| -1,63 \| \| TBCB \| Homo sapiens tubulin folding cofactor B (TBCB), transcript variant 1, mRNA. \| -1,63 \| \| MTMR6 \| Homo sapiens myotubularin related protein 6 (MTMR6), mRNA. \| -1,63 \| \| MBOAT2 \| Homo sapiens membrane bound O-acyltransferase domain containing 2 (MBOAT2), mRNA. \| -1,63 \| \| TMEM33 \| Homo sapiens transmembrane protein 33 (TMEM33), mRNA. \| -1,63 \| \| UBA6 \| Homo sapiens ubiquitin-like modifier activating enzyme 6 (UBA6), mRNA. \| -1,63 \| \| --- \| Homo sapiens SMG1 phosphatidylinositol 3-kinase-related kinase (SMG1), mRNA. \| -1,63 \| \| --- \| PREDICTED: Homo sapiens uncharacterized LOC101928451 (LOC101928451), transcript variant X1, misc_RNA. \| -1,63 \| \| TERC \| Homo sapiens telomerase RNA component (TERC), telomerase RNA. \| -1,62 \| \| GPCPD1 \| Homo sapiens glycerophosphocholine phosphodiesterase 1 (GPCPD1), mRNA. \| -1,62 \| \| CCBE1 \| Homo sapiens collagen and calcium binding EGF domains 1 (CCBE1), mRNA. \| -1,62 \| \| MAP4K4 \| Homo sapiens mitogen-activated protein kinase kinase kinase kinase 4 (MAP4K4), transcript variant 4, mRNA. \| -1,62 \| \| TAF1D \| TATA box binding protein (TBP)-associated factor, RNA polymerase I, D, 41kDa [gene_biotype:protein_coding transcript_biotype:nonsense_mediated_decay] \| -1,62 \| \| CDC42BPB \| Homo sapiens CDC42 binding protein kinase beta (DMPK-like) (CDC42BPB), mRNA. \| -1,62 \| \| EPHX1 \| Homo sapiens epoxide hydrolase 1, microsomal (xenobiotic) (EPHX1), transcript variant 1, mRNA. \| -1,62 \| \| MYDGF \| Homo sapiens myeloid-derived growth factor (MYDGF), mRNA. \| -1,62 \| \| IGF2R \| Homo sapiens insulin-like growth factor 2 receptor (IGF2R), mRNA. \| -1,62 \| \| MTX2 \| Homo sapiens metaxin 2 (MTX2), transcript variant 1, mRNA. \| -1,62 \| \| NARS \| Homo sapiens asparaginyl-tRNA synthetase (NARS), mRNA. \| -1,62 \| \| SNORD114-14 \| Homo sapiens small nucleolar RNA, C/D box 114-14 (SNORD114-14), small nucleolar RNA. \| -1,62 \| \| OGFRL1 \| Homo sapiens opioid growth factor receptor-like 1 (OGFRL1), mRNA. \| -1,62 \| \| IMPACT \| Homo sapiens impact RWD domain protein (IMPACT), mRNA. \| -1,62 \| \| EFEMP1 \| Homo sapiens EGF containing fibulin-like extracellular matrix protein 1 (EFEMP1), transcript variant 2, mRNA. \| -1,62 \| \| FAM192A \| Homo sapiens family with sequence similarity 192, member A (FAM192A), mRNA. \| -1,61 \| \| CNIH1 \| Homo sapiens cornichon family AMPA receptor auxiliary protein 1 (CNIH1), mRNA. \| -1,61 \| \| AIMP1 \| Homo sapiens aminoacyl tRNA synthetase complex-interacting multifunctional protein 1 (AIMP1), transcript variant 2, mRNA. \| -1,61 \| \| KAT6B \| Homo sapiens K(lysine) acetyltransferase 6B (KAT6B), transcript variant 2, mRNA. \| -1,61 \| \| KMT2E \| Homo sapiens lysine (K)-specific methyltransferase 2E (KMT2E), transcript variant 2, mRNA. \| -1,61 \| \| AP1M1 \| Homo sapiens adaptor-related protein complex 1, mu 1 subunit (AP1M1), transcript variant 1, mRNA. \| -1,61 \| \| --- \| Homo sapiens phosphatidylinositol-4,5-bisphosphate 3-kinase, catalytic subunit alpha (PIK3CA), mRNA. \| -1,61 \| \| TMEM183B \| Homo sapiens transmembrane protein 183B (TMEM183B), mRNA. \| -1,61 \| \| NIPAL3 \| Homo sapiens NIPA-like domain containing 3 (NIPAL3), mRNA. \| -1,61 \| \| DDR2 \| Homo sapiens discoidin domain receptor tyrosine kinase 2 (DDR2), transcript variant 1, mRNA. \| -1,61 \| \| SKA2 \| Homo sapiens spindle and kinetochore associated complex subunit 2 (SKA2), transcript variant 2, mRNA. \| -1,61 \| \| POLR2C \| Homo sapiens polymerase (RNA) II (DNA directed) polypeptide C, 33kDa (POLR2C), mRNA. \| -1,61 \| \| --- \| Non-coding transcript identified by NONCODE \| -1,61 \| \| SGPL1 \| Homo sapiens sphingosine-1-phosphate lyase 1 (SGPL1), mRNA. \| -1,61 \| \| DOCK1 \| Homo sapiens dedicator of cytokinesis 1 (DOCK1), transcript variant 1, mRNA. \| -1,61 \| \| KLHL5 \| Homo sapiens kelch-like family member 5 (KLHL5), transcript variant 3, mRNA. \| -1,61 \| \| DUSP10 \| Homo sapiens dual specificity phosphatase 10 (DUSP10), transcript variant 1, mRNA. \| -1,60 \| \| --- \| Small nucleolar RNA U3 [gene_biotype:snoRNA transcript_biotype:snoRNA] \| -1,60 \| \| LOC105371220 \| PREDICTED: Homo sapiens uncharacterized LOC105371220 (LOC105371220), ncRNA. \| -1,60 \| \| PPP4R1 \| Homo sapiens protein phosphatase 4, regulatory subunit 1 (PPP4R1), transcript variant 1, mRNA. \| -1,60 \| \| UBE2J1 \| Homo sapiens ubiquitin-conjugating enzyme E2, J1 (UBE2J1), mRNA. \| -1,60 \| \| TOMM22 \| Homo sapiens translocase of outer mitochondrial membrane 22 homolog (yeast) (TOMM22), mRNA. \| -1,60 \| \| SLC9A7 \| Homo sapiens solute carrier family 9, subfamily A (NHE7, cation proton antiporter 7), member 7 (SLC9A7), transcript variant 1, mRNA. \| -1,60 \| \| JAK2 \| Homo sapiens Janus kinase 2 (JAK2), mRNA. \| -1,60 \| \| --- \| ncrna:novel chromosome:GRCh38:4:153873367:153873451:-1 gene:ENSG00000252181 gene_biotype:miRNA transcript_biotype:miRNA \| -1,60 \| \| --- \| ncrna:novel chromosome:GRCh38:4:153873367:153873451:-1 gene:ENSG00000252181 gene_biotype:miRNA transcript_biotype:miRNA \| -1,60 \| \| NEDD4L \| Homo sapiens neural precursor cell expressed, developmentally down-regulated 4-like, E3 ubiquitin protein ligase (NEDD4L), transcript variant b, mRNA. \| -1,60 \| \| --- \| Homo sapiens filamin C, gamma (FLNC), transcript variant 2, mRNA. \| -1,60 \| \| --- \| havana:known chromosome:GRCh38:11:74745716:74746114:-1 gene:ENSG00000227615 gene_biotype:processed_pseudogene transcript_biotype:processed_pseudogene \| -1,60 \| \| LOC100288069 \| Homo sapiens uncharacterized LOC100288069 (LOC100288069), long non-coding RNA. \| -1,60 \| \| SNORD61 \| Homo sapiens small nucleolar RNA, C/D box 61 (SNORD61), small nucleolar RNA. \| -1,60 \| \| ZNF532 \| Homo sapiens zinc finger protein 532 (ZNF532), mRNA. \| -1,60 \| \| --- \| Homo sapiens major histocompatibility complex, class II, DR alpha (HLA-DRA), mRNA. \| -1,60 \| \| NOL11 \| Homo sapiens nucleolar protein 11 (NOL11), transcript variant 2, mRNA. \| -1,60 \| \| FANCI \| Homo sapiens Fanconi anemia, complementation group I (FANCI), transcript variant 1, mRNA. \| -1,60 \| \| RBL1 \| Homo sapiens retinoblastoma-like 1 (RBL1), transcript variant 1, mRNA. \| -1,60 \| \| MASTL \| Homo sapiens microtubule associated serine/threonine kinase-like (MASTL), transcript variant 1, mRNA. \| -1,60 \| \| --- \| nicotinamide phosphoribosyltransferase pseudogene 1 [gene_biotype:processed_pseudogene transcript_biotype:processed_pseudogene] \| -1,60 \| \| CKAP2L \| Homo sapiens cytoskeleton associated protein 2-like (CKAP2L), transcript variant 2, mRNA. \| -1,60 \| \| PABPC4 \| Homo sapiens poly(A) binding protein, cytoplasmic 4 (inducible form) (PABPC4), transcript variant 1, mRNA. \| -1,60 \| \| POLH \| Homo sapiens polymerase (DNA directed), eta (POLH), transcript variant 2, mRNA. \| -1,60 \| \| KCTD9 \| Homo sapiens potassium channel tetramerization domain containing 9 (KCTD9), mRNA. \| -1,59 \| \| HMGN1P30 \| high mobility group nucleosome binding domain 1 pseudogene 30[gene_biotype:processed_pseudogene transcript_biotype:processed_pseudogene] \| -1,59 \| \| LY6K \| Homo sapiens lymphocyte antigen 6 complex, locus K (LY6K), transcript variant 2, mRNA. \| -1,59 \| \| NEMP1 \| Homo sapiens nuclear envelope integral membrane protein 1 (NEMP1), transcript variant 1, mRNA. \| -1,59 \| \| ABI3BP \| Homo sapiens ABI family, member 3 (NESH) binding protein (ABI3BP), mRNA. \| -1,59 \| \| PPIG \| Homo sapiens peptidylprolyl isomerase G (cyclophilin G) (PPIG), mRNA. \| -1,59 \| \| BMPR1A \| Homo sapiens bone morphogenetic protein receptor, type IA (BMPR1A), mRNA. \| -1,59 \| \| PIK3CA \| Homo sapiens phosphatidylinositol-4,5-bisphosphate 3-kinase, catalytic subunit alpha (PIK3CA), mRNA. \| -1,59 \| \| FAM20C \| Homo sapiens family with sequence similarity 20, member C (FAM20C), mRNA. \| -1,59 \| \| PPP2R5E \| Homo sapiens protein phosphatase 2, regulatory subunit B, epsilon isoform (PPP2R5E), transcript variant 2, mRNA. \| -1,59 \| \| LOC728026 \| PREDICTED: Homo sapiens prothymosin alpha-like (LOC728026), transcript variant X2, mRNA. \| -1,59 \| \| HIST1H3B \| Homo sapiens histone cluster 1, H3b (HIST1H3B), mRNA. \| -1,59 \| \| MALT1 \| Homo sapiens MALT1 paracaspase (MALT1), transcript variant 1, mRNA. \| -1,59 \| \| FBXO11 \| Homo sapiens F-box protein 11 (FBXO11), transcript variant 4, mRNA. \| -1,59 \| \| ENTPD6 \| Homo sapiens ectonucleoside triphosphate diphosphohydrolase 6 (putative) (ENTPD6), transcript variant 2, mRNA. \| -1,59 \| \| RND3 \| Homo sapiens Rho family GTPase 3 (RND3), transcript variant 1, mRNA. \| -1,59 \| \| VIM-AS1 \| Homo sapiens VIM antisense RNA 1 (VIM-AS1), transcript variant 1, long non-coding RNA. \| -1,59 \| \| SECTM1 \| Homo sapiens secreted and transmembrane 1 (SECTM1), mRNA. \| -1,59 \| \| RNPS1 \| Homo sapiens RNA binding protein S1, serine-rich domain (RNPS1), transcript variant 3, mRNA. \| -1,59 \| \| GABARAPL2 \| Homo sapiens GABA(A) receptor-associated protein like 2 (GABARAPL2), mRNA. \| -1,58 \| \| ENC1 \| Homo sapiens ectodermal-neural cortex 1 (with BTB domain) (ENC1), transcript variant 2, mRNA. \| -1,58 \| \| UBE2T \| Homo sapiens ubiquitin-conjugating enzyme E2T (UBE2T), transcript variant 2, mRNA. \| -1,58 \| \| --- \| Homo sapiens flotillin 1 (FLOT1), mRNA. \| -1,58 \| \| RAB5C \| Homo sapiens RAB5C, member RAS oncogene family (RAB5C), transcript variant 3, mRNA. \| -1,58 \| \| DIAPH2 \| Homo sapiens diaphanous-related formin 2 (DIAPH2), transcript variant 156, mRNA. \| -1,58 \| \| --- \| Non-coding transcript identified by NONCODE \| -1,58 \| \| FYTTD1 \| Homo sapiens forty-two-three domain containing 1 (FYTTD1), transcript variant 2, mRNA. \| -1,58 \| \| RALBP1 \| Homo sapiens ralA binding protein 1 (RALBP1), mRNA. \| -1,58 \| \| --- \| Homo sapiens YTH domain containing 1 (YTHDC1), transcript variant 1, mRNA. \| -1,58 \| \| --- \| Homo sapiens small nucleolar RNA, C/D box 42B (SNORD42B), small nucleolar RNA. \| -1,58 \| \| --- \| havana:known chromosome:GRCh38:14:90402523:90405235:-1 gene:ENSG00000258424 gene_biotype:antisense transcript_biotype:antisense \| -1,58 \| \| --- \| Homo sapiens major histocompatibility complex, class II, DR alpha (HLA-DRA), mRNA. \| -1,58 \| \| --- \| Homo sapiens major histocompatibility complex, class II, DR alpha (HLA-DRA), mRNA. \| -1,58 \| \| --- \| Homo sapiens major histocompatibility complex, class II, DR alpha (HLA-DRA), mRNA. \| -1,58 \| \| MPI \| Homo sapiens mannose phosphate isomerase (MPI), transcript variant 2, mRNA. \| -1,58 \| \| ATN1 \| Homo sapiens atrophin 1 (ATN1), transcript variant 1, mRNA. \| -1,58 \| \| SLC9A7 \| Homo sapiens solute carrier family 9, subfamily A (NHE7, cation proton antiporter 7), member 7 (SLC9A7), transcript variant 1, mRNA. \| -1,58 \| \| ARHGAP18 \| Homo sapiens Rho GTPase activating protein 18 (ARHGAP18), mRNA. \| -1,58 \| \| NPIPB4 \| Homo sapiens nuclear pore complex interacting protein family, member B4 (NPIPB4), mRNA. \| -1,58 \| \| ASAP1 \| Homo sapiens ArfGAP with SH3 domain, ankyrin repeat and PH domain 1 (ASAP1), transcript variant 2, mRNA. \| -1,58 \| \| HCFC2 \| Homo sapiens host cell factor C2 (HCFC2), mRNA. \| -1,58 \| \| TCTN2 \| Homo sapiens tectonic family member 2 (TCTN2), transcript variant 2, mRNA. \| -1,58 \| \| BPTF \| Homo sapiens bromodomain PHD finger transcription factor (BPTF), transcript variant 2, mRNA. \| -1,58 \| \| --- \| RNA, U1 small nuclear 42, pseudogene [gene_biotype:snRNA transcript_biotype:snRNA] \| -1,58 \| \| WWC2 \| Homo sapiens WW and C2 domain containing 2 (WWC2), mRNA. \| -1,58 \| \| REEP3 \| Homo sapiens receptor accessory protein 3 (REEP3), mRNA. \| -1,58 \| \| FOSL1 \| Homo sapiens FOS-like antigen 1 (FOSL1), transcript variant 2, mRNA. \| -1,57 \| \| GTF2IP20 \| Homo sapiens general transcription factor IIi pseudogene 20 (GTF2IP20), non-coding RNA. \| -1,57 \| \| --- \| Homo sapiens golgin A8 family, member A (GOLGA8A), transcript variant 2, non-coding RNA. \| -1,57 \| \| --- \| RNA, U6 small nuclear 781, pseudogene [gene_biotype:snRNA transcript_biotype:snRNA] \| -1,57 \| \| UBE2A \| Homo sapiens ubiquitin-conjugating enzyme E2A (UBE2A), transcript variant 4, mRNA. \| -1,57 \| \| NDC80 \| Homo sapiens NDC80 kinetochore complex component (NDC80), mRNA. \| -1,57 \| \| LOC101927999 \| PREDICTED: Homo sapiens putative uncharacterized protein encoded by LINC00174 (LOC101927999), misc_RNA. \| -1,57 \| \| --- \| Non-coding transcript identified by NONCODE \| -1,57 \| \| ARHGAP12 \| Homo sapiens Rho GTPase activating protein 12 (ARHGAP12), transcript variant 2, mRNA. \| -1,57 \| \| MEA1 \| Homo sapiens male-enhanced antigen 1 (MEA1), mRNA. \| -1,57 \| \| --- \| havana:known chromosome:GRCh38:12:76030494:76031378:1 gene:ENSG00000257453 gene_biotype:antisense transcript_biotype:antisense \| -1,57 \| \| USP8 \| Homo sapiens ubiquitin specific peptidase 8 (USP8), transcript variant 2, mRNA. \| -1,57 \| \| FAM160B1 \| Homo sapiens family with sequence similarity 160, member B1 (FAM160B1), transcript variant 2, mRNA. \| -1,57 \| \| TRIP13 \| Homo sapiens thyroid hormone receptor interactor 13 (TRIP13), transcript variant 2, mRNA. \| -1,57 \| \| PUS7L \| Homo sapiens pseudouridylate synthase 7-like (PUS7L), transcript variant 2, mRNA. \| -1,57 \| \| LRIF1 \| Homo sapiens ligand dependent nuclear receptor interacting factor 1 (LRIF1), transcript variant 2, mRNA. \| -1,57 \| \| EFR3A \| Homo sapiens EFR3 homolog A (EFR3A), mRNA. \| -1,57 \| \| --- \| Homo sapiens small nucleolar RNA, C/D box 24 (SNORD24), small nucleolar RNA. \| -1,57 \| \| ZFYVE16 \| Homo sapiens zinc finger, FYVE domain containing 16 (ZFYVE16), transcript variant 2, mRNA. \| -1,57 \| \| YIPF6 \| Homo sapiens Yip1 domain family, member 6 (YIPF6), transcript variant B, mRNA. \| -1,57 \| \| TAF1D \| Homo sapiens TATA box binding protein (TBP)-associated factor, RNA polymerase I, D, 41kDa (TAF1D), mRNA. \| -1,57 \| \| --- \| growth arrest-specific 5 (non-protein coding) [gene_biotype:processed_transcript transcript_biotype:retained_intron] \| -1,57 \| \| HIST1H4L \| Homo sapiens histone cluster 1, H4l (HIST1H4L), mRNA. \| -1,57 \| \| B4GALT5 \| Homo sapiens UDP-Gal:betaGlcNAc beta 1,4- galactosyltransferase, polypeptide 5 (B4GALT5), mRNA. \| -1,57 \| \| MEIS3P1 \| Homo sapiens Meis homeobox 3 pseudogene 1 (MEIS3P1), non-coding RNA. \| -1,57 \| \| PHIP \| Homo sapiens pleckstrin homology domain interacting protein (PHIP), mRNA. \| -1,57 \| \| GOPC \| Homo sapiens golgi-associated PDZ and coiled-coil motif containing (GOPC), transcript variant 2, mRNA. \| -1,57 \| \| THRA \| Homo sapiens thyroid hormone receptor, alpha (THRA), transcript variant 3, mRNA. \| -1,57 \| \| SLC30A7 \| Homo sapiens solute carrier family 30 (zinc transporter), member 7 (SLC30A7), transcript variant 2, mRNA. \| -1,57 \| \| EZH1 \| Homo sapiens enhancer of zeste 1 polycomb repressive complex 2 subunit (EZH1), mRNA. \| -1,57 \| \| TRPC4AP \| Homo sapiens transient receptor potential cation channel, subfamily C, member 4 associated protein (TRPC4AP), transcript variant 1, mRNA. \| -1,56 \| \| RIOK3 \| Homo sapiens RIO kinase 3 (RIOK3), mRNA. \| -1,56 \| \| LCMT1 \| Homo sapiens leucine carboxyl methyltransferase 1 (LCMT1), transcript variant 2, mRNA. \| -1,56 \| \| H2AFY2 \| Homo sapiens H2A histone family, member Y2 (H2AFY2), mRNA. \| -1,56 \| \| EXOC6B \| Homo sapiens exocyst complex component 6B (EXOC6B), mRNA. \| -1,56 \| \| GNB4 \| Homo sapiens guanine nucleotide binding protein (G protein), beta polypeptide 4 (GNB4), mRNA. \| -1,56 \| \| FNDC1 \| Homo sapiens fibronectin type III domain containing 1 (FNDC1), mRNA. \| -1,56 \| \| SMG1P3 \| Homo sapiens SMG1 pseudogene 3 (SMG1P3), non-coding RNA. \| -1,56 \| \| RIN2 \| Homo sapiens Ras and Rab interactor 2 (RIN2), transcript variant 1, mRNA. \| -1,56 \| \| RCOR1 \| Homo sapiens REST corepressor 1 (RCOR1), mRNA. \| -1,56 \| \| SNORD116-14 \| Homo sapiens small nucleolar RNA, C/D box 116-14 (SNORD116-14), small nucleolar RNA. \| -1,56 \| \| FAM111B \| Homo sapiens family with sequence similarity 111, member B (FAM111B), transcript variant 2, mRNA. \| -1,56 \| \| TM4SF1 \| Homo sapiens transmembrane 4 L six family member 1 (TM4SF1), mRNA. \| -1,56 \| \| WEE1 \| Homo sapiens WEE1 G2 checkpoint kinase (WEE1), transcript variant 2, mRNA. \| -1,56 \| \| CENPN \| Homo sapiens centromere protein N (CENPN), transcript variant 2, mRNA. \| -1,56 \| \| SNAR-I \| Homo sapiens small ILF3/NF90-associated RNA I (SNAR-I), small nuclear RNA. \| -1,56 \| \| --- \| Homo sapiens RNA binding protein S1, serine-rich domain (RNPS1), transcript variant 3, mRNA. \| -1,56 \| \| GTF2IP1 \| Homo sapiens general transcription factor IIi pseudogene 1 (GTF2IP1), non-coding RNA. \| -1,56 \| \| --- \| Homo sapiens SMG1 pseudogene 1 (SMG1P1), non-coding RNA. \| -1,56 \| \| PEAK1 \| Homo sapiens pseudopodium-enriched atypical kinase 1 (PEAK1), mRNA. \| -1,56 \| \| MED13 \| Homo sapiens mediator complex subunit 13 (MED13), mRNA. \| -1,56 \| \| --- \| Homo sapiens phosphodiesterase 4D interacting protein (PDE4DIP), transcript variant 8, mRNA. \| -1,55 \| \| ZBTB41 \| Homo sapiens zinc finger and BTB domain containing 41 (ZBTB41), mRNA. \| -1,55 \| \| --- \| Homo sapiens flotillin 1 (FLOT1), mRNA. \| -1,55 \| \| PDE12 \| Homo sapiens phosphodiesterase 12 (PDE12), mRNA. \| -1,55 \| \| EPDR1 \| Homo sapiens ependymin related 1 (EPDR1), transcript variant 2, mRNA. \| -1,55 \| \| --- \| Non-coding transcript identified by NONCODE: Linc \| -1,55 \| \| --- \| linc-AMELY-16 chrY:-:10035731-10036602 \| -1,55 \| \| ASAP2 \| Homo sapiens ArfGAP with SH3 domain, ankyrin repeat and PH domain 2 (ASAP2), transcript variant 2, mRNA. \| -1,55 \| \| LOC400043 \| Homo sapiens uncharacterized LOC400043 (LOC400043), long non-coding RNA. \| -1,55 \| \| TUBB6 \| Homo sapiens tubulin, beta 6 class V (TUBB6), transcript variant 2, mRNA. \| -1,55 \| \| VPS36 \| Homo sapiens vacuolar protein sorting 36 homolog (S. cerevisiae) (VPS36), transcript variant 2, mRNA. \| -1,55 \| \| SNRNP27 \| Homo sapiens small nuclear ribonucleoprotein 27kDa (U4/U6.U5) (SNRNP27), transcript variant 1, mRNA. \| -1,55 \| \| PIGN \| Homo sapiens phosphatidylinositol glycan anchor biosynthesis, class N (PIGN), transcript variant 2, mRNA. \| -1,55 \| \| PI4KAP2 \| Homo sapiens phosphatidylinositol 4-kinase, catalytic, alpha pseudogene 2 (PI4KAP2), non-coding RNA. \| -1,55 \| \| COL12A1 \| Homo sapiens collagen, type XII, alpha 1 (COL12A1), transcript variant long, mRNA. \| -1,55 \| \| DYNLL2 \| Homo sapiens dynein, light chain, LC8-type 2 (DYNLL2), mRNA. \| -1,55 \| \| RNLS \| Homo sapiens renalase, FAD-dependent amine oxidase (RNLS), transcript variant 1, mRNA. \| -1,55 \| \| ATG3 \| Homo sapiens autophagy related 3 (ATG3), transcript variant 2, mRNA. \| -1,55 \| \| MFN2 \| Homo sapiens mitofusin 2 (MFN2), transcript variant 2, mRNA. \| -1,55 \| \| MIR3654 \| Homo sapiens microRNA 3654 (MIR3654), microRNA. \| -1,55 \| \| --- \| havana:known chromosome:GRCh38:10:17233325:17234833:-1 gene:ENSG00000234961 gene_biotype:antisense transcript_biotype:antisense \| -1,55 \| \| EGFR \| Homo sapiens epidermal growth factor receptor (EGFR), transcript variant 1, mRNA. \| -1,55 \| \| MFSD14B \| Homo sapiens hippocampus abundant transcript-like 1 (HIATL1), mRNA. \| -1,55 \| \| UBL3 \| Homo sapiens ubiquitin-like 3 (UBL3), mRNA. \| -1,55 \| \| --- \| U1 spliceosomal RNA [gene_biotype:snRNA transcript_biotype:snRNA] \| -1,55 \| \| SNORD116-3 \| Homo sapiens small nucleolar RNA, C/D box 116-3 (SNORD116-3), small nucleolar RNA. \| -1,55 \| \| SNORD116-3 \| Homo sapiens small nucleolar RNA, C/D box 116-3 (SNORD116-3), small nucleolar RNA. \| -1,55 \| \| SH3BP4 \| Homo sapiens SH3-domain binding protein 4 (SH3BP4), mRNA. \| -1,54 \| \| PIEZO1 \| Homo sapiens piezo-type mechanosensitive ion channel component 1 (PIEZO1), mRNA. \| -1,54 \| \| SAT1 \| Homo sapiens spermidine/spermine N1-acetyltransferase 1 (SAT1), transcript variant 1, mRNA. \| -1,54 \| \| RSF1 \| Homo sapiens remodeling and spacing factor 1 (RSF1), mRNA. \| -1,54 \| \| --- \| ensembl_lincrna:novel chromosome:GRCh38:18:32412182:32413236:1 gene:ENSG00000262477 gene_biotype:lincRNA transcript_biotype:lincRNA \| -1,54 \| \| GPC6 \| Homo sapiens glypican 6 (GPC6), mRNA. \| -1,54 \| \| SMC5 \| Homo sapiens structural maintenance of chromosomes 5 (SMC5), mRNA. \| -1,54 \| \| STXBP5 \| Homo sapiens syntaxin binding protein 5 (tomosyn) (STXBP5), transcript variant 2, mRNA. \| -1,54 \| \| KIF23 \| Homo sapiens kinesin family member 23 (KIF23), transcript variant 3, mRNA. \| -1,54 \| \| HIST1H3I \| Homo sapiens histone cluster 1, H3i (HIST1H3I), mRNA. \| -1,54 \| \| DIRC2 \| Homo sapiens disrupted in renal carcinoma 2 (DIRC2), mRNA. \| -1,54 \| \| NPC1 \| Homo sapiens Niemann-Pick disease, type C1 (NPC1), mRNA. \| -1,54 \| \| OSTM1 \| Homo sapiens osteopetrosis associated transmembrane protein 1 (OSTM1), mRNA. \| -1,54 \| \| --- \| hect domain and RLD 2 pseudogene 8[gene_biotype:transcribed_unprocessed_pseudogene transcript_biotype:transcribed_unprocessed_pseudogene] \| -1,54 \| \| SGOL1 \| Homo sapiens shugoshin-like 1 (S. pombe) (SGOL1), transcript variant A1, mRNA. \| -1,54 \| \| PRRT3-AS1 \| Homo sapiens PRRT3 antisense RNA 1 (PRRT3-AS1), long non-coding RNA. \| -1,54 \| \| FCHSD2 \| Homo sapiens FCH and double SH3 domains 2 (FCHSD2), mRNA. \| -1,53 \| \| AMMECR1L \| Homo sapiens AMMECR1-like (AMMECR1L), transcript variant 2, mRNA. \| -1,53 \| \| SNORD3C \| Homo sapiens small nucleolar RNA, C/D box 3C (SNORD3C), small nucleolar RNA. \| -1,53 \| \| ZNF253 \| Homo sapiens zinc finger protein 253 (ZNF253), mRNA. \| -1,53 \| \| SNAPC1 \| Homo sapiens small nuclear RNA activating complex, polypeptide 1, 43kDa (SNAPC1), mRNA. \| -1,53 \| \| SNORD10 \| Homo sapiens small nucleolar RNA, C/D box 10 (SNORD10), small nucleolar RNA. \| -1,53 \| \| XPO4 \| Homo sapiens exportin 4 (XPO4), mRNA. \| -1,53 \| \| FCF1 \| Homo sapiens FCF1 rRNA-processing protein (FCF1), mRNA. \| -1,53 \| \| RBMX \| Homo sapiens RNA binding motif protein, X-linked (RBMX), transcript variant 2, mRNA. \| -1,53 \| \| --- \| linc-KRTAP9-1 chr17:+:39264469-39272256 \| -1,53 \| \| DPP9 \| Homo sapiens dipeptidyl-peptidase 9 (DPP9), mRNA. \| -1,53 \| \| LMBRD1 \| Homo sapiens LMBR1 domain containing 1 (LMBRD1), mRNA. \| -1,53 \| \| RAD23B \| Homo sapiens RAD23 homolog B, nucleotide excision repair protein (RAD23B), transcript variant 2, mRNA. \| -1,53 \| \| TNKS2 \| Homo sapiens tankyrase, TRF1-interacting ankyrin-related ADP-ribose polymerase 2 (TNKS2), mRNA. \| -1,53 \| \| ARMC9 \| Homo sapiens armadillo repeat containing 9 (ARMC9), transcript variant 1, mRNA. \| -1,53 \| \| TRMT6 \| Homo sapiens tRNA methyltransferase 6 (TRMT6), transcript variant 2, mRNA. \| -1,53 \| \| EP300 \| Homo sapiens E1A binding protein p300 (EP300), mRNA. \| -1,53 \| \| C9orf78 \| Homo sapiens chromosome 9 open reading frame 78 (C9orf78), mRNA. \| -1,53 \| \| --- \| Homo sapiens flotillin 1 (FLOT1), mRNA. \| -1,53 \| \| --- \| Homo sapiens WW domain binding protein 1 (WBP1), mRNA. \| -1,53 \| \| CHMP2B \| Homo sapiens charged multivesicular body protein 2B (CHMP2B), transcript variant 2, mRNA. \| -1,53 \| \| --- \| Homo sapiens small nucleolar RNA, C/D box 56 (SNORD56), small nucleolar RNA. \| -1,53 \| \| KARS \| Homo sapiens lysyl-tRNA synthetase (KARS), transcript variant 1, mRNA. \| -1,53 \| \| LGR4 \| Homo sapiens leucine-rich repeat containing G protein-coupled receptor 4 (LGR4), mRNA. \| -1,53 \| \| MSRB2 \| Homo sapiens methionine sulfoxide reductase B2 (MSRB2), mRNA. \| -1,53 \| \| RAPGEF2 \| Homo sapiens Rap guanine nucleotide exchange factor (GEF) 2 (RAPGEF2), mRNA. \| -1,53 \| \| TRIM25 \| Homo sapiens tripartite motif containing 25 (TRIM25), mRNA. \| -1,53 \| \| SOS1 \| Homo sapiens son of sevenless homolog 1 (Drosophila) (SOS1), mRNA. \| -1,52 \| \| FEM1C \| Homo sapiens fem-1 homolog c (C. elegans) (FEM1C), mRNA. \| -1,52 \| \| SNORD3D \| Homo sapiens small nucleolar RNA, C/D box 3D (SNORD3D), small nucleolar RNA. \| -1,52 \| \| LOC105373192 \| PREDICTED: Homo sapiens uncharacterized LOC105373192 (LOC105373192), transcript variant X1, ncRNA. \| -1,52 \| \| ATAD2 \| Homo sapiens ATPase family, AAA domain containing 2 (ATAD2), mRNA. \| -1,52 \| \| --- \| SH3 domain containing 19 [gene_biotype:protein_coding transcript_biotype:processed_transcript] \| -1,52 \| \| NR2F2 \| Homo sapiens nuclear receptor subfamily 2, group F, member 2 (NR2F2), transcript variant 2, mRNA. \| -1,52 \| \| RPS19 \| Homo sapiens ribosomal protein S19 (RPS19), mRNA. \| -1,52 \| \| LRRC59 \| Homo sapiens leucine rich repeat containing 59 (LRRC59), mRNA. \| -1,52 \| \| --- \| Homo sapiens CDKN2A interacting protein N-terminal like (CDKN2AIPNL), mRNA. \| -1,52 \| \| SKI \| Homo sapiens v-ski avian sarcoma viral oncogene homolog (SKI), mRNA. \| -1,52 \| \| --- \| Non-coding transcript identified by NONCODE \| -1,52 \| \| USP33 \| Homo sapiens ubiquitin specific peptidase 33 (USP33), transcript variant 1, mRNA. \| -1,52 \| \| REST \| Homo sapiens RE1-silencing transcription factor (REST), transcript variant 2, mRNA. \| -1,52 \| \| CALU \| Homo sapiens calumenin (CALU), transcript variant 2, mRNA. \| -1,52 \| \| NUFIP2 \| Homo sapiens nuclear fragile X mental retardation protein interacting protein 2 (NUFIP2), mRNA. \| -1,52 \| \| FRG1BP \| Homo sapiens FSHD region gene 1 family member B, pseudogene (FRG1BP), non-coding RNA. \| -1,52 \| \| NPIPB5 \| Homo sapiens nuclear pore complex interacting protein family, member B5 (NPIPB5), mRNA. \| -1,52 \| \| CPSF2 \| Homo sapiens cleavage and polyadenylation specific factor 2, 100kDa (CPSF2), mRNA. \| -1,52 \| \| --- \| Homo sapiens major histocompatibility complex, class II, DR alpha (HLA-DRA), mRNA. \| -1,52 \| \| MAP3K3 \| Homo sapiens mitogen-activated protein kinase kinase kinase 3 (MAP3K3), transcript variant 2, mRNA. \| -1,52 \| \| PLK1 \| Homo sapiens polo-like kinase 1 (PLK1), mRNA. \| -1,52 \| \| GTF2H2B \| Homo sapiens general transcription factor IIH, polypeptide 2B (pseudogene) (GTF2H2B), non-coding RNA. \| -1,52 \| \| ATXN7L3B \| Homo sapiens ataxin 7-like 3B (ATXN7L3B), mRNA. \| -1,52 \| \| CDKN3 \| Homo sapiens cyclin-dependent kinase inhibitor 3 (CDKN3), transcript variant 2, mRNA. \| -1,52 \| \| --- \| Homo sapiens dimethylarginine dimethylaminohydrolase 2 (DDAH2), transcript variant 1, mRNA. \| -1,52 \| \| FAM219A \| Homo sapiens family with sequence similarity 219, member A (FAM219A), transcript variant 1, mRNA. \| -1,52 \| \| PLCB4 \| Homo sapiens phospholipase C, beta 4 (PLCB4), transcript variant 1, mRNA. \| -1,52 \| \| LOC105370960 \| PREDICTED: Homo sapiens uncharacterized LOC105370960 (LOC105370960), transcript variant X1, ncRNA. \| -1,52 \| \| --- \| havana:known chromosome:GRCh38:22:32039490:32039896:1 gene:ENSG00000232346 gene_biotype:processed_pseudogene transcript_biotype:processed_pseudogene \| -1,52 \| \| WDHD1 \| Homo sapiens WD repeat and HMG-box DNA binding protein 1 (WDHD1), transcript variant 2, mRNA. \| -1,52 \| \| PDE1C \| Homo sapiens phosphodiesterase 1C, calmodulin-dependent 70kDa (PDE1C), transcript variant 1, mRNA. \| -1,52 \| \| KIF18A \| Homo sapiens kinesin family member 18A (KIF18A), mRNA. \| -1,52 \| \| SNX6 \| Homo sapiens sorting nexin 6 (SNX6), transcript variant 1, mRNA. \| -1,51 \| \| STOM \| Homo sapiens stomatin (STOM), transcript variant 3, mRNA. \| -1,51 \| \| --- \| havana:known chromosome:GRCh38:CHR_HSCHR17_1_CTG5:45835461:45836529:1 gene:ENSG00000281642 gene_biotype:processed_pseudogene transcript_biotype:processed_pseudogene \| -1,51 \| \| --- \| havana:known chromosome:GRCh38:CHR_HSCHR17_1_CTG5:46784842:46785913:-1 gene:ENSG00000281922 gene_biotype:processed_pseudogene transcript_biotype:processed_pseudogene \| -1,51 \| \| NT5E \| Homo sapiens 5-nucleotidase, ecto (CD73) (NT5E), transcript variant 2, mRNA. \| -1,51 \| \| PODXL \| Homo sapiens podocalyxin-like (PODXL), transcript variant 1, mRNA. \| -1,51 \| \| --- \| Homo sapiens centrosomal protein 170kDa (CEP170), transcript variant beta, mRNA. \| -1,51 \| \| TAF1D \| TATA box binding protein (TBP)-associated factor, RNA polymerase I, D, 41kDa [gene_biotype:protein_coding transcript_biotype:retained_intron] \| -1,51 \| \| ZNF326 \| Homo sapiens zinc finger protein 326 (ZNF326), transcript variant 3, mRNA. \| -1,51 \| \| KATNAL1 \| Homo sapiens katanin p60 subunit A-like 1 (KATNAL1), transcript variant 2, mRNA. \| -1,51 \| \| GAS6 \| Homo sapiens growth arrest-specific 6 (GAS6), mRNA. \| -1,51 \| \| --- \| RNA, 5S ribosomal pseudogene 125 [gene_biotype:rRNA transcript_biotype:rRNA] \| -1,51 \| \| PURB \| Homo sapiens purine-rich element binding protein B (PURB), mRNA. \| -1,51 \| \| --- \| Homo sapiens ribonuclease P/MRP 21kDa subunit (RPP21), transcript variant 1, mRNA. \| -1,51 \| \| --- \| Homo sapiens ribonuclease P/MRP 21kDa subunit (RPP21), transcript variant 1, mRNA. \| -1,51 \| \| TRIM39-RPP21 \| Homo sapiens TRIM39-RPP21 readthrough (TRIM39-RPP21), mRNA. \| -1,51 \| \| THOC1 \| Homo sapiens THO complex 1 (THOC1), mRNA. \| -1,51 \| \| BIRC2 \| Homo sapiens baculoviral IAP repeat containing 2 (BIRC2), transcript variant 1, mRNA. \| -1,51 \| \| FAM188A \| Homo sapiens family with sequence similarity 188, member A (FAM188A), mRNA. \| -1,51 \| \| GCLM \| Homo sapiens glutamate-cysteine ligase, modifier subunit (GCLM), transcript variant 2, mRNA. \| -1,51 \| \| LRRC40 \| Homo sapiens leucine rich repeat containing 40 (LRRC40), mRNA. \| -1,51 \| \| --- \| Homo sapiens interferon induced transmembrane protein 4 pseudogene (IFITM4P), non-coding RNA. \| -1,51 \| \| ATP2B1 \| Homo sapiens ATPase, Ca++ transporting, plasma membrane 1 (ATP2B1), transcript variant 1, mRNA. \| -1,51 \| \| VASN \| Homo sapiens vasorin (VASN), mRNA. \| -1,51 \| \| PPIP5K1 \| SubName: Full=Inositol hexakisphosphate and diphosphoinositol-pentakisphosphate kinase 1; \| -1,51 \| \| TSPAN14 \| Homo sapiens tetraspanin 14 (TSPAN14), transcript variant 2, mRNA. \| -1,51 \| \| CYB5A \| Homo sapiens cytochrome b5 type A (microsomal) (CYB5A), transcript variant 3, mRNA. \| -1,51 \| \| SFT2D2 \| Homo sapiens SFT2 domain containing 2 (SFT2D2), mRNA. \| -1,51 \| \| HIST1H4J \| Homo sapiens histone cluster 1, H4j (HIST1H4J), mRNA. \| -1,51 \| \| --- \| Homo sapiens dimethylarginine dimethylaminohydrolase 2 (DDAH2), transcript variant 1, mRNA. \| -1,51 \| \| --- \| Homo sapiens dimethylarginine dimethylaminohydrolase 2 (DDAH2), transcript variant 1, mRNA. \| -1,51 \| \| --- \| Homo sapiens dimethylarginine dimethylaminohydrolase 2 (DDAH2), transcript variant 1, mRNA. \| -1,51 \| \| --- \| Homo sapiens dimethylarginine dimethylaminohydrolase 2 (DDAH2), transcript variant 1, mRNA. \| -1,51 \| \| EIF4A2 \| Homo sapiens eukaryotic translation initiation factor 4A2 (EIF4A2), mRNA. \| -1,51 \| \| CAMSAP2 \| Homo sapiens calmodulin regulated spectrin-associated protein family, member 2 (CAMSAP2), transcript variant 1, mRNA. \| -1,51 \| \| HMMR \| Homo sapiens hyaluronan-mediated motility receptor (RHAMM) (HMMR), transcript variant 1, mRNA. \| -1,50 \| \| HIST2H2BB \| histone cluster 2, H2bb (pseudogene)[gene_biotype:transcribed_unprocessed_pseudogene transcript_biotype:transcribed_unprocessed_pseudogene] \| -1,50 \| \| --- \| ncrna:novel chromosome:GRCh38:12:103985111:103985204:1 gene:ENSG00000215976 gene_biotype:miRNA transcript_biotype:miRNA \| -1,50 \| \| FN3KRP \| Homo sapiens fructosamine 3 kinase related protein (FN3KRP), transcript variant 1, mRNA. \| -1,50 \| \| --- \| havana:known chromosome:GRCh38:7:56809214:56848800:-1 gene:ENSG00000279072 gene_biotype:lincRNA transcript_biotype:lincRNA \| -1,50 \| \| --- \| havana:known chromosome:GRCh38:X:44741087:44741964:1 gene:ENSG00000214016 gene_biotype:processed_pseudogene transcript_biotype:processed_pseudogene \| -1,50 \| \| --- \| Homo sapiens small nucleolar RNA, C/D box 36A (SNORD36A), small nucleolar RNA. \| -1,50 \| \| EFTUD2 \| Homo sapiens elongation factor Tu GTP binding domain containing 2 (EFTUD2), transcript variant 2, mRNA. \| -1,50 \| \| TM7SF3 \| Homo sapiens transmembrane 7 superfamily member 3 (TM7SF3), mRNA. \| -1,50 \| \| MAP4K3 \| Homo sapiens mitogen-activated protein kinase kinase kinase kinase 3 (MAP4K3), transcript variant 2, mRNA. \| -1,50 \| \| ANKRD40 \| Homo sapiens ankyrin repeat domain 40 (ANKRD40), mRNA. \| -1,50 \| \| RSRP1 \| Homo sapiens arginine/serine-rich protein 1 (RSRP1), mRNA. \| -1,50 \| \| ZNF703 \| Homo sapiens zinc finger protein 703 (ZNF703), mRNA. \| -1,50 \| \| USP9X \| Homo sapiens ubiquitin specific peptidase 9, X-linked (USP9X), transcript variant 3, mRNA. \| -1,50 \| \| MAVS \| Homo sapiens mitochondrial antiviral signaling protein (MAVS), transcript variant 3, mRNA. \| -1,50 \| \| BUB3 \| Homo sapiens BUB3 mitotic checkpoint protein (BUB3), transcript variant 2, mRNA. \| -1,50 \| \| UBR2 \| Homo sapiens ubiquitin protein ligase E3 component n-recognin 2 (UBR2), transcript variant 2, mRNA. \| -1,50 \|  \| **anti-miR-149** \| \| \| \| --- \| --- \| --- \| \| **Gene Symbol** \| **mRna - Description** \| **Fold Change** \| \| CST2 \| Homo sapiens cystatin SA (CST2), mRNA. \| 2,87 \| \| --- \| linc-KRTAP9-1 chr17:+:39264469-39271423 \| 2,83 \| \| ABLIM1 \| PREDICTED: Homo sapiens actin binding LIM protein 1 (ABLIM1), transcript variant X8, mRNA. \| 2,49 \| \| --- \| linc-KRTAP9-1 chr17:+:39264469-39272256 \| 2,46 \| \| --- \| Homo sapiens B-cell receptor-associated protein 31 (BCAP31), transcript variant 3, mRNA. \| 2,42 \| \| --- \| RNA, U1 small nuclear 83, pseudogene [gene_biotype:snRNA transcript_biotype:snRNA] \| 2,31 \| \| NPTX1 \| Homo sapiens neuronal pentraxin I (NPTX1), mRNA. \| 2,26 \| \| SNORD114-3 \| Homo sapiens small nucleolar RNA, C/D box 114-3 (SNORD114-3), small nucleolar RNA. \| 2,22 \| \| EPGN \| Homo sapiens epithelial mitogen (EPGN), transcript variant 1, mRNA. \| 2,18 \| \| BEX1 \| Homo sapiens brain expressed, X-linked 1 (BEX1), mRNA. \| 2,06 \| \| PTGS1 \| Homo sapiens prostaglandin-endoperoxide synthase 1 (prostaglandin G/H synthase and cyclooxygenase) (PTGS1), transcript variant 1, mRNA. \| 2,00 \| \| SLC16A6 \| Homo sapiens solute carrier family 16, member 6 (SLC16A6), transcript variant 1, mRNA. \| 1,99 \| \| FOS \| Homo sapiens FBJ murine osteosarcoma viral oncogene homolog (FOS), mRNA. \| 1,97 \| \| UBE2M \| Homo sapiens ubiquitin-conjugating enzyme E2M (UBE2M), mRNA. \| 1,94 \| \| RNVU1-14 \| Homo sapiens RNA, variant U1 small nuclear 14 (RNVU1-14), small nuclear RNA. \| 1,93 \| \| SCAF1 \| Homo sapiens SR-related CTD-associated factor 1 (SCAF1), mRNA. \| 1,93 \| \| MEDAG \| Homo sapiens mesenteric estrogen-dependent adipogenesis (MEDAG), mRNA. \| 1,88 \| \| --- \| RNA, U6 small nuclear 135, pseudogene [gene_biotype:snRNA transcript_biotype:snRNA] \| 1,86 \| \| --- \| mitochondrially encoded tRNA asparagine [gene_biotype:Mt_tRNA transcript_biotype:Mt_tRNA] \| 1,85 \| \| --- \| linc-KRTAP9-1 chr17:+:39264469-39272256 \| 1,85 \| \| RELN \| Homo sapiens reelin (RELN), transcript variant 1, mRNA. \| 1,84 \| \| RRN3 \| Homo sapiens RRN3 homolog, RNA polymerase I transcription factor (RRN3), transcript variant 2, mRNA. \| 1,84 \| \| PALMD \| Homo sapiens palmdelphin (PALMD), mRNA. \| 1,84 \| \| --- \| ncrna:novel scaffold:GRCh38:GL000224.1:166714:166794:1 gene:ENSG00000275189 gene_biotype:miRNA transcript_biotype:miRNA \| 1,82 \| \| SNORA71D \| Homo sapiens small nucleolar RNA, H/ACA box 71D (SNORA71D), small nucleolar RNA. \| 1,81 \| \| CSGALNACT2 \| Homo sapiens chondroitin sulfate N-acetylgalactosaminyltransferase 2 (CSGALNACT2), mRNA. \| 1,80 \| \| --- \| Homo sapiens protein kinase, X-linked, pseudogene 1 (PRKXP1), non-coding RNA. \| 1,79 \| \| SNORD59A \| Homo sapiens small nucleolar RNA, C/D box 59A (SNORD59A), small nucleolar RNA. \| 1,79 \| \| FAM43A \| Homo sapiens family with sequence similarity 43, member A (FAM43A), mRNA. \| 1,78 \| \| SNORD88C \| Homo sapiens small nucleolar RNA, C/D box 88C (SNORD88C), small nucleolar RNA. \| 1,78 \| \| MIR1538 \| Homo sapiens microRNA 1538 (MIR1538), microRNA. \| 1,77 \| \| FAM215A \| Homo sapiens family with sequence similarity 215, member A (non-protein coding) (FAM215A), long non-coding RNA. \| 1,77 \| \| MIR4316 \| Homo sapiens microRNA 4316 (MIR4316), microRNA. \| 1,77 \| \| --- \| PREDICTED: Homo sapiens uncharacterized LOC101928451 (LOC101928451), transcript variant X1, misc_RNA. \| 1,76 \| \| ANGPTL2 \| Homo sapiens angiopoietin-like 2 (ANGPTL2), mRNA. \| 1,76 \| \| SLC25A3 \| solute carrier family 25 (mitochondrial carrier; phosphate carrier), member 3 [gene_biotype:protein_coding transcript_biotype:protein_coding] \| 1,76 \| \| --- \| solute carrier family 25 (mitochondrial carrier; phosphate carrier), member 3 [gene_biotype:protein_coding transcript_biotype:protein_coding] \| 1,76 \| \| --- \| RNA, 5S ribosomal pseudogene 151 [gene_biotype:rRNA transcript_biotype:rRNA] \| 1,75 \| \| THBD \| Homo sapiens thrombomodulin (THBD), mRNA. \| 1,75 \| \| NLRP1 \| NLR family, pyrin domain containing 1 [gene_biotype:protein_coding transcript_biotype:nonsense_mediated_decay] \| 1,75 \| \| PAPPA \| Homo sapiens pregnancy-associated plasma protein A, pappalysin 1 (PAPPA), mRNA. \| 1,74 \| \| TNFRSF10D \| Homo sapiens tumor necrosis factor receptor superfamily, member 10d, decoy with truncated death domain (TNFRSF10D), mRNA. \| 1,74 \| \| OLFM2 \| Homo sapiens olfactomedin 2 (OLFM2), transcript variant 1, mRNA. \| 1,74 \| \| SRPX2 \| Homo sapiens sushi-repeat containing protein, X-linked 2 (SRPX2), mRNA. \| 1,73 \| \| ZBED6 \| Homo sapiens zinc finger, BED-type containing 6 (ZBED6), mRNA. \| 1,73 \| \| SNX9 \| Homo sapiens sorting nexin 9 (SNX9), mRNA. \| 1,72 \| \| --- \| RNA, U6 small nuclear 237, pseudogene [gene_biotype:snRNA transcript_biotype:snRNA] \| 1,72 \| \| ARL8A \| Homo sapiens ADP-ribosylation factor-like 8A (ARL8A), transcript variant 2, mRNA. \| 1,72 \| \| EBF2 \| Homo sapiens early B-cell factor 2 (EBF2), mRNA. \| 1,72 \| \| CCL2 \| Homo sapiens chemokine (C-C motif) ligand 2 (CCL2), mRNA. \| 1,71 \| \| ADRA2C \| Homo sapiens adrenoceptor alpha 2C (ADRA2C), mRNA. \| 1,71 \| \| TOB1 \| Homo sapiens transducer of ERBB2, 1 (TOB1), transcript variant 2, mRNA. \| 1,71 \| \| BMPR1A \| Homo sapiens bone morphogenetic protein receptor, type IA (BMPR1A), mRNA. \| 1,71 \| \| --- \| PREDICTED: Homo sapiens uncharacterized LOC101928451 (LOC101928451), transcript variant X1, misc_RNA. \| 1,69 \| \| SECTM1 \| Homo sapiens secreted and transmembrane 1 (SECTM1), mRNA. \| 1,69 \| \| MIR548X2 \| Homo sapiens microRNA 548x-2 (MIR548X2), microRNA. \| 1,69 \| \| HSPB6 \| Homo sapiens heat shock protein, alpha-crystallin-related, B6 (HSPB6), mRNA. \| 1,68 \| \| RPP14 \| Homo sapiens ribonuclease P/MRP 14kDa subunit (RPP14), transcript variant 1, mRNA. \| 1,68 \| \| --- \| ncrna:novel chromosome:GRCh38:9:64809342:64809423:-1 gene:ENSG00000239028 gene_biotype:miRNA transcript_biotype:miRNA \| 1,68 \| \| --- \| SH3 domain containing 19 [gene_biotype:protein_coding transcript_biotype:processed_transcript] \| 1,67 \| \| AJAP1 \| Homo sapiens adherens junctions associated protein 1 (AJAP1), transcript variant 2, mRNA. \| 1,67 \| \| PRG4 \| Homo sapiens proteoglycan 4 (PRG4), transcript variant B, mRNA. \| 1,66 \| \| --- \| RNA, 5S ribosomal pseudogene 496 [gene_biotype:rRNA transcript_biotype:rRNA] \| 1,65 \| \| --- \| havana:known chromosome:GRCh38:2:219425071:219426184:-1 gene:ENSG00000234638 gene_biotype:antisense transcript_biotype:antisense \| 1,65 \| \| --- \| Homo sapiens small nucleolar RNA, H/ACA box 45A (SNORA45A), small nucleolar RNA. \| 1,65 \| \| F2R \| Homo sapiens coagulation factor II (thrombin) receptor (F2R), transcript variant 2, mRNA. \| 1,65 \| \| --- \| Homo sapiens WW domain binding protein 1 (WBP1), mRNA. \| 1,65 \| \| LGMN \| Homo sapiens legumain (LGMN), transcript variant 2, mRNA. \| 1,64 \| \| TRH \| Homo sapiens thyrotropin-releasing hormone (TRH), mRNA. \| 1,64 \| \| --- \| ncrna:novel chromosome:GRCh38:15:90295041:90295123:1 gene:ENSG00000221309 gene_biotype:miRNA transcript_biotype:miRNA \| 1,63 \| \| PLA2G4A \| Homo sapiens phospholipase A2, group IVA (cytosolic, calcium-dependent) (PLA2G4A), transcript variant 2, mRNA. \| 1,63 \| \| LOC105373192 \| PREDICTED: Homo sapiens uncharacterized LOC105373192 (LOC105373192), transcript variant X1, ncRNA. \| 1,63 \| \| --- \| Homo sapiens B-cell receptor-associated protein 31 (BCAP31), transcript variant 3, mRNA. \| 1,63 \| \| LOC105378596 \| PREDICTED: Homo sapiens uncharacterized LOC105378596 (LOC105378596), ncRNA. \| 1,63 \| \| WFDC1 \| Homo sapiens WAP four-disulfide core domain 1 (WFDC1), transcript variant 2, mRNA. \| 1,62 \| \| NBPF19 \| PREDICTED: Homo sapiens neuroblastoma breakpoint family, member 19 (NBPF19), transcript variant X1, mRNA. \| 1,62 \| \| SAT2 \| Homo sapiens spermidine/spermine N1-acetyltransferase family member 2 (SAT2), mRNA. \| 1,62 \| \| MIR21 \| Homo sapiens microRNA 21 (MIR21), microRNA. \| 1,62 \| \| --- \| Non-coding transcript identified by NONCODE \| 1,61 \| \| TAF1D \| TATA box binding protein (TBP)-associated factor, RNA polymerase I, D, 41kDa [gene_biotype:protein_coding transcript_biotype:nonsense_mediated_decay] \| 1,61 \| \| SPAST \| Homo sapiens spastin (SPAST), transcript variant 1, mRNA. \| 1,60 \| \| MEOX2 \| Homo sapiens mesenchyme homeobox 2 (MEOX2), mRNA. \| 1,60 \| \| AKAP12 \| Homo sapiens A kinase (PRKA) anchor protein 12 (AKAP12), transcript variant 1, mRNA. \| 1,60 \| \| ATP6V0A1 \| Homo sapiens ATPase, H+ transporting, lysosomal V0 subunit a1 (ATP6V0A1), transcript variant 1, mRNA. \| 1,60 \| \| EREG \| Homo sapiens epiregulin (EREG), mRNA. \| 1,60 \| \| --- \| small nucleolar RNA, C/D box 45B [gene_biotype:snoRNA transcript_biotype:snoRNA] \| 1,60 \| \| SLC1A1 \| Homo sapiens solute carrier family 1 (neuronal/epithelial high affinity glutamate transporter, system Xag), member 1 (SLC1A1), mRNA. \| 1,60 \| \| SYNPO2 \| Homo sapiens synaptopodin 2 (SYNPO2), transcript variant 2, mRNA. \| 1,60 \| \| NOTCH2NL \| notch 2 N-terminal like [gene_biotype:protein_coding transcript_biotype:processed_transcript] \| 1,60 \| \| --- \| ensembl:known chromosome:GRCh38:5:60430738:60431112:1 gene:ENSG00000280447 gene_biotype:protein_coding transcript_biotype:protein_coding \| 1,59 \| \| GNG11 \| Homo sapiens guanine nucleotide binding protein (G protein), gamma 11 (GNG11), mRNA. \| 1,59 \| \| SCUBE3 \| Homo sapiens signal peptide, CUB domain, EGF-like 3 (SCUBE3), transcript variant 2, mRNA. \| 1,59 \| \| ANKRD12 \| Homo sapiens ankyrin repeat domain 12 (ANKRD12), transcript variant 2, mRNA. \| 1,59 \| \| --- \| Homo sapiens fibroblast growth factor 7 (FGF7), mRNA. \| 1,59 \| \| ABCA6 \| Homo sapiens ATP-binding cassette, sub-family A (ABC1), member 6 (ABCA6), mRNA. \| 1,58 \| \| LOC105377763 \| PREDICTED: Homo sapiens uncharacterized LOC105377763 (LOC105377763), transcript variant X1, ncRNA. \| 1,58 \| \| INAFM2 \| Homo sapiens InaF-motif containing 2 (INAFM2), mRNA. \| 1,58 \| \| TMEM254 \| Homo sapiens transmembrane protein 254 (TMEM254), transcript variant 2, mRNA. \| 1,58 \| \| MIR5047 \| Homo sapiens microRNA 5047 (MIR5047), microRNA. \| 1,57 \| \| MIR1229 \| Homo sapiens microRNA 1229 (MIR1229), microRNA. \| 1,57 \| \| --- \| Homo sapiens microRNA 1229 (MIR1229), microRNA. \| 1,57 \| \| --- \| Homo sapiens microRNA 4469 (MIR4469), microRNA. \| 1,57 \| \| STC1 \| Homo sapiens stanniocalcin 1 (STC1), mRNA. \| 1,57 \| \| NSF \| Homo sapiens N-ethylmaleimide-sensitive factor (NSF), transcript variant 1, mRNA. \| 1,57 \| \| COX7A1 \| Homo sapiens cytochrome c oxidase subunit VIIa polypeptide 1 (muscle) (COX7A1), mRNA. \| 1,57 \| \| FAM89B \| Homo sapiens family with sequence similarity 89, member B (FAM89B), transcript variant 3, mRNA. \| 1,57 \| \| HGF \| Homo sapiens hepatocyte growth factor (hepapoietin A; scatter factor) (HGF), transcript variant 1, mRNA. \| 1,57 \| \| TAF13 \| Homo sapiens TAF13 RNA polymerase II, TATA box binding protein (TBP)-associated factor, 18kDa (TAF13), mRNA. \| 1,57 \| \| P2RY8 \| PREDICTED: Homo sapiens purinergic receptor P2Y, G-protein coupled, 8 (P2RY8), transcript variant X3, mRNA. \| 1,56 \| \| FLG \| Homo sapiens filaggrin (FLG), mRNA. \| 1,56 \| \| --- \| Homo sapiens small nucleolar RNA, C/D box 34 (SNORD34), small nucleolar RNA. \| 1,56 \| \| RNU12 \| Homo sapiens RNA, U12 small nuclear (RNU12), small nuclear RNA. \| 1,56 \| \| C16orf45 \| Homo sapiens chromosome 16 open reading frame 45 (C16orf45), transcript variant 2, mRNA. \| 1,56 \| \| --- \| Non-coding transcript identified by NONCODE \| 1,55 \| \| TINAGL1 \| Homo sapiens tubulointerstitial nephritis antigen-like 1 (TINAGL1), transcript variant 2, mRNA. \| 1,55 \| \| EDNRB \| Homo sapiens endothelin receptor type B (EDNRB), transcript variant 1, mRNA. \| 1,55 \| \| LINC00674 \| Homo sapiens long intergenic non-protein coding RNA 674 (LINC00674), long non-coding RNA. \| 1,55 \| \| LOC729739 \| Homo sapiens peptidylprolyl isomerase A (cyclophilin A) pseudogene (LOC729739), non-coding RNA. \| 1,55 \| \| RFWD2 \| Homo sapiens ring finger and WD repeat domain 2, E3 ubiquitin protein ligase (RFWD2), transcript variant 2, mRNA. \| 1,55 \| \| LRRC32 \| Homo sapiens leucine rich repeat containing 32 (LRRC32), transcript variant 2, mRNA. \| 1,55 \| \| --- \| mitochondrially encoded tRNA serine 1 (UCN) [gene_biotype:Mt_tRNA transcript_biotype:Mt_tRNA] \| 1,55 \| \| --- \| Homo sapiens poly (ADP-ribose) polymerase family, member 4 (PARP4), mRNA. \| 1,55 \| \| --- \| Homo sapiens golgin A8 family, member A (GOLGA8A), transcript variant 2, non-coding RNA. \| 1,55 \| \| SAT1 \| Homo sapiens spermidine/spermine N1-acetyltransferase 1 (SAT1), transcript variant 1, mRNA. \| 1,54 \| \| FOSB \| Homo sapiens FBJ murine osteosarcoma viral oncogene homolog B (FOSB), transcript variant 2, mRNA. \| 1,54 \| \| LOC728323 \| Homo sapiens cDNA FLJ60027 complete cds, moderately similar to F-box only protein 25. \| 1,54 \| \| ARPP19 \| Homo sapiens cAMP-regulated phosphoprotein, 19kDa (ARPP19), transcript variant 1, mRNA. \| 1,54 \| \| ZFP90 \| Homo sapiens ZFP90 zinc finger protein (ZFP90), transcript variant 2, mRNA. \| 1,54 \| \| --- \| Homo sapiens ubiquitin specific peptidase 32 (USP32), mRNA. \| 1,54 \| \| MFSD14A \| Homo sapiens hippocampus abundant transcript 1 (HIAT1), mRNA. \| 1,54 \| \| PAMR1 \| Homo sapiens peptidase domain containing associated with muscle regeneration 1 (PAMR1), transcript variant 2, mRNA. \| 1,54 \| \| SNORD37 \| Homo sapiens small nucleolar RNA, C/D box 37 (SNORD37), small nucleolar RNA. \| 1,54 \| \| FBXO17 \| Homo sapiens F-box protein 17 (FBXO17), transcript variant 2, mRNA. \| 1,54 \| \| TRAPPC8 \| Homo sapiens trafficking protein particle complex 8 (TRAPPC8), mRNA. \| 1,53 \| \| --- \| ncrna:novel chromosome:GRCh38:18:7738111:7738225:-1 gene:ENSG00000221631 gene_biotype:miRNA transcript_biotype:miRNA \| 1,53 \| \| ESPNP \| Homo sapiens espin pseudogene (ESPNP), non-coding RNA. \| 1,53 \| \| --- \| Homo sapiens cDNA FLJ10288 fis, clone MAMMA1002317. \| 1,52 \| \| CYGB \| Homo sapiens cytoglobin (CYGB), mRNA. \| 1,52 \| \| --- \| Non-coding transcript identified by NONCODE \| 1,52 \| \| GOLGB1 \| Homo sapiens golgin B1 (GOLGB1), transcript variant 1, mRNA. \| 1,52 \| \| --- \| Homo sapiens chloride channel, voltage-sensitive 3 (CLCN3), transcript variant a, mRNA. \| 1,52 \| \| --- \| ncrna:novel chromosome:GRCh38:15:99792297:99792379:1 gene:ENSG00000221511 gene_biotype:miRNA transcript_biotype:miRNA \| 1,52 \| \| MIR4642 \| Homo sapiens microRNA 4642 (MIR4642), microRNA. \| 1,52 \| \| PI4KB \| Homo sapiens phosphatidylinositol 4-kinase, catalytic, beta (PI4KB), transcript variant 2, mRNA. \| 1,51 \| \| WDR47 \| Homo sapiens WD repeat domain 47 (WDR47), transcript variant 1, mRNA. \| 1,51 \| \| TRPC4 \| Homo sapiens transient receptor potential cation channel, subfamily C, member 4 (TRPC4), transcript variant beta, mRNA. \| 1,51 \| \| LACC1 \| Homo sapiens laccase (multicopper oxidoreductase) domain containing 1 (LACC1), transcript variant 1, mRNA. \| 1,51 \| \| --- \| linc-KRTAP9-1 chr17:+:39264469-39271423 \| 1,51 \| \| DHX33 \| Homo sapiens DEAH (Asp-Glu-Ala-His) box polypeptide 33 (DHX33), transcript variant 2, mRNA. \| 1,51 \| \| F2RL2 \| Homo sapiens coagulation factor II (thrombin) receptor-like 2 (F2RL2), transcript variant 2, mRNA. \| 1,51 \| \| DEXI \| Homo sapiens Dexi homolog (mouse) (DEXI), mRNA. \| 1,51 \| \| --- \| RNA, U1 small nuclear 38, pseudogene [gene_biotype:snRNA transcript_biotype:snRNA] \| 1,51 \| \| --- \| RNA, U1 small nuclear 38, pseudogene [gene_biotype:snRNA transcript_biotype:snRNA] \| 1,51 \| \| --- \| Homo sapiens mitofusin 1 (MFN1), mRNA. \| 1,51 \| \| MMP1 \| Homo sapiens matrix metallopeptidase 1 (MMP1), transcript variant 2, mRNA. \| 1,51 \| \| SCD \| Homo sapiens stearoyl-CoA desaturase (delta-9-desaturase) (SCD), mRNA. \| 1,50 \| \| SLC16A4 \| Homo sapiens solute carrier family 16, member 4 (SLC16A4), transcript variant 2, mRNA. \| 1,50 \| \| BCL2L1 \| Homo sapiens BCL2-like 1 (BCL2L1), transcript variant 2, mRNA. \| 1,50 \| \| PDGFRB \| Homo sapiens platelet-derived growth factor receptor, beta polypeptide (PDGFRB), mRNA. \| 1,50 \|  \| **anti-miR-361** \| \| \| \| --- \| --- \| --- \| \| **Gene Symbol** \| **mRna - Description** \| **Fold Change** \| \| --- \| RNA, U5A small nuclear 8, pseudogene [gene_biotype:snRNA transcript_biotype:snRNA] \| 2,42 \| \| PSAT1 \| Homo sapiens phosphoserine aminotransferase 1 (PSAT1), transcript variant 2, mRNA. \| 2,23 \| \| --- \| mitochondrially encoded tRNA threonine [gene_biotype:Mt_tRNA transcript_biotype:Mt_tRNA] \| 1,98 \| \| --- \| Homo sapiens B-cell receptor-associated protein 31 (BCAP31), transcript variant 3, mRNA. \| 1,94 \| \| ANKRD1 \| Homo sapiens ankyrin repeat domain 1 (cardiac muscle) (ANKRD1), mRNA. \| 1,92 \| \| --- \| mitochondrially encoded tRNA isoleucine [gene_biotype:Mt_tRNA transcript_biotype:Mt_tRNA] \| 1,85 \| \| KRTAP2-3 \| Homo sapiens keratin associated protein 2-3 (KRTAP2-3), mRNA. \| 1,82 \| \| TAF1D \| TATA box binding protein (TBP)-associated factor, RNA polymerase I, D, 41kDa [gene_biotype:protein_coding transcript_biotype:nonsense_mediated_decay] \| 1,81 \| \| PDCD1LG2 \| Homo sapiens programmed cell death 1 ligand 2 (PDCD1LG2), mRNA. \| 1,79 \| \| --- \| Homo sapiens eukaryotic translation initiation factor 3, subunit J (EIF3J), transcript variant 2, mRNA. \| 1,77 \| \| NPIPB11 \| PREDICTED: Homo sapiens nuclear pore complex interacting protein family, member B11 (NPIPB11), transcript variant X1, mRNA. \| 1,75 \| \| SKA3 \| Homo sapiens spindle and kinetochore associated complex subunit 3 (SKA3), transcript variant 2, mRNA. \| 1,75 \| \| CDK17 \| Homo sapiens cyclin-dependent kinase 17 (CDK17), transcript variant 2, mRNA. \| 1,75 \| \| TAF13 \| Homo sapiens TAF13 RNA polymerase II, TATA box binding protein (TBP)-associated factor, 18kDa (TAF13), mRNA. \| 1,71 \| \| --- \| mitochondrially encoded tRNA leucine 2 (CUN) [gene_biotype:Mt_tRNA transcript_biotype:Mt_tRNA] \| 1,71 \| \| HNRNPA1 \| Homo sapiens heterogeneous nuclear ribonucleoprotein A1 (HNRNPA1), transcript variant 2, mRNA. \| 1,71 \| \| LRRC15 \| Homo sapiens leucine rich repeat containing 15 (LRRC15), transcript variant 1, mRNA. \| 1,70 \| \| DKK1 \| Homo sapiens dickkopf WNT signaling pathway inhibitor 1 (DKK1), mRNA. \| 1,70 \| \| MIR1229 \| Homo sapiens microRNA 1229 (MIR1229), microRNA. \| 1,70 \| \| --- \| Homo sapiens microRNA 1229 (MIR1229), microRNA. \| 1,70 \| \| ARPP19 \| Homo sapiens cAMP-regulated phosphoprotein, 19kDa (ARPP19), transcript variant 1, mRNA. \| 1,69 \| \| --- \| mitochondrially encoded tRNA asparagine [gene_biotype:Mt_tRNA transcript_biotype:Mt_tRNA] \| 1,69 \| \| MBOAT2 \| Homo sapiens membrane bound O-acyltransferase domain containing 2 (MBOAT2), mRNA. \| 1,63 \| \| ESM1 \| Homo sapiens endothelial cell-specific molecule 1 (ESM1), transcript variant 2, mRNA. \| 1,62 \| \| FNDC1 \| Homo sapiens fibronectin type III domain containing 1 (FNDC1), mRNA. \| 1,62 \| \| SNX4 \| Homo sapiens sorting nexin 4 (SNX4), transcript variant 1, mRNA. \| 1,62 \| \| --- \| RNA, 5S ribosomal pseudogene 113 [gene_biotype:rRNA transcript_biotype:rRNA] \| 1,62 \| \| DDX58 \| Homo sapiens DEAD (Asp-Glu-Ala-Asp) box polypeptide 58 (DDX58), mRNA. \| 1,61 \| \| ARL13B \| Homo sapiens ADP-ribosylation factor-like 13B (ARL13B), transcript variant 3, mRNA. \| 1,61 \| \| CENPI \| Homo sapiens centromere protein I (CENPI), mRNA. \| 1,61 \| \| --- \| 5S ribosomal RNA [gene_biotype:rRNA transcript_biotype:rRNA] \| 1,61 \| \| --- \| Non-coding transcript identified by NONCODE \| 1,60 \| \| --- \| small nucleolar RNA, C/D box 45B [gene_biotype:snoRNA transcript_biotype:snoRNA] \| 1,60 \| \| --- \| PREDICTED: Homo sapiens uncharacterized LOC101928451 (LOC101928451), transcript variant X1, misc_RNA. \| 1,60 \| \| --- \| mitochondrially encoded tRNA arginine [gene_biotype:Mt_tRNA transcript_biotype:Mt_tRNA] \| 1,60 \| \| --- \| Homo sapiens transcription elongation factor B (SIII), polypeptide 1 (15kDa, elongin C) (TCEB1), transcript variant 2, mRNA. \| 1,59 \| \| --- \| Small nucleolar RNA SNORA44 [gene_biotype:snoRNA transcript_biotype:snoRNA] \| 1,59 \| \| NBPF19 \| PREDICTED: Homo sapiens neuroblastoma breakpoint family, member 19 (NBPF19), transcript variant X1, mRNA. \| 1,59 \| \| KRT34 \| Homo sapiens keratin 34, type I (KRT34), mRNA. \| 1,59 \| \| --- \| Homo sapiens SMG1 phosphatidylinositol 3-kinase-related kinase (SMG1), mRNA. \| 1,59 \| \| --- \| RNA, U6 small nuclear 707, pseudogene [gene_biotype:snRNA transcript_biotype:snRNA] \| 1,59 \| \| GPCPD1 \| Homo sapiens glycerophosphocholine phosphodiesterase 1 (GPCPD1), mRNA. \| 1,59 \| \| CKAP2L \| Homo sapiens cytoskeleton associated protein 2-like (CKAP2L), transcript variant 2, mRNA. \| 1,58 \| \| UBE2M \| Homo sapiens ubiquitin-conjugating enzyme E2M (UBE2M), mRNA. \| 1,58 \| \| DSP \| Homo sapiens desmoplakin (DSP), transcript variant 2, mRNA. \| 1,58 \| \| --- \| havana:known chromosome:GRCh38:9:66950394:66952344:1 gene:ENSG00000277350 gene_biotype:lincRNA transcript_biotype:lincRNA \| 1,58 \| \| SLBP \| Homo sapiens stem-loop binding protein (SLBP), transcript variant 2, mRNA. \| 1,58 \| \| --- \| growth arrest-specific 5 (non-protein coding) [gene_biotype:processed_transcript transcript_biotype:retained_intron] \| 1,57 \| \| CCDC18 \| Homo sapiens coiled-coil domain containing 18 (CCDC18), transcript variant 1, mRNA. \| 1,57 \| \| FAM86C2P \| Homo sapiens family with sequence similarity 86, member C2, pseudogene (FAM86C2P), non-coding RNA. \| 1,57 \| \| LOC389831 \| Homo sapiens uncharacterized LOC389831 (LOC389831), mRNA. \| 1,57 \| \| --- \| mitochondrially encoded tRNA tryptophan [gene_biotype:Mt_tRNA transcript_biotype:Mt_tRNA] \| 1,57 \| \| SNORD114-3 \| Homo sapiens small nucleolar RNA, C/D box 114-3 (SNORD114-3), small nucleolar RNA. \| 1,56 \| \| --- \| PREDICTED: Homo sapiens uncharacterized LOC101928451 (LOC101928451), transcript variant X1, misc_RNA. \| 1,56 \| \| WDR44 \| Homo sapiens WD repeat domain 44 (WDR44), transcript variant 2, mRNA. \| 1,56 \| \| CSGALNACT2 \| Homo sapiens chondroitin sulfate N-acetylgalactosaminyltransferase 2 (CSGALNACT2), mRNA. \| 1,56 \| \| --- \| Homo sapiens golgin A8 family, member A (GOLGA8A), transcript variant 2, non-coding RNA. \| 1,56 \| \| --- \| RNA, 5S ribosomal pseudogene 279 [gene_biotype:rRNA transcript_biotype:rRNA] \| 1,56 \| \| --- \| Homo sapiens small nucleolar RNA, C/D box 34 (SNORD34), small nucleolar RNA. \| 1,55 \| \| SNORD3C \| Homo sapiens small nucleolar RNA, C/D box 3C (SNORD3C), small nucleolar RNA. \| 1,55 \| \| LOC105373192 \| PREDICTED: Homo sapiens uncharacterized LOC105373192 (LOC105373192), transcript variant X1, ncRNA. \| 1,55 \| \| WDR43 \| Homo sapiens WD repeat domain 43 (WDR43), mRNA. \| 1,55 \| \| ARMT1 \| Homo sapiens acidic residue methyltransferase 1 (ARMT1), transcript variant 2, mRNA. \| 1,55 \| \| MIR221 \| Homo sapiens microRNA 221 (MIR221), microRNA. \| 1,55 \| \| --- \| Homo sapiens poly (ADP-ribose) polymerase family, member 4 (PARP4), mRNA. \| 1,55 \| \| NRAS \| Homo sapiens neuroblastoma RAS viral (v-ras) oncogene homolog (NRAS), mRNA. \| 1,54 \| \| ENPP1 \| Homo sapiens ectonucleotide pyrophosphatase/phosphodiesterase 1 (ENPP1), mRNA. \| 1,54 \| \| NDC80 \| Homo sapiens NDC80 kinetochore complex component (NDC80), mRNA. \| 1,54 \| \| RRN3 \| Homo sapiens RRN3 homolog, RNA polymerase I transcription factor (RRN3), transcript variant 2, mRNA. \| 1,54 \| \| KIF20B \| Homo sapiens kinesin family member 20B (KIF20B), transcript variant 1, mRNA. \| 1,54 \| \| HMMR \| Homo sapiens hyaluronan-mediated motility receptor (RHAMM) (HMMR), transcript variant 1, mRNA. \| 1,53 \| \| LTV1 \| Homo sapiens LTV1 ribosome biogenesis factor (LTV1), mRNA. \| 1,53 \| \| CDH2 \| Homo sapiens cadherin 2, type 1, N-cadherin (neuronal) (CDH2), transcript variant 2, mRNA. \| 1,53 \| \| PANK3 \| Homo sapiens pantothenate kinase 3 (PANK3), mRNA. \| 1,53 \| \| --- \| Homo sapiens hypothetical LOC400590, mRNA (cDNA clone MGC:70830 IMAGE:5248762), complete cds. \| 1,52 \| \| SYVN1 \| Homo sapiens synovial apoptosis inhibitor 1, synoviolin (SYVN1), transcript variant 1, mRNA. \| 1,52 \| \| AGPS \| Homo sapiens alkylglycerone phosphate synthase (AGPS), mRNA. \| 1,52 \| \| RPP14 \| Homo sapiens ribonuclease P/MRP 14kDa subunit (RPP14), transcript variant 1, mRNA. \| 1,52 \| \| SLC9A7 \| Homo sapiens solute carrier family 9, subfamily A (NHE7, cation proton antiporter 7), member 7 (SLC9A7), transcript variant 1, mRNA. \| 1,52 \| \| PDIA3P1 \| Homo sapiens protein disulfide isomerase family A, member 3 pseudogene 1 (PDIA3P1), non-coding RNA. \| 1,52 \| \| SKIL \| Homo sapiens SKI-like proto-oncogene (SKIL), transcript variant 2, mRNA. \| 1,52 \| \| ZFAS1 \| Homo sapiens ZNFX1 antisense RNA 1 (ZFAS1), transcript variant 1, long non-coding RNA. \| 1,51 \| \| --- \| havana:known chromosome:GRCh38:14:52640839:52641566:1 gene:ENSG00000258757 gene_biotype:antisense transcript_biotype:antisense \| 1,51 \| \| --- \| mitochondrially encoded tRNA lysine [gene_biotype:Mt_tRNA transcript_biotype:Mt_tRNA] \| 1,51 \| \| RPF2 \| Homo sapiens ribosome production factor 2 homolog (RPF2), transcript variant 2, mRNA. \| 1,51 \| \| NAA35 \| Homo sapiens N(alpha)-acetyltransferase 35, NatC auxiliary subunit (NAA35), mRNA. \| 1,51 \| \| MIR3922 \| Homo sapiens microRNA 3922 (MIR3922), microRNA. \| 1,50 \| \| TAF1D \| TATA box binding protein (TBP)-associated factor, RNA polymerase I, D, 41kDa [gene_biotype:protein_coding transcript_biotype:nonsense_mediated_decay] \| 1,50 \| \| HIST2H2AB \| Homo sapiens histone cluster 2, H2ab (HIST2H2AB), mRNA. \| 1,50 \| \| CENPE \| Homo sapiens centromere protein E, 312kDa (CENPE), transcript variant 2, mRNA. \| 1,50 \|  \| **Pre-miR-29a** \| \| \| \| --- \| --- \| --- \| \| **Gene Symbol** \| **mRna - Description** \| **Fold Change** \| \| COL3A1 \| Homo sapiens collagen, type III, alpha 1 (COL3A1), mRNA. \| -3,69 \| \| INSIG1 \| Homo sapiens insulin induced gene 1 (INSIG1), transcript variant 1, mRNA. \| -3,33 \| \| SLC7A5 \| Homo sapiens solute carrier family 7 (amino acid transporter light chain, L system), member 5 (SLC7A5), mRNA. \| -3,00 \| \| COL5A2 \| Homo sapiens collagen, type V, alpha 2 (COL5A2), mRNA. \| -2,77 \| \| TGFB2 \| Homo sapiens transforming growth factor, beta 2 (TGFB2), transcript variant 1, mRNA. \| -2,56 \| \| KDELC1 \| Homo sapiens KDEL (Lys-Asp-Glu-Leu) containing 1 (KDELC1), mRNA. \| -2,56 \| \| MIR31HG \| Homo sapiens MIR31 host gene (MIR31HG), long non-coding RNA. \| -2,52 \| \| GXYLT2 \| Homo sapiens glucoside xylosyltransferase 2 (GXYLT2), mRNA. \| -2,51 \| \| FBN1 \| Homo sapiens fibrillin 1 (FBN1), mRNA. \| -2,49 \| \| COL6A3 \| Homo sapiens collagen, type VI, alpha 3 (COL6A3), transcript variant 1, mRNA. \| -2,42 \| \| MAP4K4 \| Homo sapiens mitogen-activated protein kinase kinase kinase kinase 4 (MAP4K4), transcript variant 4, mRNA. \| -2,40 \| \| MMP14 \| Homo sapiens matrix metallopeptidase 14 (membrane-inserted) (MMP14), mRNA. \| -2,38 \| \| COL1A2 \| Homo sapiens collagen, type I, alpha 2 (COL1A2), mRNA. \| -2,24 \| \| WNT5A \| Homo sapiens wingless-type MMTV integration site family, member 5A (WNT5A), transcript variant 2, mRNA. \| -2,21 \| \| SECTM1 \| Homo sapiens secreted and transmembrane 1 (SECTM1), mRNA. \| -2,14 \| \| PSAT1 \| Homo sapiens phosphoserine aminotransferase 1 (PSAT1), transcript variant 2, mRNA. \| -2,13 \| \| ARL4C \| Homo sapiens ADP-ribosylation factor-like 4C (ARL4C), transcript variant 1, mRNA. \| -2,11 \| \| AHR \| Homo sapiens aryl hydrocarbon receptor (AHR), mRNA. \| -2,08 \| \| PPIC \| Homo sapiens peptidylprolyl isomerase C (cyclophilin C) (PPIC), mRNA. \| -2,07 \| \| COL1A1 \| Homo sapiens collagen, type I, alpha 1 (COL1A1), mRNA. \| -2,06 \| \| IFI30 \| Homo sapiens interferon, gamma-inducible protein 30 (IFI30), mRNA. \| -2,06 \| \| GJA1 \| Homo sapiens gap junction protein, alpha 1, 43kDa (GJA1), mRNA. \| -2,06 \| \| CSGALNACT2 \| Homo sapiens chondroitin sulfate N-acetylgalactosaminyltransferase 2 (CSGALNACT2), mRNA. \| -2,03 \| \| --- \| havana:known chromosome:GRCh38:7:56603410:56603553:1 gene:ENSG00000271047 gene_biotype:processed_pseudogene transcript_biotype:processed_pseudogene \| -2,01 \| \| --- \| havana:known chromosome:GRCh38:7:56603410:56603553:1 gene:ENSG00000271047 gene_biotype:processed_pseudogene transcript_biotype:processed_pseudogene \| -2,01 \| \| --- \| havana:known chromosome:GRCh38:7:56603410:56603553:1 gene:ENSG00000271047 gene_biotype:processed_pseudogene transcript_biotype:processed_pseudogene \| -2,01 \| \| NID1 \| Homo sapiens nidogen 1 (NID1), mRNA. \| -2,00 \| \| UBE2CP5 \| Homo sapiens cDNA FLJ16160 fis, clone BRCAN2003070, moderately similar to Homo sapiens ubiquitin-conjugating enzyme E2C (UBE2C). \| -2,00 \| \| COL14A1 \| Homo sapiens collagen, type XIV, alpha 1 (COL14A1), mRNA. \| -2,00 \| \| TMEM158 \| Homo sapiens transmembrane protein 158 (gene/pseudogene) (TMEM158), mRNA. \| -1,99 \| \| FAM20C \| Homo sapiens family with sequence similarity 20, member C (FAM20C), mRNA. \| -1,95 \| \| CEMIP \| Homo sapiens cell migration inducing protein, hyaluronan binding (CEMIP), transcript variant 1, mRNA. \| -1,95 \| \| PLSCR4 \| Homo sapiens phospholipid scramblase 4 (PLSCR4), transcript variant 1, mRNA. \| -1,92 \| \| DPP4 \| Homo sapiens dipeptidyl-peptidase 4 (DPP4), mRNA. \| -1,91 \| \| SESTD1 \| Homo sapiens SEC14 and spectrin domains 1 (SESTD1), mRNA. \| -1,91 \| \| LACC1 \| Homo sapiens laccase (multicopper oxidoreductase) domain containing 1 (LACC1), transcript variant 1, mRNA. \| -1,89 \| \| LGR4 \| Homo sapiens leucine-rich repeat containing G protein-coupled receptor 4 (LGR4), mRNA. \| -1,89 \| \| --- \| Non-coding transcript identified by NONCODE: Antisense \| -1,89 \| \| VAMP7 \| Homo sapiens vesicle-associated membrane protein 7 (VAMP7), transcript variant 2, mRNA. \| -1,88 \| \| VAMP7 \| Homo sapiens vesicle-associated membrane protein 7 (VAMP7), transcript variant 2, mRNA. \| -1,86 \| \| LOC101928451 \| PREDICTED: Homo sapiens uncharacterized LOC101928451 (LOC101928451), transcript variant X2, misc_RNA. \| -1,84 \| \| HMOX1 \| Homo sapiens heme oxygenase 1 (HMOX1), mRNA. \| -1,83 \| \| LXN \| Homo sapiens latexin (LXN), mRNA. \| -1,82 \| \| CYP1B1 \| Homo sapiens cytochrome P450, family 1, subfamily B, polypeptide 1 (CYP1B1), mRNA. \| -1,82 \| \| EI24 \| Homo sapiens etoposide induced 2.4 (EI24), transcript variant 3, mRNA. \| -1,81 \| \| MARVELD1 \| Homo sapiens MARVEL domain containing 1 (MARVELD1), mRNA. \| -1,81 \| \| TMEM183B \| Homo sapiens transmembrane protein 183B (TMEM183B), mRNA. \| -1,80 \| \| MLLT11 \| Homo sapiens myeloid/lymphoid or mixed-lineage leukemia; translocated to, 11 (MLLT11), mRNA. \| -1,80 \| \| ANKRD50 \| Homo sapiens ankyrin repeat domain 50 (ANKRD50), transcript variant 2, mRNA. \| -1,79 \| \| ITGA5 \| Homo sapiens integrin, alpha 5 (fibronectin receptor, alpha polypeptide) (ITGA5), mRNA. \| -1,79 \| \| MALL \| Homo sapiens mal, T-cell differentiation protein-like (MALL), mRNA. \| -1,78 \| \| DBP \| Homo sapiens D site of albumin promoter (albumin D-box) binding protein (DBP), mRNA. \| -1,78 \| \| CHI3L1 \| Homo sapiens chitinase 3-like 1 (cartilage glycoprotein-39) (CHI3L1), mRNA. \| -1,77 \| \| --- \| Non-coding transcript identified by NONCODE \| -1,77 \| \| --- \| Non-coding transcript identified by NONCODE \| -1,77 \| \| ITGA11 \| Homo sapiens integrin, alpha 11 (ITGA11), mRNA. \| -1,77 \| \| S100A16 \| Homo sapiens S100 calcium binding protein A16 (S100A16), mRNA. \| -1,76 \| \| XYLT1 \| Homo sapiens xylosyltransferase I (XYLT1), mRNA. \| -1,76 \| \| PDGFRB \| Homo sapiens platelet-derived growth factor receptor, beta polypeptide (PDGFRB), mRNA. \| -1,76 \| \| MAZ \| Homo sapiens MYC-associated zinc finger protein (purine-binding transcription factor) (MAZ), transcript variant 2, mRNA. \| -1,74 \| \| POSTN \| Homo sapiens periostin, osteoblast specific factor (POSTN), transcript variant 2, mRNA. \| -1,74 \| \| --- \| Homo sapiens microRNA 423 (MIR423), microRNA. \| -1,73 \| \| --- \| Homo sapiens tripartite motif containing 25 (TRIM25), mRNA. \| -1,72 \| \| SPNS1 \| Homo sapiens spinster homolog 1 (Drosophila) (SPNS1), transcript variant 2, mRNA. \| -1,72 \| \| --- \| asparagine synthetase pseudogene 1[gene_biotype:unprocessed_pseudogene transcript_biotype:unprocessed_pseudogene] \| -1,72 \| \| SAT1 \| Homo sapiens spermidine/spermine N1-acetyltransferase 1 (SAT1), transcript variant 1, mRNA. \| -1,72 \| \| KIRREL \| Homo sapiens kin of IRRE like (Drosophila) (KIRREL), transcript variant 2, mRNA. \| -1,71 \| \| KLHDC3 \| Homo sapiens kelch domain containing 3 (KLHDC3), transcript variant 1, mRNA. \| -1,70 \| \| CHIC2 \| Homo sapiens cysteine-rich hydrophobic domain 2 (CHIC2), mRNA. \| -1,70 \| \| NDST1 \| Homo sapiens N-deacetylase/N-sulfotransferase (heparan glucosaminyl) 1 (NDST1), transcript variant 2, mRNA. \| -1,70 \| \| RAB27B \| Homo sapiens RAB27B, member RAS oncogene family (RAB27B), mRNA. \| -1,70 \| \| SEMA3C \| Homo sapiens sema domain, immunoglobulin domain (Ig), short basic domain, secreted, (semaphorin) 3C (SEMA3C), mRNA. \| -1,68 \| \| --- \| RNA, 5S ribosomal pseudogene 182 [gene_biotype:rRNA transcript_biotype:rRNA] \| -1,68 \| \| FAXDC2 \| Homo sapiens fatty acid hydroxylase domain containing 2 (FAXDC2), mRNA. \| -1,68 \| \| GPAT3 \| Homo sapiens glycerol-3-phosphate acyltransferase 3 (GPAT3), transcript variant 2, mRNA. \| -1,68 \| \| DKK1 \| Homo sapiens dickkopf WNT signaling pathway inhibitor 1 (DKK1), mRNA. \| -1,67 \| \| SNX9 \| Homo sapiens sorting nexin 9 (SNX9), mRNA. \| -1,67 \| \| RCC2 \| Homo sapiens regulator of chromosome condensation 2 (RCC2), transcript variant 2, mRNA. \| -1,67 \| \| SLC16A6 \| Homo sapiens solute carrier family 16, member 6 (SLC16A6), transcript variant 1, mRNA. \| -1,67 \| \| ID1 \| Homo sapiens inhibitor of DNA binding 1, dominant negative helix-loop-helix protein (ID1), transcript variant 1, mRNA. \| -1,66 \| \| FBXO32 \| Homo sapiens F-box protein 32 (FBXO32), transcript variant 3, mRNA. \| -1,66 \| \| SDC4 \| Homo sapiens syndecan 4 (SDC4), mRNA. \| -1,66 \| \| B4GALT1 \| Homo sapiens UDP-Gal:betaGlcNAc beta 1,4- galactosyltransferase, polypeptide 1 (B4GALT1), mRNA. \| -1,65 \| \| PTHLH \| Homo sapiens parathyroid hormone-like hormone (PTHLH), transcript variant 2, mRNA. \| -1,65 \| \| TRAM2 \| Homo sapiens translocation associated membrane protein 2 (TRAM2), mRNA. \| -1,65 \| \| ATP6AP1 \| Homo sapiens ATPase, H+ transporting, lysosomal accessory protein 1 (ATP6AP1), mRNA. \| -1,64 \| \| --- \| Homo sapiens chondroitin sulfate proteoglycan 4 (CSPG4), mRNA. \| -1,64 \| \| ATG9A \| Homo sapiens autophagy related 9A (ATG9A), transcript variant 1, mRNA. \| -1,64 \| \| PXDN \| Homo sapiens peroxidasin (PXDN), mRNA. \| -1,63 \| \| --- \| Non-coding transcript identified by NONCODE \| -1,63 \| \| SLC7A11 \| Homo sapiens solute carrier family 7 (anionic amino acid transporter light chain, xc- system), member 11 (SLC7A11), mRNA. \| -1,63 \| \| UACA \| Homo sapiens uveal autoantigen with coiled-coil domains and ankyrin repeats (UACA), transcript variant 2, mRNA. \| -1,63 \| \| --- \| Non-coding transcript identified by NONCODE \| -1,63 \| \| --- \| RNA, U6 small nuclear 892, pseudogene [gene_biotype:snRNA transcript_biotype:snRNA] \| -1,63 \| \| DYM \| Homo sapiens dymeclin (DYM), mRNA. \| -1,63 \| \| MIR378I \| Homo sapiens microRNA 378i (MIR378I), microRNA. \| -1,62 \| \| ITPRIP \| Homo sapiens inositol 1,4,5-trisphosphate receptor interacting protein (ITPRIP), transcript variant 2, mRNA. \| -1,62 \| \| SERPINB2 \| Homo sapiens serpin peptidase inhibitor, clade B (ovalbumin), member 2 (SERPINB2), transcript variant 1, mRNA. \| -1,61 \| \| NIPSNAP3A \| Homo sapiens nipsnap homolog 3A (C. elegans) (NIPSNAP3A), mRNA. \| -1,61 \| \| MIR1587 \| Homo sapiens microRNA 1587 (MIR1587), microRNA. \| -1,61 \| \| --- \| PREDICTED: Homo sapiens uncharacterized LOC101928451 (LOC101928451), transcript variant X1, misc_RNA. \| -1,61 \| \| SLC35G4 \| Homo sapiens solute carrier family 35, member G4 (SLC35G4), mRNA. \| -1,61 \| \| SPTLC3 \| Homo sapiens serine palmitoyltransferase, long chain base subunit 3 (SPTLC3), mRNA. \| -1,60 \| \| --- \| RNA, U6 small nuclear 628, pseudogene [gene_biotype:snRNA transcript_biotype:snRNA] \| -1,60 \| \| FAM129B \| Homo sapiens family with sequence similarity 129, member B (FAM129B), transcript variant 2, mRNA. \| -1,60 \| \| XPNPEP1 \| Homo sapiens X-prolyl aminopeptidase (aminopeptidase P) 1, soluble (XPNPEP1), transcript variant 2, mRNA. \| -1,60 \| \| MIR4710 \| Homo sapiens microRNA 4710 (MIR4710), microRNA. \| -1,60 \| \| --- \| Non-coding transcript identified by NONCODE \| -1,60 \| \| LOXL2 \| Homo sapiens lysyl oxidase-like 2 (LOXL2), mRNA. \| -1,59 \| \| --- \| Homo sapiens microRNA 4515 (MIR4515), microRNA. \| -1,59 \| \| PPT1 \| Homo sapiens palmitoyl-protein thioesterase 1 (PPT1), transcript variant 1, mRNA. \| -1,59 \| \| BPGM \| Homo sapiens 2,3-bisphosphoglycerate mutase (BPGM), transcript variant 3, mRNA. \| -1,59 \| \| --- \| RNA, 5S ribosomal pseudogene 199 [gene_biotype:rRNA transcript_biotype:rRNA] \| -1,58 \| \| --- \| Small nucleolar RNA U3 [gene_biotype:snoRNA transcript_biotype:snoRNA] \| -1,58 \| \| CD276 \| Homo sapiens CD276 molecule (CD276), transcript variant 1, mRNA. \| -1,57 \| \| GPRC5A \| Homo sapiens G protein-coupled receptor, class C, group 5, member A (GPRC5A), mRNA. \| -1,57 \| \| COL4A1 \| Homo sapiens collagen, type IV, alpha 1 (COL4A1), transcript variant 2, mRNA. \| -1,57 \| \| HGSNAT \| Homo sapiens heparan-alpha-glucosaminide N-acetyltransferase (HGSNAT), mRNA. \| -1,57 \| \| MGAT4B \| Homo sapiens mannosyl (alpha-1,3-)-glycoprotein beta-1,4-N-acetylglucosaminyltransferase, isozyme B (MGAT4B), transcript variant 1, mRNA. \| -1,57 \| \| LAMA3 \| Homo sapiens laminin, alpha 3 (LAMA3), transcript variant 2, mRNA. \| -1,57 \| \| SPRR2F \| Homo sapiens small proline-rich protein 2F (SPRR2F), mRNA. \| -1,57 \| \| SLC35B2 \| Homo sapiens solute carrier family 35 (adenosine 3-phospho 5-phosphosulfate transporter), member B2 (SLC35B2), transcript variant 2, mRNA. \| -1,56 \| \| PMP22 \| Homo sapiens peripheral myelin protein 22 (PMP22), transcript variant 1, mRNA. \| -1,56 \| \| NID2 \| Homo sapiens nidogen 2 (osteonidogen) (NID2), mRNA. \| -1,56 \| \| LINC00341 \| Homo sapiens long intergenic non-protein coding RNA 341 (LINC00341), long non-coding RNA. \| -1,56 \| \| LAMA4 \| Homo sapiens laminin, alpha 4 (LAMA4), transcript variant 1, mRNA. \| -1,56 \| \| NKIRAS2 \| Homo sapiens NFKB inhibitor interacting Ras-like 2 (NKIRAS2), transcript variant 1, mRNA. \| -1,56 \| \| --- \| Homo sapiens chondroitin sulfate proteoglycan 4 (CSPG4), mRNA. \| -1,56 \| \| LOC100101148 \| Homo sapiens FK506 binding protein 6, 36kDa pseudogene (LOC100101148), non-coding RNA. \| -1,56 \| \| STC1 \| Homo sapiens stanniocalcin 1 (STC1), mRNA. \| -1,56 \| \| NUPR1 \| Homo sapiens nuclear protein, transcriptional regulator, 1 (NUPR1), transcript variant 1, mRNA. \| -1,55 \| \| ANKRD28 \| Homo sapiens ankyrin repeat domain 28 (ANKRD28), transcript variant 2, mRNA. \| -1,55 \| \| FAM99B \| Homo sapiens family with sequence similarity 99, member B (non-protein coding) (FAM99B), long non-coding RNA. \| -1,55 \| \| PIGS \| Homo sapiens phosphatidylinositol glycan anchor biosynthesis, class S (PIGS), mRNA. \| -1,55 \| \| --- \| Non-coding transcript identified by NONCODE: Antisense \| -1,55 \| \| P4HA1 \| Homo sapiens prolyl 4-hydroxylase, alpha polypeptide I (P4HA1), transcript variant 1, mRNA. \| -1,55 \| \| BDKRB2 \| Homo sapiens bradykinin receptor B2 (BDKRB2), mRNA. \| -1,55 \| \| CCDC43 \| Homo sapiens coiled-coil domain containing 43 (CCDC43), transcript variant 2, mRNA. \| -1,54 \| \| ADAMTS5 \| Homo sapiens ADAM metallopeptidase with thrombospondin type 1 motif, 5 (ADAMTS5), mRNA. \| -1,54 \| \| IGLJ2 \| immunoglobulin lambda joining 2[gene_biotype:IG_gene transcript_biotype:IG_gene] \| -1,54 \| \| MIR4646 \| Homo sapiens microRNA 4646 (MIR4646), microRNA. \| -1,54 \| \| DSEL \| Homo sapiens dermatan sulfate epimerase-like (DSEL), mRNA. \| -1,54 \| \| --- \| PREDICTED: Homo sapiens major histocompatibility complex, class II, DR beta 4 (HLA-DRB4), transcript variant X1, mRNA. \| -1,54 \| \| ANKRD52 \| Homo sapiens ankyrin repeat domain 52 (ANKRD52), mRNA. \| -1,54 \| \| OSTC \| Homo sapiens oligosaccharyltransferase complex subunit (non-catalytic) (OSTC), transcript variant 2, mRNA. \| -1,54 \| \| LOC105372158 \| PREDICTED: Homo sapiens uncharacterized LOC105372158 (LOC105372158), transcript variant X1, ncRNA. \| -1,53 \| \| NPTX1 \| Homo sapiens neuronal pentraxin I (NPTX1), mRNA. \| -1,53 \| \| IFNA5 \| Homo sapiens interferon, alpha 5 (IFNA5), mRNA. \| -1,53 \| \| THRA \| Homo sapiens thyroid hormone receptor, alpha (THRA), transcript variant 3, mRNA. \| -1,53 \| \| IL1R1 \| Homo sapiens interleukin 1 receptor, type I (IL1R1), transcript variant 1, mRNA. \| -1,53 \| \| BICC1 \| Homo sapiens BicC family RNA binding protein 1 (BICC1), mRNA. \| -1,53 \| \| DYNLT1 \| Homo sapiens dynein, light chain, Tctex-type 1 (DYNLT1), transcript variant 2, mRNA. \| -1,53 \| \| YPEL3 \| Homo sapiens yippee-like 3 (YPEL3), transcript variant 2, mRNA. \| -1,53 \| \| C4orf32 \| Homo sapiens chromosome 4 open reading frame 32 (C4orf32), mRNA. \| -1,53 \| \| CATSPER2P1 \| Homo sapiens cation channel, sperm associated 2 pseudogene 1 (CATSPER2P1), non-coding RNA. \| -1,53 \| \| ABCC3 \| Homo sapiens ATP-binding cassette, sub-family C (CFTR/MRP), member 3 (ABCC3), transcript variant 2, mRNA. \| -1,52 \| \| TBC1D19 \| PREDICTED: Homo sapiens TBC1 domain family, member 19 (TBC1D19), transcript variant X2, mRNA. \| -1,52 \| \| COLGALT1 \| Homo sapiens collagen beta(1-O)galactosyltransferase 1 (COLGALT1), mRNA. \| -1,52 \| \| --- \| RNA, U6 small nuclear 1005, pseudogene [gene_biotype:snRNA transcript_biotype:snRNA] \| -1,52 \| \| --- \| linc-FAM153C-1 chr5:+:177384571-177386892 \| -1,52 \| \| --- \| Non-coding transcript identified by NONCODE: Linc \| -1,52 \| \| ABLIM1 \| PREDICTED: Homo sapiens actin binding LIM protein 1 (ABLIM1), transcript variant X8, mRNA. \| -1,52 \| \| UAP1L1 \| Homo sapiens UDP-N-acetylglucosamine pyrophosphorylase 1 like 1 (UAP1L1), mRNA. \| -1,52 \| \| MRFAP1 \| Homo sapiens Morf4 family associated protein 1 (MRFAP1), transcript variant 2, mRNA. \| -1,51 \| \| MT1X \| Homo sapiens metallothionein 1X (MT1X), mRNA. \| -1,51 \| \| SERPINH1 \| Homo sapiens serpin peptidase inhibitor, clade H (heat shock protein 47), member 1, (collagen binding protein 1) (SERPINH1), transcript variant 1, mRNA. \| -1,51 \| \| COL5A1 \| Homo sapiens collagen, type V, alpha 1 (COL5A1), transcript variant 1, mRNA. \| -1,51 \| \| --- \| RNA, 5S ribosomal pseudogene 64 [gene_biotype:rRNA transcript_biotype:rRNA] \| -1,51 \| \| --- \| macrophage stimulating 1 [gene_biotype:protein_coding transcript_biotype:retained_intron] \| -1,50 \| \| SPARC \| Homo sapiens secreted protein, acidic, cysteine-rich (osteonectin) (SPARC), transcript variant 2, mRNA. \| -1,50 \| |
| --- | --- | --- | --- | --- | --- | --- | --- | --- | --- | --- | --- | --- | --- | --- | --- | --- | --- | --- | --- | --- | --- | --- | --- | --- | --- | --- | --- | --- | --- | --- | --- | --- | --- | --- | --- | --- | --- | --- | --- | --- | --- | --- | --- | --- | --- | --- | --- | --- | --- | --- | --- | --- | --- | --- | --- | --- | --- | --- | --- | --- | --- | --- | --- | --- | --- | --- | --- | --- | --- | --- | --- | --- | --- | --- | --- | --- | --- | --- | --- | --- | --- | --- | --- | --- | --- | --- | --- | --- | --- | --- | --- | --- | --- | --- | --- | --- | --- | --- | --- | --- | --- | --- | --- | --- | --- | --- | --- | --- | --- | --- | --- | --- | --- | --- | --- | --- | --- | --- | --- | --- | --- | --- | --- | --- | --- | --- | --- | --- | --- | --- | --- | --- | --- | --- | --- | --- | --- | --- | --- | --- | --- | --- | --- | --- | --- | --- | --- | --- | --- | --- | --- | --- | --- | --- | --- | --- | --- | --- | --- | --- | --- | --- | --- | --- | --- | --- | --- | --- | --- | --- | --- | --- | --- | --- | --- | --- | --- | --- | --- | --- | --- | --- | --- | --- | --- | --- | --- | --- | --- | --- | --- | --- | --- | --- | --- | --- | --- | --- | --- | --- | --- | --- | --- | --- | --- | --- | --- | --- | --- | --- | --- | --- | --- | --- | --- | --- | --- | --- | --- | --- | --- | --- | --- | --- | --- | --- | --- | --- | --- | --- | --- | --- | --- | --- | --- | --- | --- | --- | --- | --- | --- | --- | --- | --- | --- | --- | --- | --- | --- | --- | --- | --- | --- | --- | --- | --- | --- | --- | --- | --- | --- | --- | --- | --- | --- | --- | --- | --- | --- | --- | --- | --- | --- | --- | --- | --- | --- | --- | --- | --- | --- | --- | --- | --- | --- | --- | --- | --- | --- | --- | --- | --- | --- | --- | --- | --- | --- | --- | --- | --- | --- | --- | --- | --- | --- | --- | --- | --- | --- | --- | --- | --- | --- | --- | --- | --- | --- | --- | --- | --- | --- | --- | --- | --- | --- | --- | --- | --- | --- | --- | --- | --- | --- | --- | --- | --- | --- | --- | --- | --- | --- | --- | --- | --- | --- | --- | --- | --- | --- | --- | --- | --- | --- | --- | --- | --- | --- | --- | --- | --- | --- | --- | --- | --- | --- | --- | --- | --- | --- | --- | --- | --- | --- | --- | --- | --- | --- | --- | --- | --- | --- | --- | --- | --- | --- | --- | --- | --- | --- | --- | --- | --- | --- | --- | --- | --- | --- | --- | --- | --- | --- | --- | --- | --- | --- | --- | --- | --- | --- | --- | --- | --- | --- | --- | --- | --- | --- | --- | --- | --- | --- | --- | --- | --- | --- | --- | --- | --- | --- | --- | --- | --- | --- | --- | --- | --- | --- | --- | --- | --- | --- | --- | --- | --- | --- | --- | --- | --- | --- | --- | --- | --- | --- | --- | --- | --- | --- | --- | --- | --- | --- | --- | --- | --- | --- | --- | --- | --- | --- | --- | --- | --- | --- | --- | --- | --- | --- | --- | --- | --- | --- | --- | --- | --- | --- | --- | --- | --- | --- | --- | --- | --- | --- | --- | --- | --- | --- | --- | --- | --- | --- | --- | --- | --- | --- | --- | --- | --- | --- | --- | --- | --- | --- | --- | --- | --- | --- | --- | --- | --- | --- | --- | --- | --- | --- | --- | --- | --- | --- | --- | --- | --- | --- | --- | --- | --- | --- | --- | --- | --- | --- | --- | --- | --- | --- | --- | --- | --- | --- | --- | --- | --- | --- | --- | --- | --- | --- | --- | --- | --- | --- | --- | --- | --- | --- | --- | --- | --- | --- | --- | --- | --- | --- | --- | --- | --- | --- | --- | --- | --- | --- | --- | --- | --- | --- | --- | --- | --- | --- | --- | --- | --- | --- | --- | --- | --- | --- | --- | --- | --- | --- | --- | --- | --- | --- | --- | --- | --- | --- | --- | --- | --- | --- | --- | --- | --- | --- | --- | --- | --- | --- | --- | --- | --- | --- | --- | --- | --- | --- | --- | --- | --- | --- | --- | --- | --- | --- | --- | --- | --- | --- | --- | --- | --- | --- | --- | --- | --- | --- | --- | --- | --- | --- | --- | --- | --- | --- | --- | --- | --- | --- | --- | --- | --- | --- | --- | --- | --- | --- | --- | --- | --- | --- | --- | --- | --- | --- | --- | --- | --- | --- | --- | --- | --- | --- | --- | --- | --- | --- | --- | --- | --- | --- | --- | --- | --- | --- | --- | --- | --- | --- | --- | --- | --- | --- | --- | --- | --- | --- | --- | --- | --- | --- | --- | --- | --- | --- | --- | --- | --- | --- | --- | --- | --- | --- | --- | --- | --- | --- | --- | --- | --- | --- | --- | --- | --- | --- | --- | --- | --- | --- | --- | --- | --- | --- | --- | --- | --- | --- | --- | --- | --- | --- | --- | --- | --- | --- | --- | --- | --- | --- | --- | --- | --- | --- | --- | --- | --- | --- | --- | --- | --- | --- | --- | --- | --- | --- | --- | --- | --- | --- | --- | --- | --- | --- | --- | --- | --- | --- | --- | --- | --- | --- | --- | --- | --- | --- | --- | --- | --- | --- | --- | --- | --- | --- | --- | --- | --- | --- | --- | --- | --- | --- | --- | --- | --- | --- | --- | --- | --- | --- | --- | --- | --- | --- | --- | --- | --- | --- | --- | --- | --- | --- | --- | --- | --- | --- | --- | --- | --- | --- | --- | --- | --- | --- | --- | --- | --- | --- | --- | --- | --- | --- | --- | --- | --- | --- | --- | --- | --- | --- | --- | --- | --- | --- | --- | --- | --- | --- | --- | --- | --- | --- | --- | --- | --- | --- | --- | --- | --- | --- | --- | --- | --- | --- | --- | --- | --- | --- | --- | --- | --- | --- | --- | --- | --- | --- | --- | --- | --- | --- | --- | --- | --- | --- | --- | --- | --- | --- | --- | --- | --- | --- | --- | --- | --- | --- | --- | --- | --- | --- | --- | --- | --- | --- | --- | --- | --- | --- | --- | --- | --- | --- | --- | --- | --- | --- | --- | --- | --- | --- | --- | --- | --- | --- | --- | --- | --- | --- | --- | --- | --- | --- | --- | --- | --- | --- | --- | --- | --- | --- | --- | --- | --- | --- | --- | --- | --- | --- | --- | --- | --- | --- | --- | --- | --- | --- | --- | --- | --- | --- | --- | --- | --- | --- | --- | --- | --- | --- | --- | --- | --- | --- | --- | --- | --- | --- | --- | --- | --- | --- | --- | --- | --- | --- | --- | --- | --- | --- | --- | --- | --- | --- | --- | --- | --- | --- | --- | --- | --- | --- | --- | --- | --- | --- | --- | --- | --- | --- | --- | --- | --- | --- | --- | --- | --- | --- | --- | --- | --- | --- | --- | --- | --- | --- | --- | --- | --- | --- | --- | --- | --- | --- | --- | --- | --- | --- | --- | --- | --- | --- | --- | --- | --- | --- | --- | --- | --- | --- | --- | --- | --- | --- | --- | --- | --- | --- | --- | --- | --- | --- | --- | --- | --- | --- | --- | --- | --- | --- | --- | --- | --- | --- | --- | --- | --- | --- | --- | --- | --- | --- | --- | --- | --- | --- | --- | --- | --- | --- | --- | --- | --- | --- | --- | --- | --- | --- | --- | --- | --- | --- | --- | --- | --- | --- | --- | --- | --- | --- | --- | --- | --- | --- | --- | --- | --- | --- | --- | --- | --- | --- | --- | --- | --- | --- | --- | --- | --- | --- | --- | --- | --- | --- | --- | --- | --- | --- | --- | --- | --- | --- | --- | --- | --- | --- | --- | --- | --- | --- | --- | --- | --- | --- | --- | --- | --- | --- | --- | --- | --- | --- | --- | --- | --- | --- | --- | --- | --- | --- | --- | --- | --- | --- | --- | --- | --- | --- | --- | --- | --- | --- | --- | --- | --- | --- | --- | --- | --- | --- | --- | --- | --- | --- | --- | --- | --- | --- | --- | --- | --- | --- | --- | --- | --- | --- | --- | --- | --- | --- | --- | --- | --- | --- | --- | --- | --- | --- | --- | --- | --- | --- | --- | --- | --- | --- | --- | --- | --- | --- | --- | --- | --- | --- | --- | --- | --- | --- | --- | --- | --- | --- | --- | --- | --- | --- | --- | --- | --- | --- | --- | --- | --- | --- | --- | --- | --- | --- | --- | --- | --- | --- | --- | --- | --- | --- | --- | --- | --- | --- | --- | --- | --- | --- | --- | --- | --- | --- | --- | --- | --- | --- | --- | --- | --- | --- | --- | --- | --- | --- | --- | --- | --- | --- | --- | --- | --- | --- | --- | --- | --- | --- | --- | --- | --- | --- | --- | --- | --- | --- | --- | --- | --- | --- | --- | --- | --- | --- | --- | --- | --- | --- | --- | --- | --- | --- | --- | --- | --- | --- | --- | --- | --- | --- | --- | --- | --- | --- | --- | --- | --- | --- | --- | --- | --- | --- | --- | --- | --- | --- | --- | --- | --- | --- | --- | --- | --- | --- | --- | --- | --- | --- | --- | --- | --- | --- | --- | --- | --- | --- | --- | --- | --- | --- | --- | --- | --- | --- | --- | --- | --- | --- | --- | --- | --- | --- | --- | --- | --- | --- | --- | --- | --- | --- | --- | --- | --- | --- | --- | --- | --- | --- | --- | --- | --- | --- | --- | --- | --- | --- | --- | --- | --- | --- | --- | --- | --- | --- | --- | --- | --- | --- | --- | --- | --- | --- | --- | --- | --- | --- | --- | --- | --- | --- | --- | --- | --- | --- | --- | --- | --- | --- | --- | --- | --- | --- | --- | --- | --- | --- | --- | --- | --- | --- | --- | --- | --- | --- | --- | --- | --- | --- | --- | --- | --- | --- | --- | --- | --- | --- | --- | --- | --- | --- | --- | --- | --- | --- | --- | --- | --- | --- | --- | --- | --- | --- | --- | --- | --- | --- | --- | --- | --- | --- | --- | --- | --- | --- | --- | --- | --- | --- | --- | --- | --- | --- | --- | --- | --- | --- | --- | --- | --- | --- | --- | --- | --- | --- | --- | --- | --- | --- | --- | --- | --- | --- | --- | --- | --- | --- | --- | --- | --- | --- | --- | --- | --- | --- | --- | --- | --- | --- | --- | --- | --- | --- | --- | --- | --- | --- | --- | --- | --- | --- | --- | --- | --- | --- | --- | --- | --- | --- | --- | --- | --- | --- | --- | --- | --- | --- | --- | --- | --- | --- | --- | --- | --- | --- | --- | --- | --- | --- | --- | --- | --- | --- | --- | --- | --- | --- | --- | --- | --- | --- | --- | --- | --- | --- | --- | --- | --- | --- | --- | --- | --- | --- | --- | --- | --- | --- | --- | --- | --- | --- | --- | --- | --- | --- | --- | --- | --- | --- | --- | --- | --- | --- | --- | --- | --- | --- | --- | --- | --- | --- | --- | --- | --- | --- | --- | --- | --- | --- | --- | --- | --- | --- | --- | --- | --- | --- | --- | --- | --- | --- | --- | --- | --- | --- | --- | --- | --- | --- | --- | --- | --- | --- | --- | --- | --- | --- | --- | --- | --- | --- | --- | --- | --- | --- | --- | --- | --- | --- | --- | --- | --- | --- | --- | --- | --- | --- | --- | --- | --- | --- | --- | --- | --- | --- | --- | --- | --- | --- | --- | --- | --- | --- | --- | --- | --- | --- | --- | --- | --- | --- | --- | --- | --- | --- | --- | --- | --- | --- | --- | --- | --- | --- | --- | --- | --- | --- | --- | --- | --- | --- | --- | --- | --- | --- | --- | --- | --- | --- | --- | --- | --- | --- | --- | --- | --- | --- | --- | --- | --- | --- | --- | --- | --- | --- | --- | --- | --- | --- | --- | --- | --- | --- | --- | --- | --- | --- | --- | --- | --- | --- | --- | --- | --- | --- | --- | --- | --- | --- | --- | --- | --- | --- | --- | --- | --- | --- | --- | --- | --- | --- | --- | --- | --- | --- | --- | --- | --- | --- | --- | --- | --- | --- | --- | --- | --- | --- | --- | --- | --- | --- | --- | --- | --- | --- | --- | --- | --- | --- | --- | --- | --- | --- | --- | --- | --- | --- | --- | --- | --- | --- | --- | --- | --- | --- | --- | --- | --- | --- | --- | --- | --- | --- | --- | --- | --- | --- | --- | --- | --- | --- | --- | --- | --- | --- | --- | --- | --- | --- | --- | --- | --- | --- | --- | --- | --- | --- | --- | --- | --- | --- | --- | --- | --- | --- | --- | --- | --- | --- | --- | --- | --- | --- | --- | --- | --- | --- | --- | --- | --- | --- | --- | --- | --- | --- | --- | --- | --- | --- | --- | --- | --- | --- | --- | --- | --- | --- | --- | --- | --- | --- | --- | --- | --- | --- | --- | --- | --- | --- | --- | --- | --- | --- | --- | --- | --- | --- | --- | --- | --- | --- | --- | --- | --- | --- | --- | --- | --- | --- | --- | --- | --- | --- | --- | --- | --- | --- | --- | --- | --- | --- | --- | --- | --- | --- | --- | --- | --- | --- | --- | --- | --- | --- | --- | --- | --- | --- | --- | --- | --- | --- | --- | --- | --- | --- | --- | --- | --- | --- | --- | --- | --- | --- | --- | --- | --- | --- | --- | --- | --- | --- | --- | --- | --- | --- | --- | --- | --- | --- | --- | --- | --- | --- | --- | --- | --- | --- | --- | --- | --- | --- | --- | --- | --- | --- | --- | --- | --- | --- | --- | --- | --- | --- | --- | --- | --- | --- | --- | --- | --- | --- | --- | --- | --- | --- | --- | --- | --- | --- | --- | --- | --- | --- | --- | --- | --- | --- | --- | --- | --- | --- | --- | --- | --- | --- | --- | --- | --- | --- | --- | --- | --- | --- | --- | --- | --- | --- | --- | --- | --- | --- | --- | --- | --- | --- | --- | --- | --- | --- | --- | --- | --- | --- | --- | --- | --- | --- | --- | --- | --- | --- | --- | --- | --- | --- | --- | --- | --- | --- | --- | --- | --- | --- | --- | --- | --- | --- | --- | --- | --- | --- | --- | --- | --- | --- | --- | --- | --- | --- | --- | --- | --- | --- | --- | --- | --- | --- | --- | --- | --- | --- | --- | --- | --- | --- | --- | --- | --- | --- | --- | --- | --- | --- | --- | --- | --- | --- | --- | --- | --- | --- | --- | --- | --- | --- | --- | --- | --- | --- | --- | --- | --- | --- | --- | --- | --- | --- | --- | --- | --- | --- | --- | --- | --- | --- | --- | --- | --- | --- | --- | --- | --- | --- | --- | --- | --- | --- | --- | --- | --- | --- | --- | --- | --- | --- | --- | --- | --- | --- | --- | --- | --- | --- | --- | --- | --- | --- | --- | --- | --- | --- | --- | --- | --- | --- | --- | --- | --- | --- | --- | --- | --- | --- | --- | --- | --- | --- | --- | --- | --- | --- | --- | --- | --- | --- | --- | --- | --- | --- | --- | --- | --- | --- | --- | --- | --- | --- | --- | --- | --- | --- | --- | --- | --- | --- | --- | --- | --- | --- | --- | --- | --- | --- | --- | --- | --- | --- | --- | --- | --- | --- | --- | --- | --- | --- | --- | --- | --- | --- | --- | --- | --- | --- | --- | --- | --- | --- | --- | --- | --- | --- | --- | --- | --- | --- | --- | --- | --- | --- | --- | --- | --- | --- | --- | --- | --- | --- | --- | --- | --- | --- | --- | --- | --- | --- | --- | --- | --- | --- | --- | --- | --- | --- | --- | --- | --- | --- | --- | --- | --- | --- | --- | --- | --- | --- | --- | --- | --- | --- | --- | --- | --- | --- | --- | --- | --- | --- | --- | --- | --- | --- | --- | --- | --- | --- | --- | --- | --- | --- | --- | --- | --- | --- | --- | --- | --- | --- | --- | --- | --- | --- | --- | --- | --- | --- | --- | --- | --- | --- | --- | --- | --- | --- | --- | --- | --- | --- | --- | --- | --- | --- | --- | --- | --- | --- | --- | --- | --- | --- | --- | --- | --- | --- | --- | --- | --- | --- | --- | --- | --- | --- | --- | --- | --- | --- | --- | --- | --- | --- | --- | --- | --- | --- | --- | --- | --- | --- | --- | --- | --- | --- | --- | --- | --- | --- | --- | --- | --- | --- | --- | --- | --- | --- | --- | --- | --- | --- | --- | --- | --- | --- | --- | --- | --- | --- | --- | --- | --- | --- | --- | --- | --- | --- | --- | --- | --- | --- | --- | --- | --- | --- | --- | --- | --- | --- | --- | --- | --- | --- | --- | --- | --- | --- | --- | --- | --- | --- | --- | --- | --- | --- | --- | --- | --- | --- | --- | --- | --- | --- | --- | --- | --- | --- | --- | --- | --- | --- | --- | --- | --- | --- | --- | --- | --- | --- | --- | --- | --- | --- | --- | --- | --- | --- | --- | --- | --- | --- | --- | --- | --- | --- | --- | --- | --- | --- | --- | --- | --- | --- | --- | --- | --- | --- | --- | --- | --- | --- | --- | --- | --- | --- | --- | --- | --- | --- | --- | --- | --- | --- | --- | --- | --- | --- | --- | --- | --- | --- | --- | --- | --- | --- | --- | --- | --- | --- | --- | --- | --- | --- | --- | --- | --- | --- | --- | --- | --- | --- | --- | --- | --- | --- | --- | --- | --- | --- | --- | --- | --- | --- | --- | --- | --- | --- | --- | --- | --- | --- | --- | --- | --- | --- | --- | --- | --- | --- | --- | --- | --- | --- | --- | --- | --- | --- | --- | --- | --- | --- | --- | --- | --- | --- | --- | --- | --- | --- | --- | --- | --- | --- | --- | --- | --- | --- | --- | --- | --- | --- | --- | --- | --- | --- | --- | --- | --- | --- | --- | --- | --- | --- | --- | --- | --- | --- | --- | --- | --- | --- | --- | --- | --- | --- | --- | --- | --- | --- | --- | --- | --- | --- | --- | --- | --- | --- | --- | --- | --- | --- | --- | --- | --- | --- | --- | --- | --- | --- | --- | --- | --- | --- | --- | --- | --- | --- | --- | --- | --- | --- | --- | --- | --- | --- | --- | --- | --- | --- | --- | --- | --- | --- | --- | --- | --- | --- | --- | --- | --- | --- | --- | --- | --- | --- | --- | --- | --- | --- | --- | --- | --- | --- | --- | --- | --- | --- | --- | --- | --- | --- | --- | --- | --- | --- | --- | --- | --- | --- | --- | --- | --- | --- | --- | --- | --- | --- | --- | --- | --- | --- | --- | --- | --- | --- | --- | --- | --- | --- | --- | --- | --- | --- | --- | --- | --- | --- | --- | --- | --- | --- | --- | --- | --- | --- | --- | --- | --- | --- | --- | --- | --- | --- | --- | --- | --- | --- | --- | --- | --- | --- | --- | --- | --- | --- | --- | --- | --- | --- | --- | --- | --- | --- | --- | --- | --- | --- | --- | --- | --- | --- | --- | --- | --- | --- | --- | --- | --- | --- | --- | --- | --- | --- | --- | --- | --- | --- | --- | --- | --- | --- | --- | --- | --- | --- | --- | --- | --- | --- | --- | --- | --- | --- | --- | --- | --- | --- | --- | --- | --- | --- | --- | --- | --- | --- | --- | --- | --- | --- | --- | --- | --- | --- | --- | --- | --- | --- | --- | --- | --- | --- | --- | --- | --- | --- | --- | --- | --- | --- | --- | --- | --- | --- | --- | --- | --- | --- | --- | --- | --- | --- | --- | --- | --- | --- | --- | --- | --- | --- | --- | --- | --- | --- | --- | --- | --- | --- | --- | --- | --- | --- | --- | --- | --- | --- | --- | --- | --- | --- | --- | --- | --- | --- | --- | --- | --- | --- | --- | --- | --- | --- | --- | --- | --- | --- | --- | --- | --- | --- | --- | --- | --- | --- | --- | --- | --- | --- | --- | --- | --- | --- | --- | --- | --- | --- | --- | --- | --- | --- | --- | --- | --- | --- | --- | --- | --- | --- | --- | --- | --- | --- | --- | --- | --- | --- | --- | --- | --- | --- | --- | --- | --- | --- | --- | --- | --- | --- | --- | --- | --- | --- | --- | --- | --- | --- | --- | --- | --- | --- | --- | --- | --- | --- | --- | --- | --- | --- | --- | --- | --- | --- | --- | --- | --- | --- | --- | --- | --- | --- | --- | --- | --- | --- | --- | --- | --- | --- | --- | --- | --- | --- | --- | --- | --- | --- | --- | --- | --- | --- | --- | --- | --- | --- | --- | --- | --- | --- | --- | --- | --- | --- | --- | --- | --- | --- | --- | --- | --- | --- | --- | --- | --- | --- | --- | --- | --- | --- | --- | --- | --- | --- | --- | --- | --- | --- | --- | --- | --- | --- | --- | --- | --- | --- | --- | --- | --- | --- | --- | --- | --- | --- | --- | --- | --- | --- | --- | --- | --- | --- | --- | --- | --- | --- | --- | --- | --- | --- | --- | --- | --- | --- | --- | --- | --- | --- | --- | --- | --- | --- | --- | --- | --- | --- | --- | --- | --- | --- | --- | --- | --- | --- | --- | --- | --- | --- | --- | --- | --- | --- | --- | --- | --- | --- | --- | --- | --- | --- | --- | --- | --- | --- | --- | --- | --- | --- | --- | --- | --- | --- | --- | --- | --- | --- | --- | --- | --- | --- | --- | --- | --- | --- | --- | --- | --- | --- | --- | --- | --- | --- | --- | --- | --- | --- | --- | --- | --- | --- | --- | --- | --- | --- | --- | --- | --- | --- | --- | --- | --- | --- | --- | --- | --- | --- | --- | --- | --- | --- | --- | --- | --- | --- | --- | --- | --- | --- | --- | --- | --- | --- | --- | --- | --- | --- | --- | --- | --- | --- | --- | --- | --- | --- | --- | --- | --- | --- | --- | --- | --- | --- | --- | --- | --- | --- | --- | --- | --- | --- | --- | --- | --- | --- | --- | --- | --- | --- | --- | --- | --- | --- | --- | --- | --- | --- | --- | --- | --- | --- | --- | --- | --- | --- | --- | --- | --- | --- | --- | --- | --- | --- | --- | --- | --- | --- | --- | --- | --- | --- | --- | --- | --- | --- | --- | --- | --- | --- | --- | --- | --- | --- | --- | --- | --- | --- | --- | --- | --- | --- | --- | --- | --- | --- | --- | --- | --- | --- | --- | --- | --- | --- | --- | --- | --- | --- | --- | --- | --- | --- | --- | --- | --- | --- | --- | --- | --- | --- | --- | --- | --- | --- | --- | --- | --- | --- | --- | --- | --- | --- | --- | --- | --- | --- | --- | --- | --- | --- | --- | --- | --- | --- | --- | --- | --- | --- | --- | --- | --- | --- | --- | --- | --- | --- | --- | --- | --- | --- | --- | --- | --- | --- | --- | --- | --- | --- | --- | --- | --- | --- | --- | --- | --- | --- | --- | --- | --- | --- | --- | --- | --- | --- | --- | --- | --- | --- | --- | --- | --- | --- | --- | --- | --- | --- | --- | --- | --- | --- | --- | --- | --- | --- | --- | --- | --- | --- | --- | --- | --- | --- | --- | --- | --- | --- | --- | --- | --- | --- | --- | --- | --- | --- | --- | --- | --- | --- | --- | --- | --- | --- | --- | --- | --- | --- | --- | --- | --- | --- | --- | --- | --- | --- | --- | --- | --- | --- | --- | --- | --- | --- | --- | --- | --- | --- | --- | --- | --- | --- | --- | --- | --- | --- | --- | --- | --- | --- | --- | --- | --- | --- | --- | --- | --- | --- | --- | --- | --- | --- | --- | --- | --- | --- | --- | --- | --- | --- | --- | --- | --- | --- | --- | --- | --- | --- | --- | --- | --- | --- | --- | --- | --- | --- | --- | --- | --- | --- | --- | --- | --- | --- | --- | --- | --- | --- | --- | --- | --- | --- | --- | --- | --- | --- | --- | --- | --- | --- | --- | --- | --- | --- | --- | --- | --- | --- | --- | --- | --- | --- | --- | --- | --- | --- | --- | --- | --- | --- | --- | --- | --- | --- | --- | --- | --- | --- | --- | --- | --- | --- | --- | --- | --- | --- | --- | --- | --- | --- | --- | --- | --- | --- | --- | --- | --- | --- | --- | --- | --- | --- | --- | --- | --- | --- | --- | --- | --- | --- | --- | --- | --- | --- | --- | --- | --- | --- | --- | --- | --- | --- | --- | --- | --- | --- | --- | --- | --- | --- | --- | --- | --- | --- | --- | --- | --- | --- | --- | --- | --- | --- | --- | --- | --- | --- | --- | --- | --- | --- | --- | --- | --- | --- | --- | --- | --- | --- | --- | --- | --- | --- | --- | --- | --- | --- | --- | --- | --- | --- | --- | --- | --- | --- | --- | --- | --- | --- | --- | --- | --- | --- | --- | --- | --- | --- | --- | --- | --- | --- | --- | --- | --- | --- | --- | --- | --- | --- | --- | --- | --- | --- | --- | --- | --- | --- | --- | --- | --- | --- | --- | --- | --- | --- | --- | --- | --- | --- | --- | --- | --- | --- | --- | --- | --- | --- | --- | --- | --- | --- | --- | --- | --- | --- | --- | --- | --- | --- | --- | --- | --- | --- | --- | --- | --- | --- | --- | --- | --- | --- | --- | --- | --- | --- | --- | --- | --- | --- | --- | --- | --- | --- | --- | --- | --- | --- | --- | --- | --- | --- | --- | --- | --- | --- | --- | --- | --- | --- | --- | --- | --- | --- | --- | --- | --- | --- | --- | --- | --- | --- | --- | --- | --- | --- | --- | --- | --- | --- | --- | --- |
