# Supplementary material for: miR-155 Contributes to the Immunoregulatory Function of Human Mesenchymal Stem Cells
Source: Front Immunol. 2021 Mar 26;12:624024. doi: 10.3389/fimmu.2021.624024 (PMC8033167; doi:10.3389/fimmu.2021.624024)
Supplement: Supplementary file 5 [file Table_5.docx]

S**upl. Table 5:** List of target genes modulated in PBMCs after cocuture with premiR-155 overexpressing MSCs or PHA activation. Values are expressed as the ratio of the relative gene expression in PBMCs cultured with premiR-155 overexpressing MSCs (p155) on premiR-control overexpressing MSCs (pCT) or, in PHA-activated PBMCs (A) on non-activated PBMCs (NA).

| **Gene** | **p155/pCT ratio** | **Gene** | **A/NA ratio** |  | **Gene** | **p155/pCT ratio** | **Gene** | **A/NA ratio** |
| --- | --- | --- | --- | --- | --- | --- | --- | --- |
| MAFB | 1,69 | MYB | 20,00 |  | DET1 | 1,12 | UQCR11 | 2,25 |
| BCORL1 | 1,59 | ASTN2 | 13,95 |  | FOXO3 | 1,11 | H3-3A | 2,24 |
| SPI1 | 1,56 | AICDA | 6,45 |  | SOCS1 | 1,09 | TTL | 2,20 |
| HNRNPA3 | 1,41 | MSH2 | 6,37 |  | BACH1 | 1,08 | RAP1B | 2,20 |
| NFATC2IP | 1,33 | RUNX2 | 5,27 |  | ZNF236 | 1,07 | HIVEP2 | 2,17 |
| STX16 | 1,32 | MSH6 | 4,72 |  | FADD | 1,07 | RHOA | 2,10 |
| CEBPB | 1,32 | ETS1 | 3,93 |  | KAT2A | 1,07 | TAB2 | 1,97 |
| RUNX2 | 1,31 | JARID2 | 3,41 |  | PAM | 1,06 | HNRNPA3 | 1,87 |
| TP53INP1 | 1,30 | MLH1 | 3,36 |  | TTL | 1,06 | INPP5D | 1,85 |
| AAK1 | 1,29 | DHX40 | 3,27 |  | LDOC1 | 1,06 | ARID2 | 1,80 |
| RIPK1 | 1,28 | MATR3 | 3,11 |  | TAB2 | 1,04 | TLE4 | 1,80 |
| INPP5D | 1,27 | BCORL1 | 3,06 |  | CHD9 | 1,04 | STX16 | 1,74 |
| JADE1 | 1,27 | SMAD2 | 3,05 |  | SEPT11 | 1,04 | MECP2 | 1,73 |
| RREB1 | 1,27 | CARD11 | 2,96 |  | NDFIP1 | 1,04 | CSNK1G2 | 1,68 |
| IRAK3 | 1,24 | SKIV2L2 | 2,90 |  | MSH2 | 1,03 | PAM | 1,65 |
| TLE4 | 1,24 | APC | 2,85 |  | MYB | 1,02 | TM6SF1 | 1,63 |
| SKI | 1,24 | USP14 | 2,83 |  | USP14 | 1,02 | FOXO3 | 1,60 |
| ZNF652 | 1,23 | G3BP2 | 2,78 |  | MSH6 | 1,01 | RIPK1 | 1,52 |
| ARID2 | 1,22 | LDOC1 | 2,78 |  | SKIV2L2 | 1,01 | SMAD5 | 1,38 |
| CARD11 | 1,22 | SMAD1 | 2,78 |  | LPAR6 | 1,00 | DET1 | 1,29 |
| EDN1 | 1,22 | NFATC2IP | 2,77 |  | MLH1 | 1,00 | IKBKE | 1,21 |
| SMAD1 | 1,21 | TCEB1 | 2,75 |  | G3BP2 | 1,00 | MEF2A | 1,08 |
| KRAS | 1,21 | AAK1 | 2,75 |  | TOMM20 | 1,00 | SKI | 1,05 |
| MEF2A | 1,20 | ILF3 | 2,70 |  | INFGR1 | 1,00 | CHD9 | 0,86 |
| CSNK1G2 | 1,19 | ZMYM2 | 2,63 |  | TM6SF1 | 0,98 | RREB1 | 0,80 |
| ASTN2 | 1,19 | SOCS1 | 2,63 |  | DHX40 | 0,98 | BACH1 | 0,76 |
| TSHZ3 | 1,19 | JADE1 | 2,60 |  | MATR3 | 0,97 | ZNF652 | 0,71 |
| ETS1 | 1,17 | TP53INP1 | 2,53 |  | AICDA | 0,97 | INFGR1 | 0,67 |
| ILF3 | 1,16 | KAT2A | 2,48 |  | TCEB1 | 0,96 | LPAR6 | 0,39 |
| MECP2 | 1,16 | NDFIP1 | 2,44 |  | RAP1B | 0,95 | IRAK3 | 0,29 |
| JARID2 | 1,16 | KRAS | 2,37 |  | UQCR11 | 0,94 | CEBPB | 0,24 |
| IKBKE | 1,15 | SEPT11 | 2,37 |  | RHOA | 0,93 | TSHZ3 | 0,22 |
| ZMYM2 | 1,15 | ZNF236 | 2,35 |  | SMAD2 | 0,93 | MAFB | 0,13 |
| SMAD5 | 1,14 | FADD | 2,34 |  | H3-3A | 0,92 | EDN1 | 0,13 |
| HIVEP2 | 1,14 | PELI1 | 2,31 |  | APC | 0,85 | SPI1 | 0,07 |
| PELI1 | 1,14 | TOMM20 | 2,31 |  |  |  |  |  |
